# Chemical composition and larvicidal activities of *Azolla pinnata* extracts against *Aedes* (Diptera:Culicidae)

**DOI:** 10.1371/journal.pone.0206982

**Published:** 2018-11-06

**Authors:** Rajiv Ravi, Nor Shaida Husna Zulkrnin, Nurul Nadiah Rozhan, Nik Raihan Nik Yusoff, Mohd Sukhairi Mat Rasat, Muhammad Iqbal Ahmad, Intan H. Ishak, Mohamad Faiz Mohd Amin

**Affiliations:** 1 Faculty of Earth Science, Universiti Malaysia Kelantan, Jeli Campus, Jeli, Kelantan, Malaysia; 2 Faculty of Bioengineering and Technology, Universiti Malaysia Kelantan, Jeli Campus, Jeli, Kelantan, Malaysia; 3 School of Biological Sciences, Universiti Sains Malaysia, Minden, Penang, Malaysia; 4 Vector Control Research Unit, School of Biological Sciences, Universiti Sains Malaysia, Minden, Penang, Malaysia; Banaras Hindu University, INDIA

## Abstract

**Background:**

The resistance problem of dengue vectors to different classes of insecticides that are used for public health has raised concerns about vector control programmes. Hence, the discovery of alternative compounds that would enhance existing tools is important for overcoming the resistance problem of using insecticides in vectors and ensuring a chemical-free environment. The larvicidal effects of *Azolla pinnata* extracts by using two different extraction methods with methanol solvent against *Aedes* in early 4^th^ instar larvae was conducted.

**Methods:**

The fresh *Azolla pinnata* plant from Kuala Krai, Kelantan, Malaysia was used for crude extraction using Soxhlet and maceration methods. Then, the chemical composition of extracts and its structure were identified using GCMS-QP2010 Ultra (Shimadzu). Next, following the WHO procedures for larval bioassays, the extracts were used to evaluate the early 4^th^ instar larvae of *Aedes* mosquito vectors.

**Results:**

The larvicidal activity of *Azolla pinnata* plant extracts evidently affected the early 4^th^ instar larvae of *Aedes aegypti* mosquito vectors. The Soxhlet extraction method had the highest larvicidal effect against *Ae*. *aegypti* early 4^th^ instar larvae, with LC50 and LC95 values of 1093 and 1343 mg/L, respectively. Meanwhile, the maceration extraction compounds were recorded with the LC50 and LC95 values of 1280 and 1520 mg/L, respectively. The larvae bioassay test for *Ae*. *albopictus* showed closely similar values in its Soxhlet extraction, with LC50 and LC95 values of 1035 and 1524 mg/L, compared with the maceration extraction LC50 and LC95 values of 1037 and 1579 mg/L, respectively. The non-target organism test on guppy fish, *Poecilia reticulata*, showed no mortalities and posed no toxic effects. The chemical composition of the *Azolla pinnata* plant extract has been found and characterized as having 18 active compounds for the Soxhlet method and 15 active compounds for the maceration method.

**Conclusions:**

Our findings showed that the crude extract of *A*. *pinnata* bioactive molecules are effective and have the potential to be developed as biolarvicides for *Aedes* mosquito vector control. This study recommends future research on the use of active ingredients isolated from *A*. *pinnata* extracts and their evaluation against larvicidal activity of *Aedes* in small-scale field trials for environmentally safe botanical insecticide invention.

## Background

Mosquitoes are a harrowing nuisance and are primary vectors for vector-borne diseases that affects humans [1]. One of the most important viral infections for humans is dengue, which is transmitted by bites of infected *Aedes* mosquitoes, and it is considered to be a major health problem in tropical and subtropical countries [2]. Currently, Malaysia has recorded 55,744 dengue cases, with 131 deaths between January and July 2017 [3]. The worldwide distribution of dengue epidemics includes 124 countries, and 3.61 billion people are at risk of being infected, with 500 million at risk of infection each year [4].

Currently, only physical and chemical methods are commonly used to control mosquito-borne diseases. Physical approaches, such as bed nets and covering the human body with light-coloured clothes, are only temporary solutions. Meanwhile, the use of chemical methods, such as the application of temephos and pyrethroids, is more prominent but comes with resistant challenges. In Malaysia, resistance evidence towards permethrin and temephos has been recorded from *Ae*. *aegypti* in Kuala Lumpur and Penang regions [5,6]. Ishak et al. [7], stated that insecticide resistances were caused by two main factors: the increase in the rate of insecticide metabolism and alterations in its target sites.

Chemical insecticides have improved biological activity that results from synergistic effects of active or individually inactive compounds and the explanatory effects of structurally related compounds that counter resistance development, which characterizes most single-component bioactive compounds of the current mosquitocide classes [8–11]. Additionally, Ghosh et al. [12] supported the concepts of botanical control mechanisms for a simple and sustainable method compared to conventional insecticides. Unlike conventional insecticides, the advantage of plant-derived insecticides composed by botanical blends of multiple chemical compounds is that they may act concertedly on both physiological and behavioural processes. Hence, alternative uses of bio-insecticides would provide a more suitable and sustainable solution against *Ae*. *aegypti and Ae*. *albopictus*. Following this safer and greener alternative concept, the *Azolla pinnata* plant has potential as a bio-insecticide to solve the problems of resistance from a single chemical compound.

*Azolla pinnata* is also commonly known as a mosquito weed that has been used for the nourishment of paddy growth, because it provides the required nitrogen source. Moreover, *A*. *pinnata* also forms a thick mat on the water surface, which may prevent breeding and adult mosquito emergence by covering the surface of sluggish, still or stagnant freshwater bodies, hence preventing adult mosquitoes from laying eggs and reducing the emergence and development of mosquito larvae [13]. Meanwhile, Larvicides [14] reported in a field study that the breeding of malaria-transmitting mosquitoes was almost completely suppressed in pools, wells and ponds that were covered with the *A*. *pinnata* plant. In the paddy fields of Tanzania, Africa, Kusumawathie et al. [15] found that *Anabaena azollae* reduced the larval productivity and larval densities of *An*. *Gambiae*, *An*. *funestus* and *Cx*. *quinquefasciatus*. Kusumawathie et al. [15] suggested that the mosquito productivity is low when the Azolla coverage is high (>80%) in paddy fields. Understanding these potentials from *A*. *pinnata*, Ekanayake et al. [16] evaluated the phytochemical properties. The *A*. *pinnata* compounds were characterized as alkaloids, flavonoids, phenols, saponins, quinones, tannins, carboxylic acids, proteins, xanthoproteins, coumarins, steroids and carbohydrates [16]. Despite many studies on the *A*. *pinnata* plant against mosquitoes, it appears that all of the literatures only focused on the physical barrier of these plants, and none conducted a study on its biochemical application for mosquitocidal and larvicidal effects. Hence, there are no other studies on the specific chemical compound and its structure for larvicidal effects. Additionally, no other explicit study has stated the suitable extraction method for the *A*. *pinnata* plant. Various extraction methods have been deployed to exploit the plant material resources and to obtain its valuable compounds [17]. However, the extraction relies on mainly the temperature and the solubilization of metabolites, depending on the plant species and its reliability of thermo-stable chemical compounds [18]. Therefore, the right method is necessary to extract the desired chemical components from the plant for its further application.

Despite the fact that there are many studies on the *Azolla* plant with mosquitoes, none of them have mentioned the specific bioactive compounds and crude plant applications against *Aedes* mosquitoes. To date, no other studies have been done on the identification of the specific chemical compound structure of the *A*. *pinnata* crude extract and its applications against the larvicides of *Ae*. *aegypti* and *Ae*. *albopictus*. Additionally, this study will also compare the efficacies between two extractions methods of *A*. *pinnata* crude extracts against the larvicides of *Ae*. *aegypti* and *Ae*. *albopictus*. Hence, the purpose of this study is to identify the chemical compounds and their structures from maceration and Soxhlet extraction methods for the *A*. *pinnata* plant and to test their efficacies by comparing the two extraction methods against the early 4^th^ instar larvae of *Ae*. *aegypti* and *Ae*. *albopictus*.

## Materials and methods

All experimental procedures were approved by animal ethics: USM/IACUC/2018/111/909 from Vector Control Research Unit, School of Biological Sciences, Universiti Sains Malaysia, Minden, Penang, Malaysia.

### Plant materials

**Fig 1:**
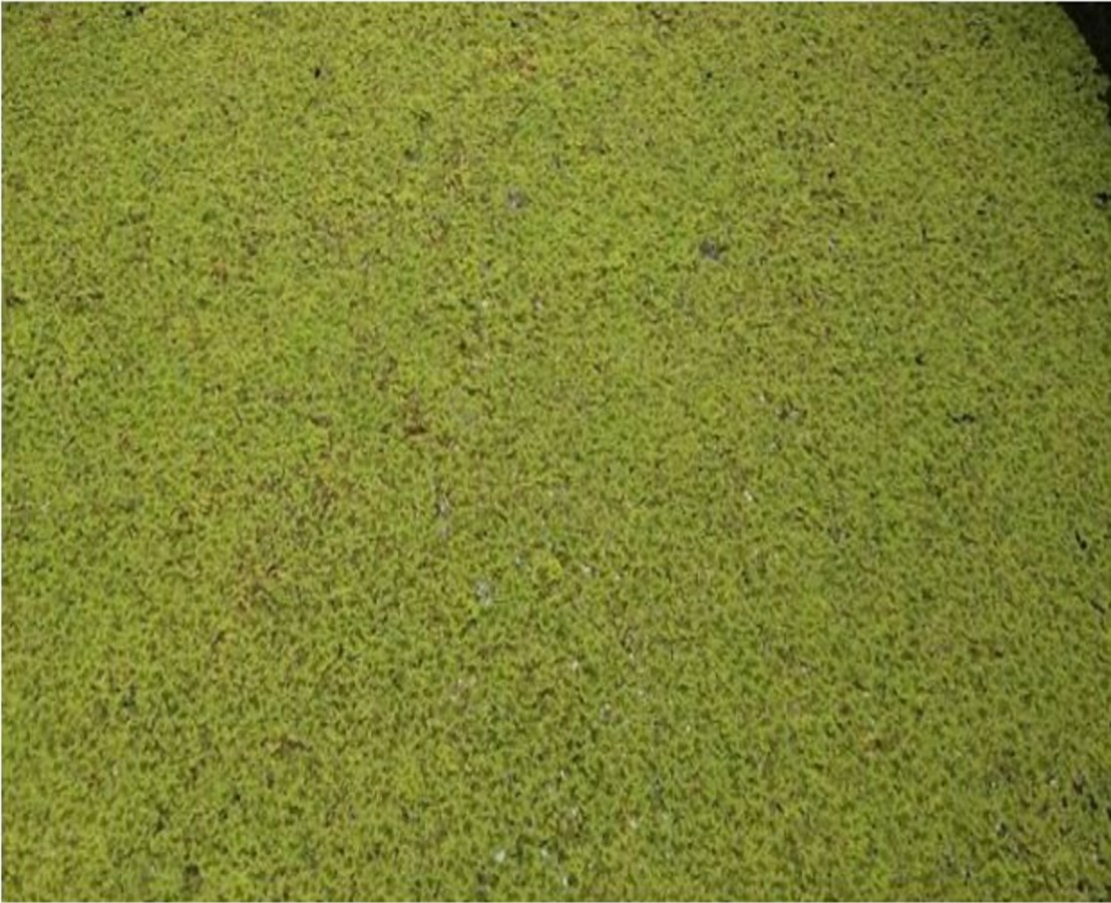
Picture of *Azolla pinnata* plant from the field. The Ventral view of phyllotaxis from *Azolla pinnata* plant.

A total of 50 kg fresh *A*. *pinnata* (Fig 1) was sampled from Kuala Krai, Kelantan (5° 31’ N 102° 12’ E) and its species was identified based on a morphological view of phyllotaxis. *A*. *pinnata* fresh samples were prepared using a sun-dried (30°C ± 4°C room temperature) technique for 2 days. Then, the dried samples were powdered electrically with a grinding stainless steel blender (Faber brand, model: FBG-460K) and were sieved as fine powder. The fine powders would increase the surface area and thus increase the rate of extractions [19]. Next, two different extraction methods were used in this study: maceration and Soxhlet, using methanol as a solvent.

### Maceration extraction

According to Atanasova et al. [20], a total of 60 g of the dried whole plant of *A*. *pinnata* powder was measured and placed in a beaker; then, one litre of methanol solvent was added into the beaker. Furthermore, the samples were left for 7 days until the plant cellular structure was penetrated and softened by solvents. The plant sample in the solvent was occasionally stirred to facilitate the extraction. After completing that procedure, the extract was then filtered with Whatman filter paper No. 1 to remove the sample waste and then was evaporated in a rotary vacuum evaporator to obtain the crude extract. Finally, the crude extract powders were kept at -20°C until further use.

### Soxhlet extraction

Following Atanasova et al. [20], Soxhlet extraction was performed using a Soxhlet apparatus (Favorit, Malaysia) with 40 g of dried plant powder, placed into the paper thimble. Next, some cotton wool was placed on the top part of the extraction flask to prevent the sample from overflowing onto other apparatus parts. One litre of methanol solvent was placed in a round-bottom flask with the heating mantle underneath. The solvent was heated by refluxing it repeatedly and was then washed with fine-ground plant material to extract the desired compound into the round-bottom flask. The extraction in the Soxhlet apparatus was at boiling point of 70°C for approximately 3 hours until the solvent in the siphon arm became clear, which indicated that the sample was extracted entirely. The extracts were evaporated to dryness in the vacuum evaporator to obtain the crude extract. Finally, the crude extract powders were kept at -4°C until further use.

### GC-MS analysis

The GC-MS analysis of the maceration and Soxhlet methods, which were used to obtain crude extracts from *A*. *pinnata*, was performed on a GCMS-QP2010 Ultra (Shimadzu). We followed the method used by previously published research findings of plant extracts [21]. The GCMS-QP2010 Ultra (Shimadzu) system, fitted with a BPX5capillary column (30 m×0.25 mm inner diameter, ×0.25μm film thickness; maximum temperature, 370°C), coupled to a QP2010 Ultra (Shimadzu) MS. Ultra-high purity helium (99.99%) was used as carrier gas at a constant flow rate of 1.0 mL/ min. The injection, transfer line and ion source temperatures were all 280°C. The oven temperature was programmed from 80°C (hold for 2 min) to 280°C at a rate of 3°C/min. The crude samples were diluted with the appropriate solvent (1/100, v/v) and filtered. The particle-free diluted crude extracts (1 μL) were taken in a syringe and injected into an injector with a split ratio of 10:1. All data were obtained by collecting the full-scan mass spectra within the scan range of 40–550 amu. The percentage composition of the crude extract constituents was expressed as the percentage by peak area. The identification and characterization of chemical compounds in various crude extracts were based on the GC retention time. The mass spectra were computer matched with those of the standards available in the NIST 08 mass spectrum libraries.

### Larvae rearing

The eggs of *Aedes* were obtained from the Vector Control Research Unit (VCRU) at the University Sains Malaysia (USM), Penang, Malaysia. We followed the method used by [13, 22] in larvae rearing. The eggs were hatched in de-chlorinated water for 24 hours and were maintained at 25°C to 30°C (room temperature), a pH of 6.95 to 7.03, relative humidity of 80 ± 10% and dissolved oxygen from 5.5 to 6.1 mg/L in the laboratory. After five days, the early 4^th^ instar larvae were used for the bioassay test.

### Larvicidal bioassay

Larvicidal bioassays were performed in accordance with the standard World Health Organization (2005) guidelines [14]. Bioassays were carried out using 25, *Ae*. *aegypti* and *Ae*. *albopictus* to early 4^th^ instar larvae (homogeneous population consisting 5 mm to 6 mm in body length). The bioassays were replicated four times using 25 larvae for each concentration, with methanol CH_3_OH as a solvent for the control. During the larval testing period, fish meal was provided. Initially, before selecting an accurate testing dose, all of the larvae were subjected to a wide range of test concentrations. This step is necessary to determine the range of extract solutions for larvicidal activities [13]. In this study, seven concentrations ranging from 500 mg/L to 2500 mg/L, yielding between 0 and 100% mortality in 24 hours of exposure, were selected as test concentrations. The control solutions were prepared with 1 ml of distilled water and 10% of the respective methanol solvent for each of the experimental replicates [13]. The reason for using solvent control is to ensure that all test replicates are identical to the plant extract solutions and to ensure that the mortality results were not due to its solvent [13]. The experiments were conducted at room temperature 28±2°C, and larvae mortalities were recorded at intervals of 24 hours and 48 hours [14]. Immobilization and total absence from the larvae, even after touch, was the end point of the bioassay [23]. The data were analysed by using a probit analysis in IBM SPSS Statistics 24 [13].

### Morphological view

*Ae*. *aegypti* early 4^th^ instar larvae were observed under an optical microscope (Leica USA), with a magnification of 40-400x [24].

### Non-targeted organism test

Guppy fish, *Poecilia reticulata*, were used for the non-target organism test, with a total of ten fishes in three replicates with a 1.20g mean weight and 3.5cm mean length (acclimatization period of 12 days in laboratory conditions before the start of experiment). Each treatment group were tested with a larvicidal LC95 plant extract concentration dissolved in 2000 mL anti-chlorinated water. A control group was also set with only 2000 mL of anti-chlorine treated water. The tests were performed for 24 hours with observations made during the first 10 minutes, then at 1, 2, 3, 6 and 24 hours. The mortality and observable abnormalities of the fish were recorded. The test conditions of water, such as the pH, water temperature and dissolved oxygen, were recorded during the start and end of the experiment [15,16].

## Results

### GC-MS analysis and identification of compounds

#### Soxhlet extraction

**Fig 2:**
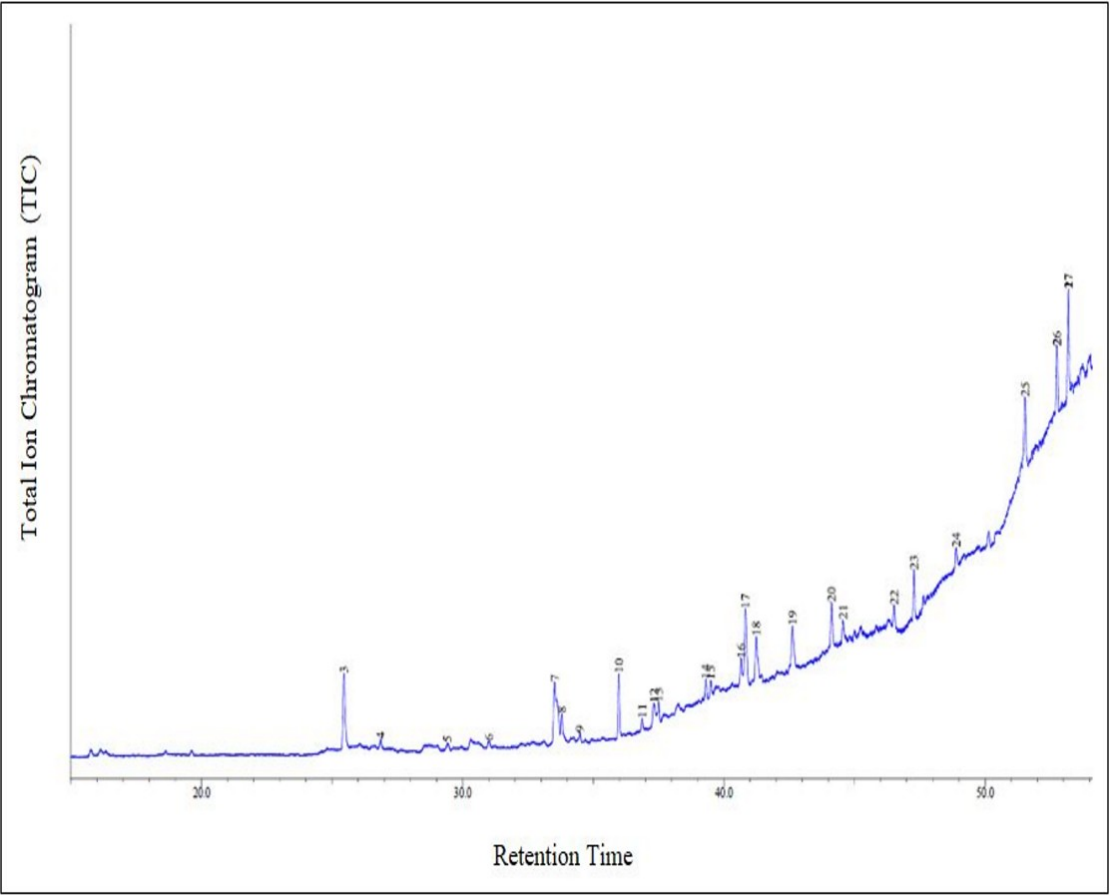
Chromatogram for GC-MS analysis of methanol extract using soxhlet extraction method for *A*. *Pinnata* (ACQUISITION PARAMETERS; BPX5capillary column 30 m×0.25 mm inner diameter, ×0.25 μm film thickness, the oven temperature was programmed from 80°C (hold for 2 min) to 280°C at a rate of 3°C/min Carrier Gas = He).

The GC-MS analysis of methanol solvent extracts using the Soxhlet extraction of *A*. *Pinnata* showed 27 peaks, which indicated the presence of 27 phytochemical compounds (Fig 2). In the comparison of the mass spectra of the constituents with the NIST 08 library, only 17 compounds were characterized and identified (Table 1). The major 5 highest peak chemical compounds in the extracts were diethyl phthalate (20.449%), Bis(2-ethylhexyl) methylphosphonate (17.960%), sulfurous acid, cyclohexylmethyl pentadecyl ester (17.038%), methacrylic acid, dodecyl ester (8.305%), and 2,4,4,6,6,8,8-heptamethyl-1-nonene (8.529%). The attached supplementary, S1 and S2 Tables pdf file contains the NIST 08 library search for chemical compound structures and details.

**Table 1:** Soxhlet extracted chemical compounds for *Azolla Pinnata*.

S/N	RT	Area	Area %	Compound Name	Activity
3	25.453	625738	20.449	Diethyl Phthalate	Insecticidal activity[25,26]
5	29.416	67674	2.212	Sulfurous acid, cyclohexylmethyl tridecyl ester	Pesticides activity[27]
6	31.003	39128	1.279	Nonane 2,2,4,4,6,8,8-heptamethyl-	Pesticides activity[28]
7	33.521	254140	8.305	Methacrylic acid, dodecyl ester	Pigments, lubricant additives in industry[29]
9	34.484	7854	0.257	1-Nonadecene	Insecticidal and anticancer activity[30]
10	35.978	72998	2.386	Neophytadiene	Larvicidal, insecticidal and antimicrobial activity[31]
13	37.506	18733	0.612	3,7,11,15-Tetramethyl-2-hexadecen-1-ol	Insecticidal, anti-parasitic, nematicide and antimicrobial activity[32]
14	39.308	47654	1.557	Hexadecanoic acid, methyl ester	Insecticidal, nematicide, pesticide activity[33]
15	39.497	30916	1.010	Benzenepropanoic acid, 3,5-bis(1,1-dimethylethyl)-4-	Antioxidant activity[34]
17	40.822	157771	5.156	Methacrylic acid, pentadecyl ester	Antimicrobial activity[34]
18	41.236	521365	17.038	Sulfurous acid, cyclohexylmethyl pentadecyl ester	Pesticide activity[35]
19	42.624	260983	8.529	2,4,4,6,6,8,8-Heptamethyl-1-nonene	Antioxidant activity[36]
21	44.560	39256	1.283	Behenic alcohol	Pesticide, agrochemical lubricants, emulsifiers, insecticides, and detergent activity[37]
23	47.281	69797	2.281	Methacrylic acid, hexadecyl ester	Pesticide activity[38]
24	48.891	160457	5.244	Sulfurous acid, cyclohexylmethyl pentadecyl ester	Pesticide activity[38]
25	51.529	549563	17.960	Bis(2-ethylhexyl) methylphosphonate	Pesticide activity[39]
27	53.186	135898	4.441	Methacrylic acid, heptadecyl ester	Pesticide activity[39]

Note: S/N: Signal Noise, RT: Retention Time

#### Maceration extraction

**Fig 3:**
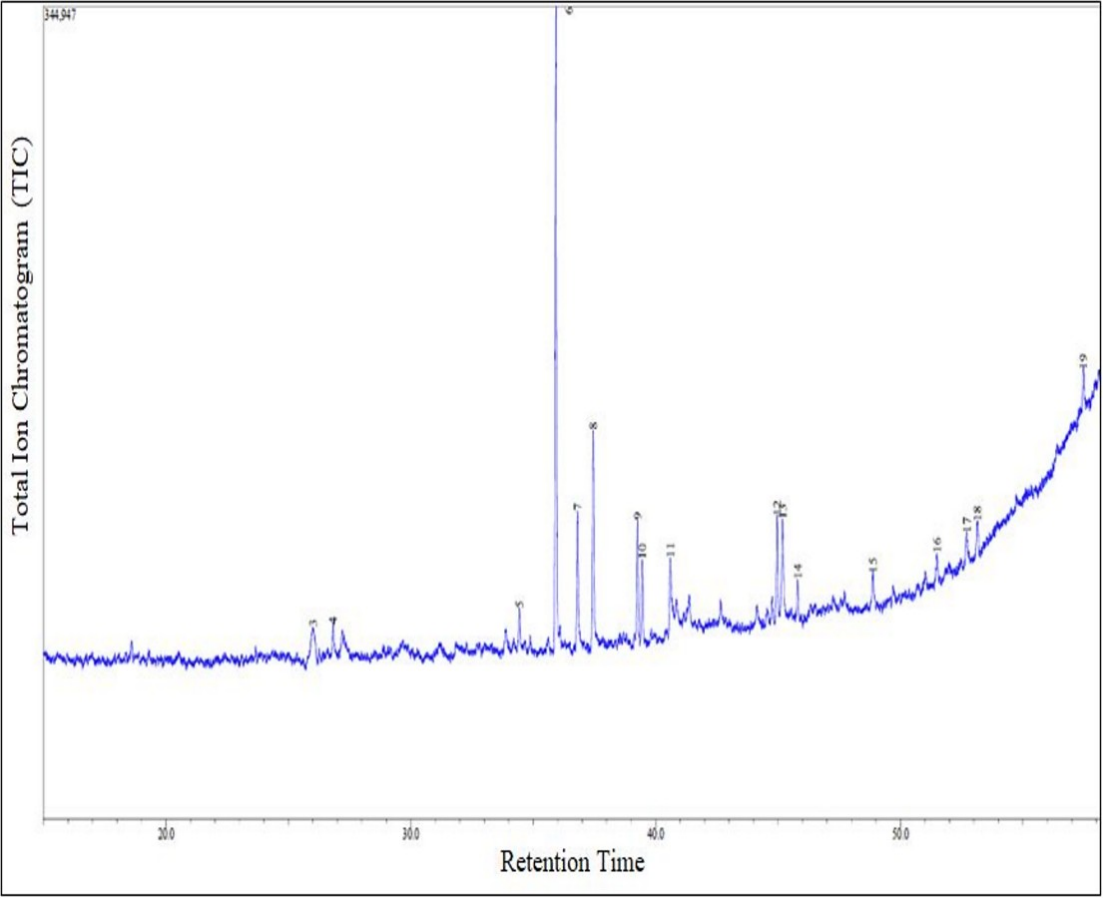
Chromatogram for GC-MS analysis of methanol extract using maceration extraction method for *A*. *Pinnata* (ACQUISITION PARAMETERS; BPX5 capillary column 30 m×0.25 mm inner diameter, ×0.25 μm film thickness, the oven temperature was programmed from 80°C (hold for 2 min) to 280°C at a rate of 3°C/min Carrier Gas = He).

The GC-MS analysis of methanol solvent extracts using the maceration extraction of *A*. *pinnata* showed 19 peaks that indicated the presence of 19 phytochemical compounds (Fig 3). In the comparison of the mass spectra of the constituents with the NIST 08 library, only 15 compounds were characterized and identified (Table 2). The major 5 highest peak chemical compounds in the extracts were neophytadiene (27.852%), hexadecanoate <methyl> (14.182%), phytol (10.043%), 2,4,4,6,6,8,8-heptamethyl-2-nonene (9.507%), 3,7,11,15-tetramethyl-2-hexadecen-1-ol (9.061%). The attached supplementary, S1 and S2 Tables pdf file contains the NIST 08 library search for chemical compound structures and details.

**Table 2:** Maceration extracted chemical compound for *Azolla Pinnata*.

S/N	RT	Area	Area %	Compound Name	Activity
1	3.623	1176	0.347	1-Methyldecylamine	Insecticidal activity[39]
4	26.824	4307	1.270	1-Heptadecene	Insecticidal and antibacterial activity[40,41]
5	34.437	6599	1.946	1-Nonadecene	Insecticidal and fungizide activity[42–44]
6	35.926	94443	27.853	Neophytadiene	Larvicidal, insecticidal and antimicrobial activity[31,45,46]
8	37.446	30724	9.061	3,7,11,15-Tetramethyl-2-hexadecen-1-ol	Insecticidal, anti-parasitic, nematicide and antimicrobial activity[32,47,48]
9	39.260	48088	14.182	Hexadecanoate <methyl>	Insecticidal and pesticide activity[21]
10	39.451	24512	7.229	Benzenepropanoic acid, 3,5-bis(1,1-dimethylethyl)-4-	Antioxidant activity[34]
11	40.592	12072	3.560	Hexadecanoic acid <n->	Insecticidal and pesticide activity[49]
12	44.965	11534	3.402	9-Octadecenoic acid, methyl ester, (E)-	Insecticidal and antifeedant activity[45]
13	45.188	34055	10.043	Phytol	Insecticidal, fungicide, miticide activity[9]
14	45.796	12993	3.832	Octadecanoic acid, methyl ester	Anti-inflammatory, antimicrobial, pesticide activity[50]
16	51.487	32238	9.507	2,4,4,6,6,8,8-Heptamethyl-2-nonene	Biodegradable activity[51]
17	52.704	15078	4.447	2,4,4,6,6,8,8-Heptamethyl-1-nonene	Antioxidant activity[52]
18	53.141	6765	1.995	Methacrylic acid, heptadecyl ester	Pesticide activity[38]
19	57.475	4498	1.327	16-Octadecenal	Surfactant, emulsifier industry activity[53]

Note: S/N: Signal Noise, RT: Retention Time

### Larvicidal bioassay

**Fig 4:**
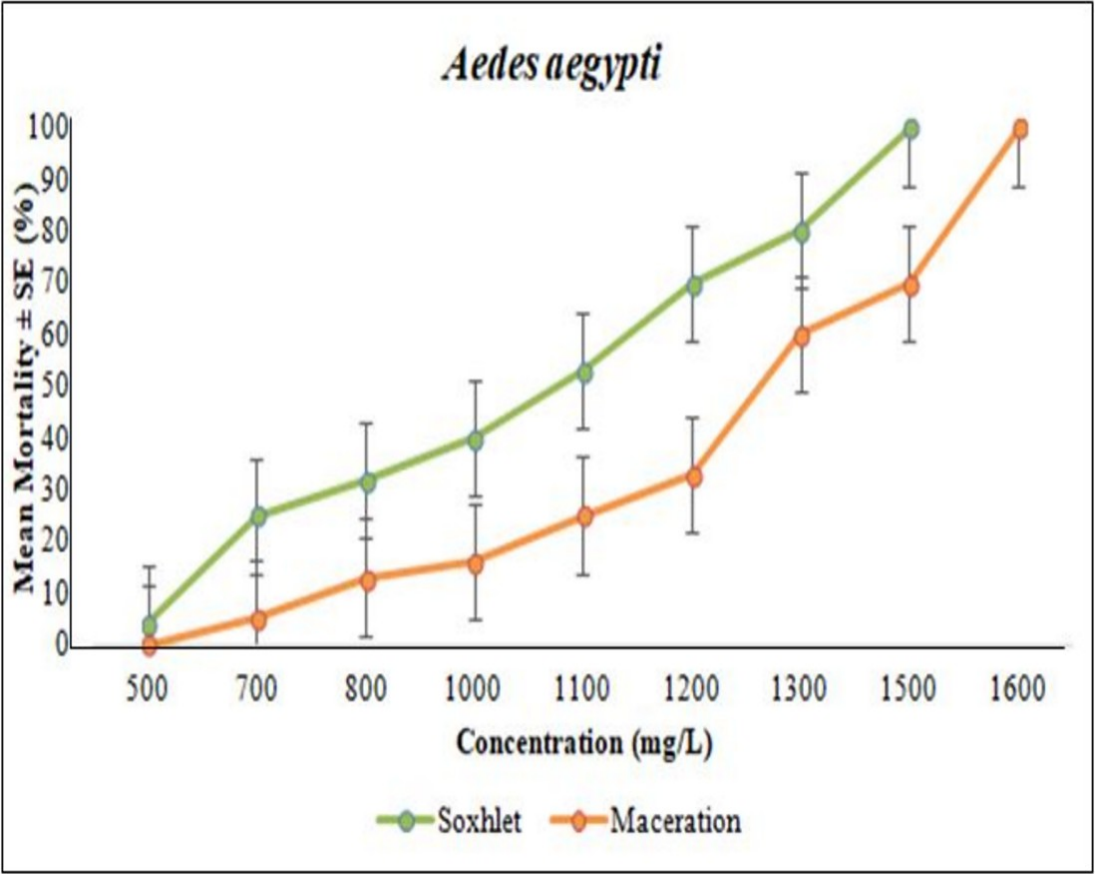
Comparison of *Aedes aegypti* larvae mortality rate between soxhlet and maceration for various *Azolla pinnata* extract concentrations.

**Fig 5:**
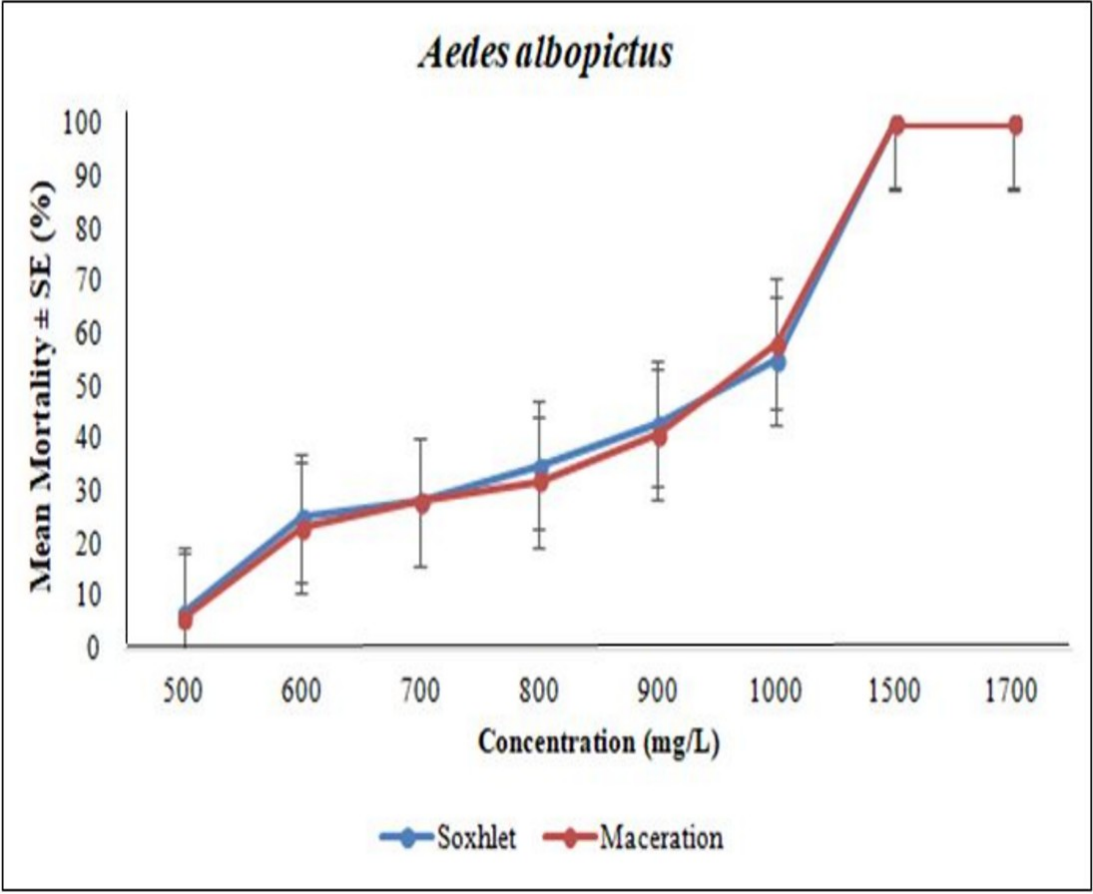
Comparison of *Aedes albopictus* larvae mortality rate between soxhlet and maceration for various *Azolla pinnata* extract concentrations.

**Fig 6:**
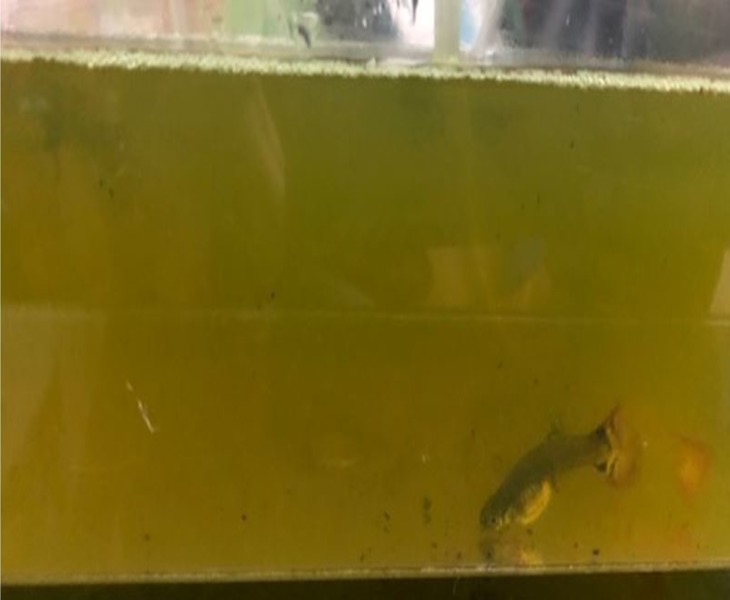
Guppy fish, *Poecilia reticulata* toxicity test with *Azolla pinnata* extracts.

The bioassay testing from the Soxhlet extraction method of *A*. *pinnata* was tested at 500 mg/L, 700 mg/L, 800 mg/L, 1000 mg/L, 1100 mg/L, 1200 mg/L, 1300 mg/L and 1500 mg/L; meanwhile, the maceration extraction method was tested at 500 mg/L, 700 mg/L, 800 mg/L, 1000 mg/L, 1100 mg/L, 1200 mg/L, 1300 mg/L, 1500 mg/L and 1600 mg/L. The entire larvae bioassay test with *A*. *pinnata* extracts showed a significant increase in the mortality percentage with the increase of concentration. Among the plant extracts tested, the highest larvicidal activity was observed in the Soxhlet-extracted compounds against the early 4^th^ instar larvae of *Ae*. *Aegypti*, with the LC50 and LC95 values of 1093 and 1343 mg/L, respectively (Table 3). Meanwhile, the maceration extraction compounds were recorded with the LC50 and LC95 values of 1280 and 1520 mg/L, respectively (Table 3). The larvae bioassay test for *Ae*. *albopictus* showed closely similar values in its Soxhlet extraction LC50 and LC95 values of 1035 and 1524 mg/L, respectively, and with maceration extraction LC50 and LC95 values of 1037 and 1579 mg/L, respectively (Table 4). Figs 4 and 5 show the graphical representation of the larvae mortality rate between Soxhlet and maceration methods. Finally, the results for the non-target organism test on guppy fish, *Poecilia reticulata*, showed no mortalities with plant extracts at 1500 mg/L and possessed no toxic effects on fish (Fig 6). The 95% confidence limits LC50 (95%CI) and LC95 (95%CI), chi-square and the degree of freedom (df) values were also calculated (Table 3). In the control assay, there was no significant mortality.

**Table 3:** Larvicidal activity of *Azolla pinnata* extracts against early 4^th^ instar larvae of *Ae*. *Aegypti*.

Extraction Method	N_a_	LC_50_(mg/L)(95% LCL-UCL)	LC_95_(mg/L)(95% LCL-UCL)	*X*^2^	df	R
Maceration	100	1280(1119–1387)Y = -21.871+7.038X	1520(1408–1600)Y = -21.871+7.038X	30*	12	0.986
Soxhlet	100	1093(1038–1151)Y = -22.890+7.533X	1343(1266–1453)Y = -22.890+7.533X	30*	16	0.979

N_a_; total number of mosquitoes larvae used; n = 25 with 4 replicates

LC50; Lethal concentration 50% mortality, LC95; Lethal concentration 95% mortality

LCL;lower confidence limits, UCL; upper confidence limits

(χ2); Pearson chi square, df; degrees of freedom, R; Pearson’s R

(Note: Chi-square values with asterisk"*" are significant P<0.05).

**Table 4:** Larvicidal activity of *Azolla pinnata* extracts against early 4^th^ instar larvae of *Ae*. *Albopictus*.

Extraction Method	N_a_	LC_50_(mg/L)(95% LCL-UCL)	LC_95_(mg/L)(95% LCL-UCL)	*X*^2^	df	R
Maceration	100	1037(983–1109)Y = -19.196+6.644X	1579(1426–1813)Y = -19.196+6.644X	40*	18	0.987
Soxhlet	100	1035(980–1107)Y = -20.607+6.876X	1524(1377–1764)Y = -20.607+6.876X	40*	15	0.988

N_a_; total number of mosquitoes larvae used; n = 25 with 4 replicates

LC50; Lethal concentration 50% mortality, LC95; Lethal concentration 95% mortality

LCL;lower confidence limits, UCL; upper confidence limits

(χ2); Pearson chi square, df; degrees of freedom, R; Pearson’s R

(Note: Chi-square values with asterisk "*"are significant P<0.05).

All the analysis data are available in S3, S4, S5, S6, S7 and S8 Tables

### Morphological view

**Fig 7:**
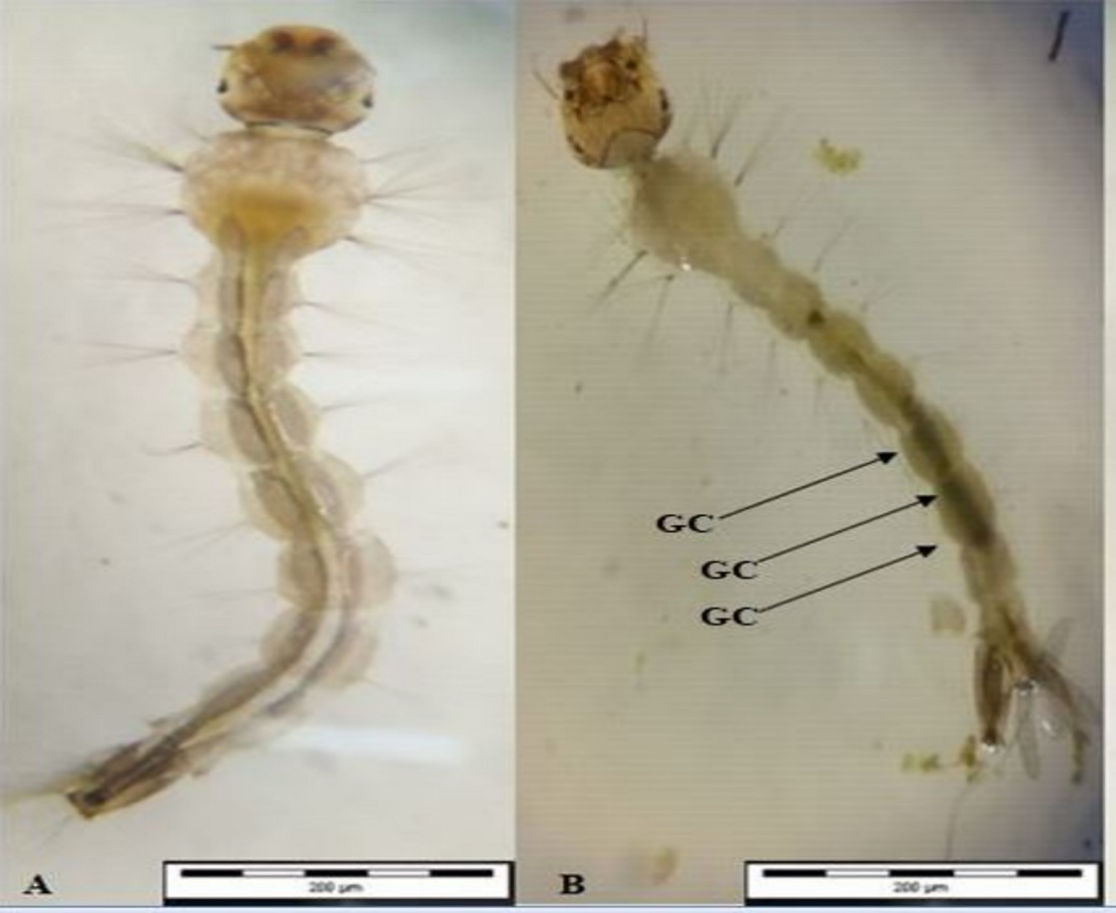
Morphological midgut content induced by *Azolla pinnata* plant extract from soxhlet extraction method in larvae of *Ae*. *Aegypti*. (A) Control test for midgut content view in early 4^th^ instar larvae of *Ae*. *Aegypti* (B) *A*. *pinnata* crude extract for midgut content view in larvae of *Ae*. *Aegypti* Note: Arrows indicating the plant extracts (greenish colour), GC: gut content (after 24hours).

**Fig 8:**
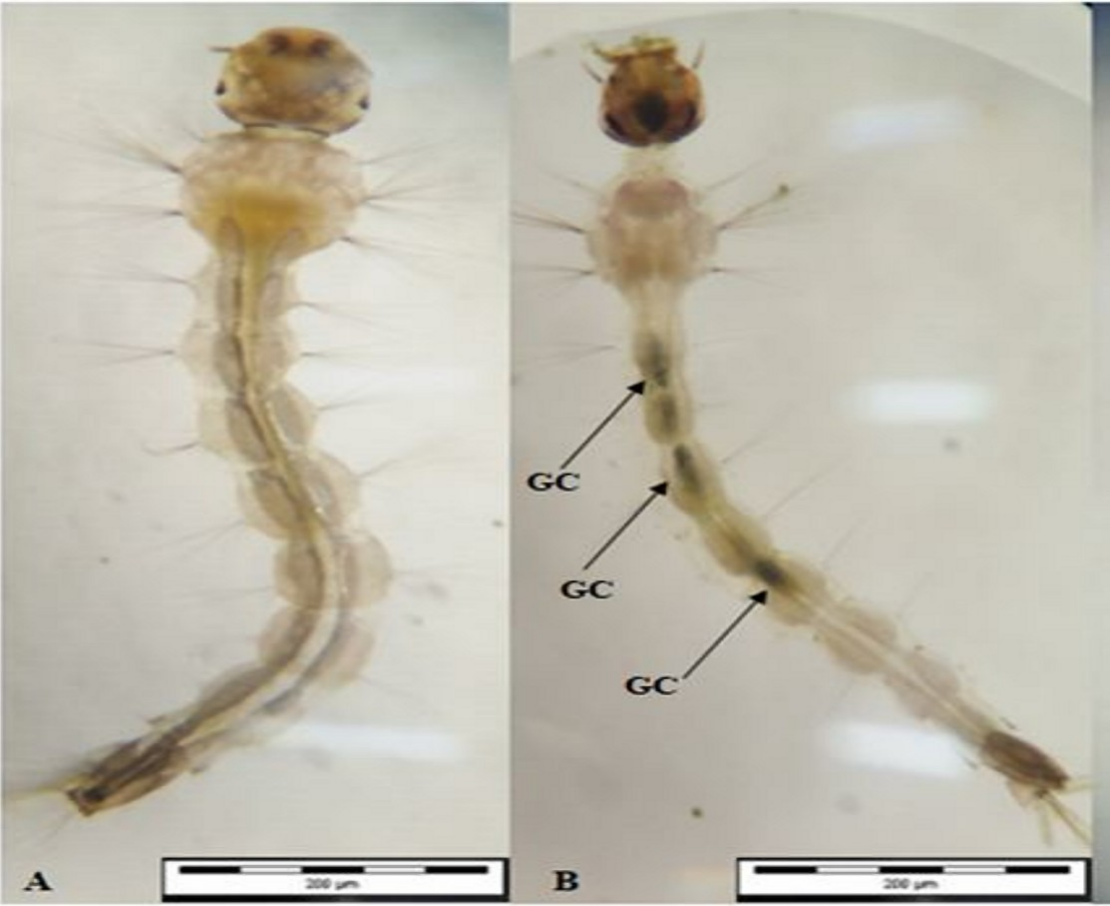
Morphological midgut content induced by *Azolla pinnata* plant extract from maceration extraction method in larvae of *Ae*. *Aegypti*. (A) Control test for midgut content view in early 4^th^ instar larvae of *Ae*. *Aegypti* (B) *A*. *pinnata* crude extract for midgut content view in larvae of *Ae*. *Aegypti* Note: Arrows indicating the plant extracts (greenish color), GC: gut content (after 24hours).

**Fig 9:**
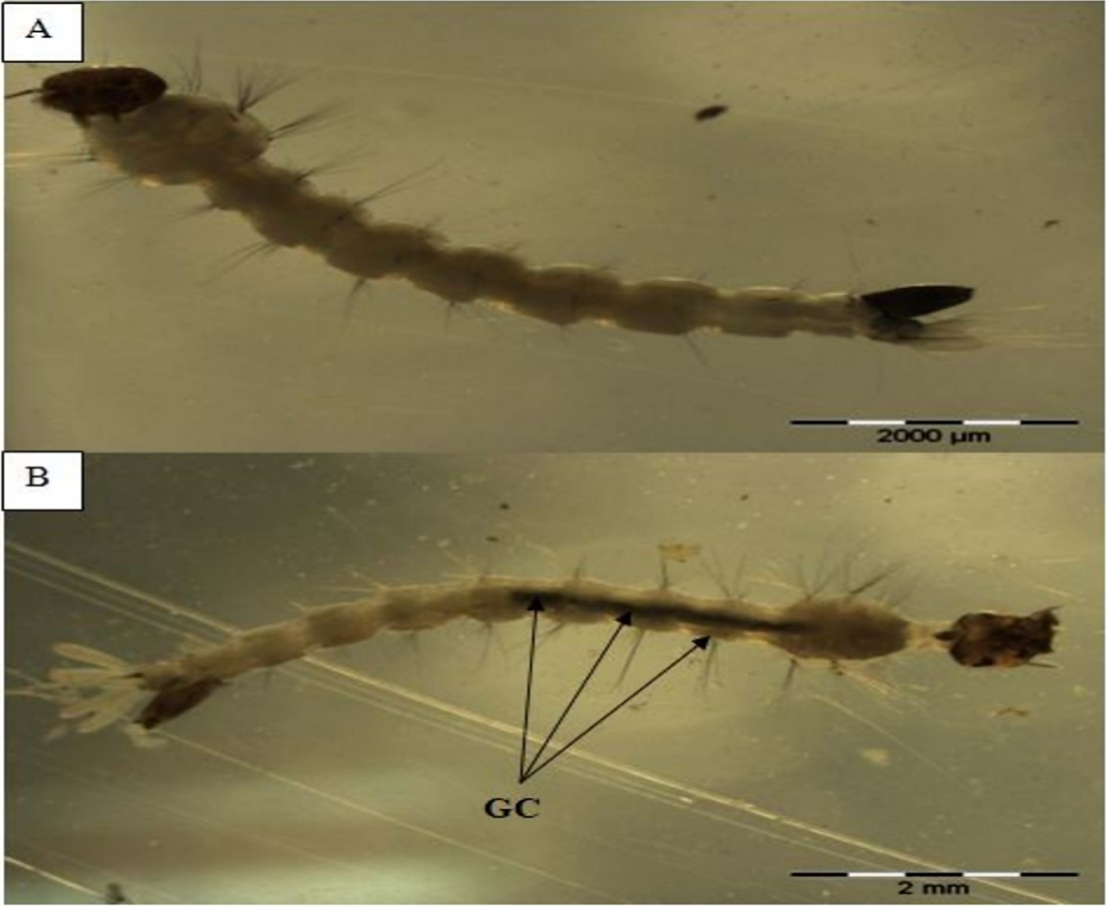
Morphological midgut content induced by *Azolla pinnata* plant extract from maceration and soxhlet extraction method in larvae of *Ae*. *Albopictus*. (A) Control test for midgut content view in early 4^th^ instar larvae of *Ae*. *Albopictus* (B) *A*. *pinnata* crude extract for midgut content view in larvae of *Ae*. *Albopictus* Note: Arrows indicating the plant extracts (greenish color), GC: gut content (after 24hours).

The morphological view shown in Figs 7B, 8B and 9B indicates the presence of *A*. *pinnata* plant extracts in the midgut content by the greenish colour of extracts in comparison with the control test (Figs 7A, 8A and 9A). The greenish colour is due to the extracted plant’s chlorophyll colour.

## Discussion

The findings of this study have shown that the phytochemical compounds extracted from *A*. *pinnata* crude extract could be an innovative application for the conception of a bio-insecticidal product that could be used as an alternative to synthetic chemical insecticides. Additionally, *A*. *pinnata* plant crude extracts could be more effective than a single active compound due to the synergism of its active ingredients, which may be effective in managing the resistant population of mosquitoes. In this study, we found that neophytadiene, 3,7,11,15-tetramethyl-2-hexadecen-1-ol, benzenepropanoic acid, 3,5-bis(1,1-dimethylethyl)-4, 2,4,4,6,6,8,8-heptamethyl-1-nonene, methacrylic acid, and heptadecyl ester compounds from both extraction methods of *A*. *pinnata* plant crude extracts. As stated in Tables 1 and 2, the uses of these compounds are for insecticidal, pesticidal, anti-parasitic, nematicide, antimicrobial and antioxidant activities [31,41,45,46,54–56].

Moreover, the Soxhlet extraction method of the *A*. *pinnata* plant has a higher percentage of diethyl phthalate (20.449%), Bis(2-ethylhexyl) methylphosphonate (17.96%), sulfurous acid, cyclohexylmethyl pentadecyl ester (17.038%), and compounds with a total of all three compounds at 55%. Diethyl phthalate has often been studied for its larvicidal and repellent properties against mosquitoes [56]. Meanwhile, Bis(2-ethylhexyl) methylphosphonate and sulfurous acid, cyclohexylmethyl pentadecyl ester were used for pesticidal activity [50,57,58]. Next, the maceration extraction method has a higher percentage of neophytadiene (27.852%), hexadecanoate <methyl> (14.182%) and phytol (10.043%), with a total of 52% of all three compounds. Neophytadiene, hexadecanoate and phytol were used in many studies for larvicidal activity [59–61].

The results showed that the methanol solvent using the Soxhlet extraction method showed a lower lethal concentration value compared with the maceration method of *A*. *pinnata* crude extracts against the larvae of *Ae*. *aegypti*, and both extraction techniques were mostly equivalent for *Ae*. *albopictus*. Our results were similar to a comparison study between Soxhlet and maceration plant extraction methods by Atanasova et al. [20]. The acetone extract from the Soxhlet extraction method of the *Ipomoea cairica* plant impose better larvicidal properties against *Cx*. *quinquefasciatus* larvae compared to that of extracted by the maceration method. Additionally, according to Navarro-Silva et al. [62], the Soxhlet method was more effective for extracting larvicidal chemical components from the *Azadirachta indica* and *Artemisia annua* plants compared to the reflux and hot extraction methods. The main advantage of using the Soxhlet plant extraction method is due to the disarticulation of the shift equilibrium by repetitively bringing fresh solvent into contact with the plant powder, thus maintaining a relatively high extraction temperature from the distillation flask [21].

Moreover, according to the results, we should not only focus on the disadvantages of the maceration extraction method, because some compounds are unstable in high temperature extraction techniques, and vice versa for some compounds that may not be efficiently extracted at room temperature. The advantage of the maceration method is that it is an easy and simple method that is relatively cheap, because it does not require advance tools [20]. We have conducted two extraction techniques in our study to determine the efficacies of the compounds extracted as to date, because there are no other similar studies that have been conducted on *A*. *pinnata* crude extracts compounds to determine the larvicidal effects.

In addition to that, the residual factors of using *A*. *pinnata* crude extract compounds in water are considered to be harmless, and they promote the growth of plants. According to Bindhu et al., [63], the extraction of the fern *Azolla* sp showed bio-fertilizer ability when soaked in its extracts for the *Pisum sativum* plant. In another study, *Azolla* sp was also used for a water purification system and for the remediation of pesticide-contaminated soils [64]. Additionally, the *Azolla pinnata* extracts did not possess any toxic effects on the fish. Many studies on guppy fish, *Poecilia reticulata*, have shown the abilities of this fish as the biological control of *Aedes* larvae by its larval-eating potential [65, 66]. According a study by Pereira and Olivaira, [67], *Poecilia reticulata* has significant potential effects to predate on *Aedes aegypti* larvae, and this eliminates the breeding ground of larvae. Thus, without the *A*. *pinnata* toxic effects on *Poecilia reticulata*, we could integrate the application of *A*. *pinnata* and fish to control *Aedes* larvae.

Additionally, in aquaculture aspects, the extracts of *A*. *pinnata* were widely used as fish feeds, as it promotes growth of fingerlings and adults [68, 69]. Toxic materials are present in all plant materials; however, the effects of toxic material on the environment differs according to the plant species [70]. As an example, the *Olea europea* leaf contains maslinic acid, which has lower toxic effects on the environment compared to that of chemical insecticides [70]. Hence, depending on the plant species, natural insecticides from plants may possess lesser effects on the environment.

The crude extract and compounds of *A*. *pinnata*, as described here, may lead to the development of natural mosquitocidal products to replace synthetic chemical insecticides. Furthermore, as stated by Cruz-Estrada et al. [71], the application of natural plant-based products by individuals and communities can enhance their ecosystem from chemical pollutants and would provide better vector diseases control programmes. Additionally, the photomicrography view of Figs 5B, 6B and 7B were indicative of the presence of *A*. *pinnata* plant extracts (green colour) in the gut of the larvae after 24 hours of incubation. The gut content of greenish colour intensity compared with that of the control test, Figs 5A, 6A and 7A, showed a similar finding with [72], whereby the effects of *Moringa oleifera* lectin could be seen clearly on the photomicrography of *Aedes* larvae from its gut content. Hence, the application of *A*. *pinnata* crude plant extracts clearly provide an evidential overview of its ingestion mechanisms in larvae. The extracts of *A*. *pinnata* can be seen in the guts of larvae; thus, the possible commercialization of this extract could be based on liquid techniques. Example of similar possible applications, such as the “Dalmatian Powder”, were dissolved in liquid for its application [73]. However, in this current study, we stored the crude extract at -4°C before its application under room temperature. Similarly, Ahbi et al.,[13] prevented the degradation of crude extracts by storing it at -4°C before its application.

The larvicidal application of *A*. *pinnata* plant extracts showed its residual activity until 48 hours in water. Meanwhile, Ullah et al., [74] have stated that 5 different plant species pose residual activity against *Culex quinquefasciatus* until 72 hours of its application. Nevertheless, a field application study of *Azadirachta indica* neem leaf extracts in central Nigeria against mosquito larvae showed residual activity until 72 hours [75]. Elsewhere, a study on a natural herbal extract in comparison with synthetic pesticides to control aphids on cabbage suggested that the residual activity of synthetic pesticides was much longer lasting and active compared to natural herbs [76]. Therefore, it can be concluded that plant-based insecticides may possess lesser residual activity compared to that of synthetic chemicals. Therefore, further studies are needed to determine the residual effect of *Azolla pinnata* extracts. Finally, although *A*. *pinnata* plant extracts could be used as biolarvicides, future testing would have to be conducted to validate its long-term effects on human health and other organisms in the environment.

## Conclusion

In conclusion, the findings of this study have shown the colossal effectiveness of *A*. *pinnata* extracts by two extraction methods against one of the major mosquito species in the early 4^th^ instar larvae stages. Moreover, our findings showed that the crude extract of *A*. *pinnata* bioactive molecules are effective and may be developed as bio-larvicides for *Aedes* mosquito vector control. Finally, this study suggests that future research work can be conducted on the field applications of *A*. *pinnata* extracts to test for its long-term effects on other non-target organisms, including on human health.

## Supporting information

S1 Table This is the Maceration extraction compounds from NIST 08 Library search. (PDF)

S2 Table This is the Soxhlet extraction compounds from NIST 08 library search. (PDF)

S3 Table This is the SPSS data set results for 24 hours maceration (*Ae*. *aegypti)*. (SAV)

S4 Table This is the SPSS data set results for 24 hours soxhlet *(Ae*. *aegypti)*. (SAV)

S5 Table This is the SPSS data set results for 24 hours Probit for maceration (*Ae*. *albopictus*). (SAV)

S6 Table This is the SPSS data set results for 24 hours Probit for soxhlet (*Ae*. *albopictus*). (SAV)

S7 Table This is the SPSS data set results for 24 hours for soxhlet compare maceration (*Ae*. *aegypti*). (SAV)

S8 Table This is the SPSS data set results for 24 hours for soxhlet compare maceration (*Ae*. *albopictus*). (SAV)
