# Supplementary material for: Chemical composition and larvicidal activities of Azolla pinnata extracts against Aedes (Diptera:Culicidae)
Source: PLoS One. 2018 Nov 6;13(11):e0206982. doi: 10.1371/journal.pone.0206982 (PMC6219802; doi:10.1371/journal.pone.0206982)

Sample Information

LIBRARY SEARCH
ID : B
Sample Name : MAC_2000
Date :12/3/2018

Library

<< Target >>
Line#:1 R.Time:3.500(Scan#:1) MassPeaks:441
RawMode:Single 3.500(1) BasePeak:43.95(25562)
BG Mode:None Group 1 - Event 1

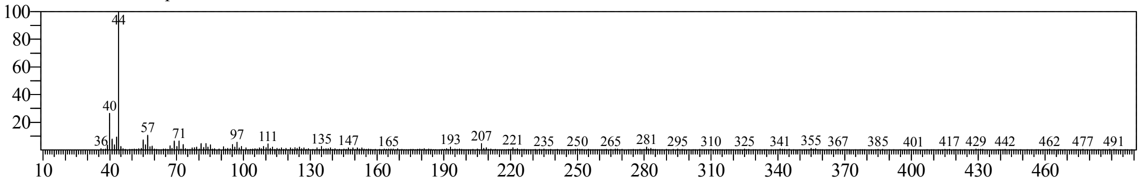

Hit#:1 Entry:26089 Library:NIST08.LIB
SI:77 Formula:C11H25N CAS:13205-56-6 MolWeight:171 RetIndex:1294
CompName:1-Methyldecylamine $$ 2-Undecylamine $$ 2-Undecanamine $$ 2-Aminoundecane $$

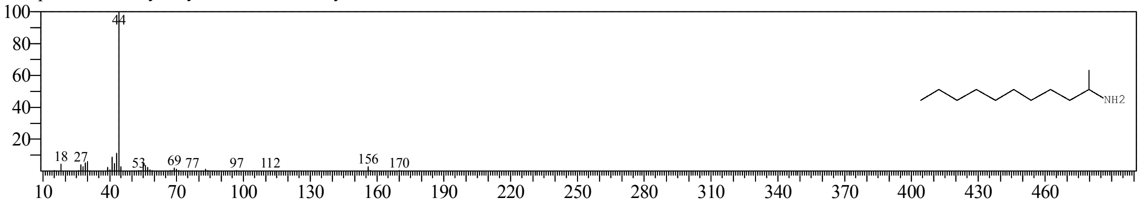

Hit#:2 Entry:1830 Library:NIST08.LIB
SI:76 Formula:C6H13N CAS:5831-72-1 MolWeight:99 RetIndex:806
CompName:Methylpent-4-enylamine $$ N-Methyl-N-(4-pentenyl)amine # $$

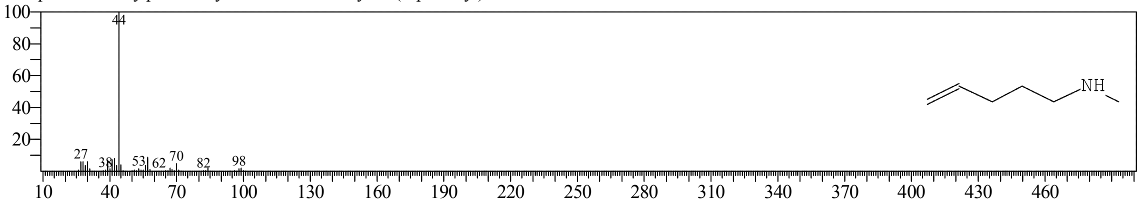

Hit#:3 Entry:43663 Library:NIST08.LIB
SI:76 Formula:C9H18N2O3 CAS:19079-66-4 MolWeight:202 RetIndex:1787
CompName:dl-Alanyl-dl-norleucine $$ N-(2-Aminopropanoyl)norleucine # $$

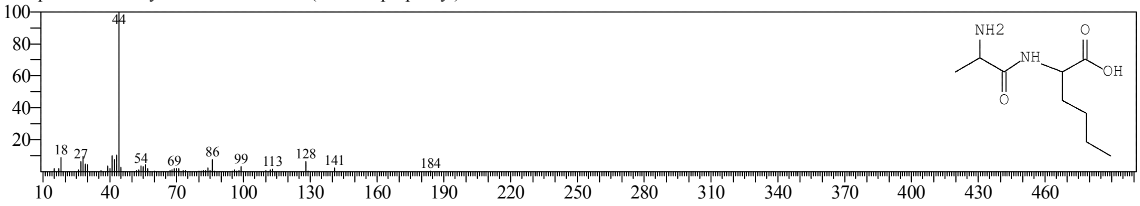

Hit#:4 Entry:13405 Library:NIST08.LIB
SI:76 Formula:C5H10N2O3 CAS:1188-01-8 MolWeight:146 RetIndex:1454
CompName:N-dl-Alanylglycine $$ Glycine, N-DL-alanyl- $$ [(2-Aminopropanoyl)amino]acetic acid # $$

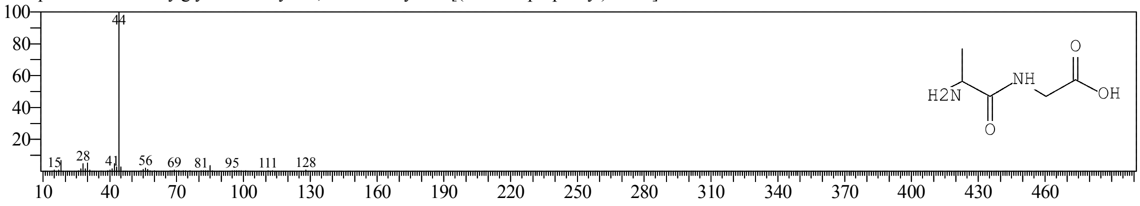

Hit#:5 Entry:99894 Library:NIST08.LIB
SI:76 Formula:C19H41N CAS:31604-55-4 MolWeight:283 RetIndex:2089
CompName:2-Aminononadecane $$ 1-Methyloctadecylamine # $$

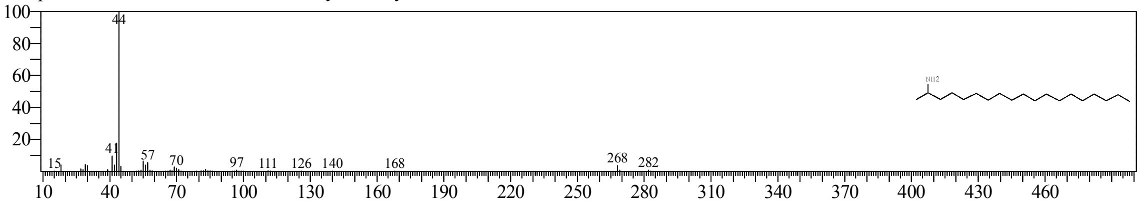

<< Target >>
Line#:2   R.Time:3.500(Scan#:1) MassPeaks:441
RawMode:Single 3.500(1) BasePeak:43.95(25562)
BG Mode:None Group 1 - Event 1

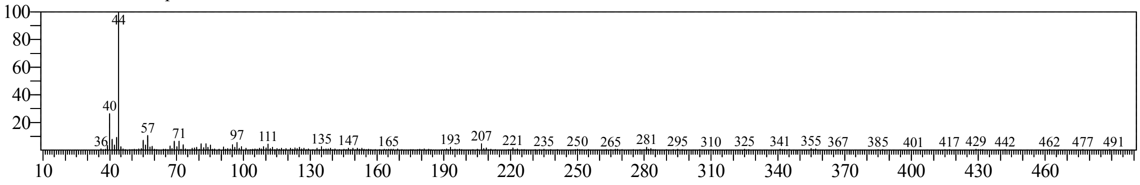

Hit#:1 Entry:26089 Library:NIST08.LIB
SI:77 Formula:C11H25N CAS:13205-56-6 MolWeight:171 RetIndex:1294
CompName:1-Methyldecylamine $$ 2-Undecylamine $$ 2-Undecanamine $$ 2-Aminoundecane $$

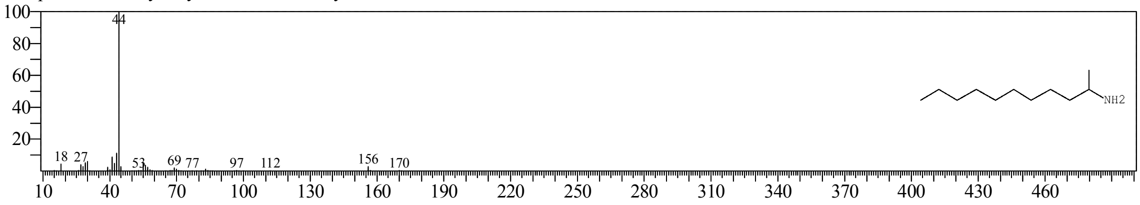

Hit#:2 Entry:1830 Library:NIST08.LIB
SI:76 Formula:C6H13N CAS:5831-72-1 MolWeight:99 RetIndex:806
CompName:Methylpent-4-enylamine $$ N-Methyl-N-(4-pentenyl)amine # $$

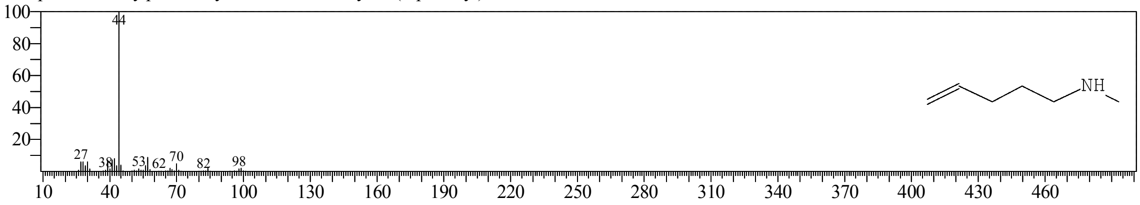

Hit#:3 Entry:43663 Library:NIST08.LIB
SI:76 Formula:C9H18N2O3 CAS:19079-66-4 MolWeight:202 RetIndex:1787
CompName:dl-Alanyl-dl-norleucine $$ N-(2-Aminopropanoyl)norleucine # $$

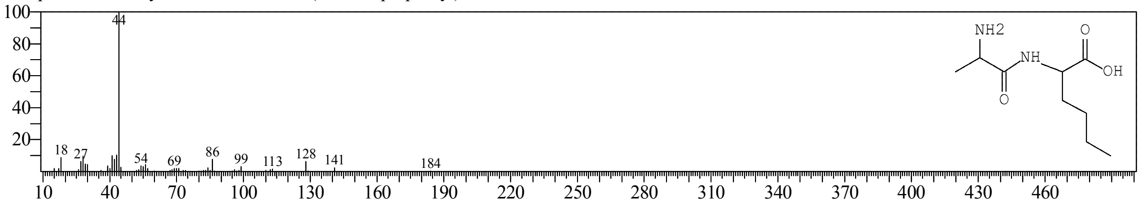

Hit#:4 Entry:13405 Library:NIST08.LIB
SI:76 Formula:C5H10N2O3 CAS:1188-01-8 MolWeight:146 RetIndex:1454
CompName:N-dl-Alanylglycine $$ Glycine, N-DL-alanyl- $$ [(2-Aminopropanoyl)amino]acetic acid # $$

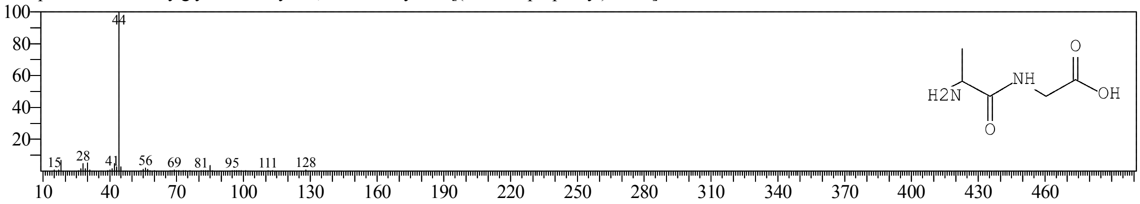

Hit#:5 Entry:99894 Library:NIST08.LIB
SI:76 Formula:C19H41N CAS:31604-55-4 MolWeight:283 RetIndex:2089
CompName:2-Aminononadecane $$ 1-Methyloctadecylamine # $$

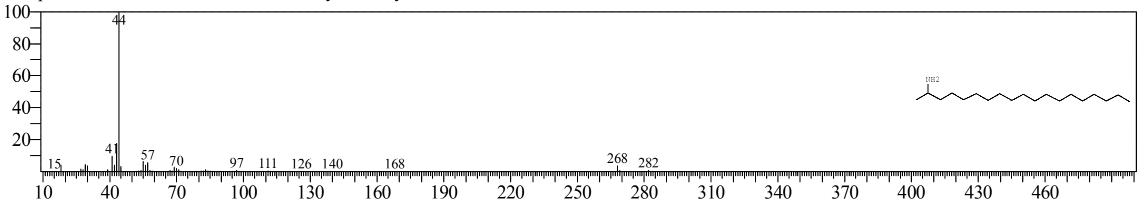

<< Target >>
Line#:3   R.Time:26.005(Scan#:4502) MassPeaks:252
RawMode:Single 26.005(4502) BasePeak:39.95(8786)
BG Mode:25.855(4472) Group 1 - Event 1

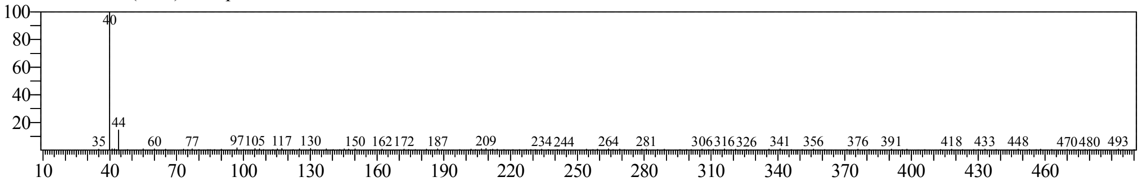

Hit#:1 Entry:30 Library:NIST08.LIB
SI:79 Formula:C3H4 CAS:74-99-7 MolWeight:40 RetIndex:317
CompName:Propyne $$ Methylacetylene $$ 1-Propyne $$ Allylene $$ Propine $$ CH3C.$.CH $$ Acetylene, methyl- $$

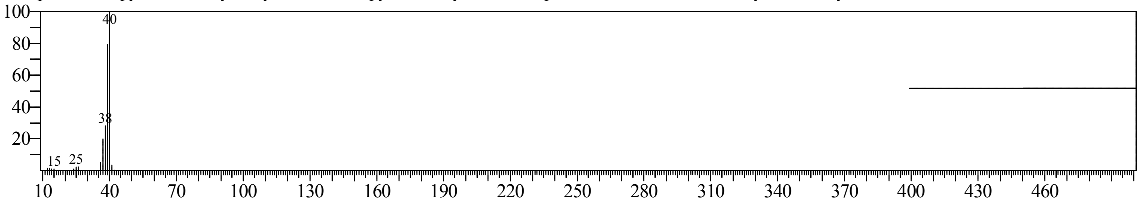

Hit#:2 Entry:29 Library:NIST08.LIB
SI:79 Formula:C3H4 CAS:463-49-0 MolWeight:40 RetIndex:0
CompName:Allene $$ Propadiene $$ 1,2-Propadiene $$ sym-Allylene $$ Dimethylenemethane $$ CH2=C=CH2 $$ Propadiene, inhibited $$ UN 2200 $$ P

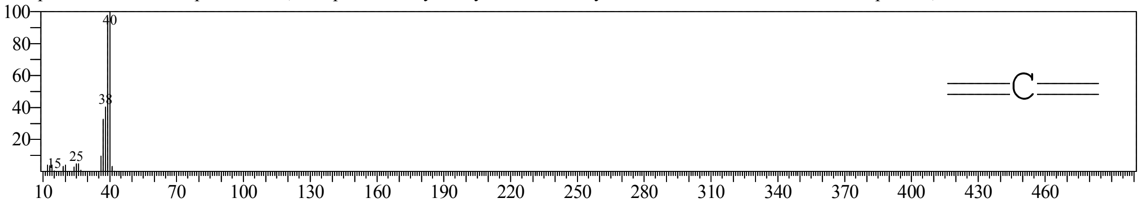

Hit#:3 Entry:27 Library:NIST08.LIB
SI:79 Formula:Ar CAS:7440-37-1 MolWeight:40 RetIndex:0
CompName:Argon $$ Ar $$ UN 1006 $$ UN 1951 $$ argon atom $$

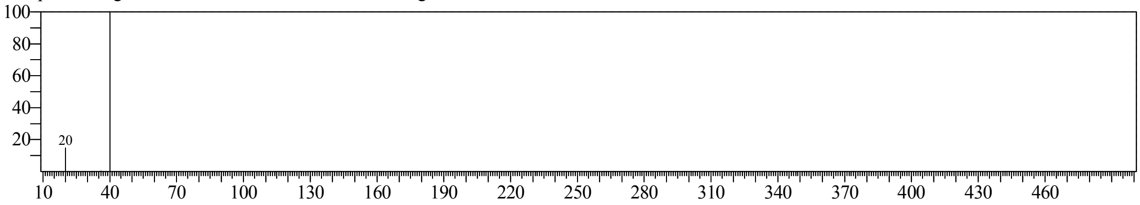

Hit#:4 Entry:28 Library:NIST08.LIB
SI:77 Formula:C3H4 CAS:2781-85-3 MolWeight:40 RetIndex:342
CompName:Cyclopropene $$ 1-Cyclopropene # $$

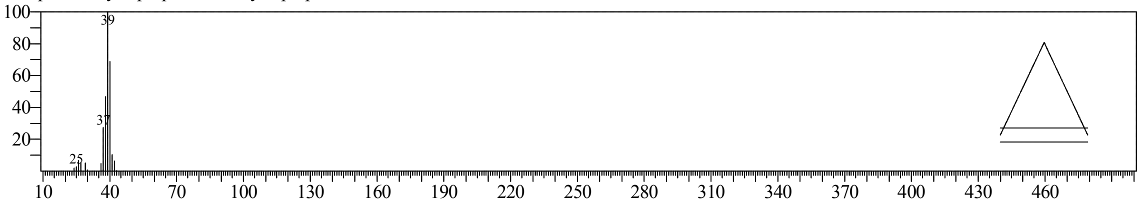

Hit#:5 Entry:36 Library:NIST08.LIB
SI:73 Formula:CH3BO CAS:13205-44-2 MolWeight:42 RetIndex:0
CompName:Borane carbonyl $$ Boron, carbonyltrihydro-, (T-4)- $$ Borane, compd. with carbon monoxide (1:1) $$ Borane-carbon monoxide (1:1) $$ Bori

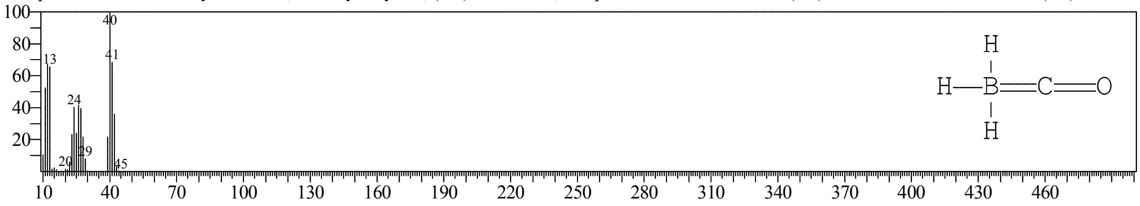

<< Target >>
Line#:4 R.Time:26.820(Scan#:4665) MassPeaks:237
RawMode:Single 26.820(4665) BasePeak:57.05(1005)
BG Mode:26.765(4654) Group 1 - Event 1

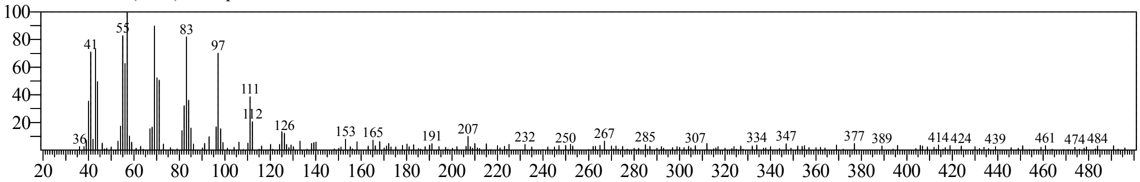

Hit#:1 Entry:20509 Library:NIST08s.LIB
SI:90 Formula:C17H34 CAS:6765-39-5 MolWeight:238 RetIndex:1701
CompName:1-Heptadecene $$ Hexahydroaplotaxene $$

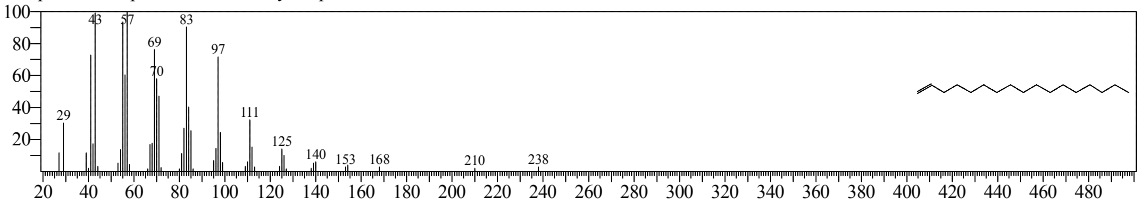

Hit#:2 Entry:97784 Library:NIST08.LIB
SI:89 Formula:C20H40 CAS:74685-29-3 MolWeight:280 RetIndex:2017
CompName:9-Eicosene, (E)- $$ (9E)-9-Icosene # $$

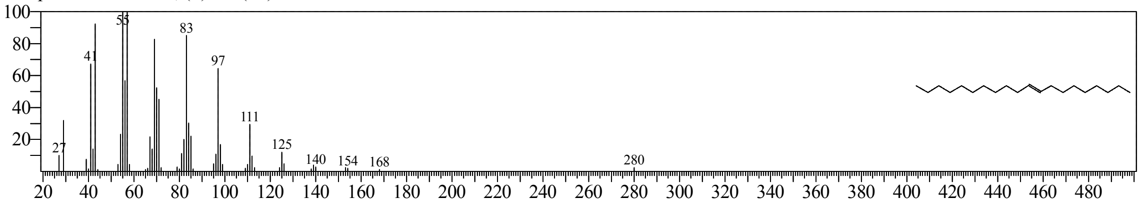

Hit#:3 Entry:128430 Library:NIST08.LIB
SI:89 Formula:C17H31F3O2 CAS:0-00-0 MolWeight:324 RetIndex:1713
CompName:Pentadecyl trifluoroacetate

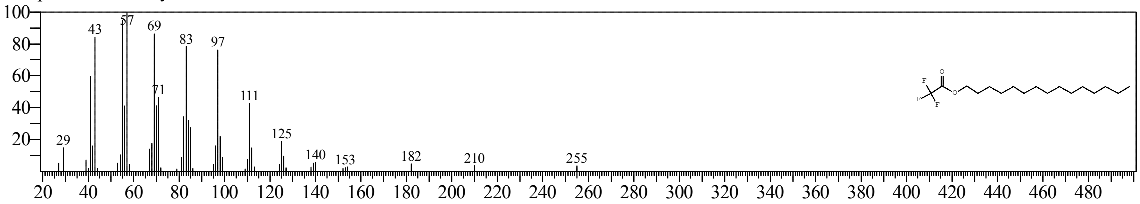

Hit#:4 Entry:17848 Library:NIST08s.LIB
SI:89 Formula:C15H30 CAS:13360-61-7 MolWeight:210 RetIndex:1502
CompName:1-Pentadecene $$ Pentadecene,1- $$ Pentadec-1-ene $$

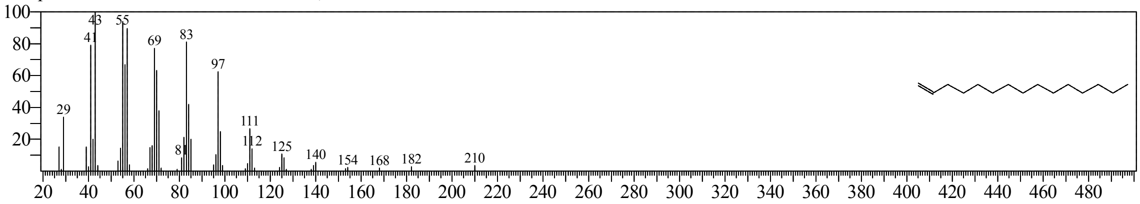

Hit#:5 Entry:97785 Library:NIST08.LIB
SI:89 Formula:C20H40 CAS:74685-33-9 MolWeight:280 RetIndex:2017
CompName:3-Eicosene, (E)- $$ (3E)-3-Icosene # $$

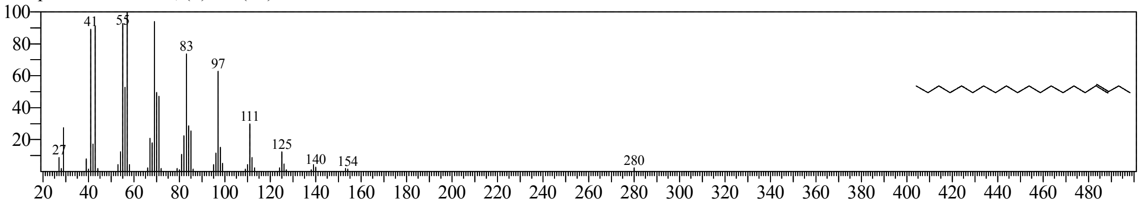

<< Target >>
Line#:5 R.Time:34.435(Scan#:6188) MassPeaks:256
RawMode:Single 34.435(6188) BasePeak:83.10(1580)
BG Mode:34.345(6170) Group 1 - Event 1

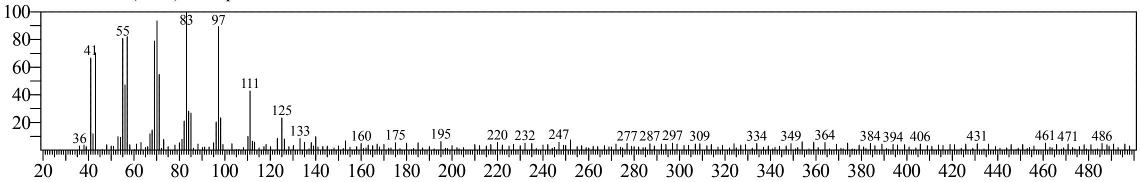

Hit#:1 Entry:22496 Library:NIST08s.LIB
SI:89 Formula:C19H38 CAS:18435-45-5 MolWeight:266 RetIndex:1900
CompName:1-Nonadecene

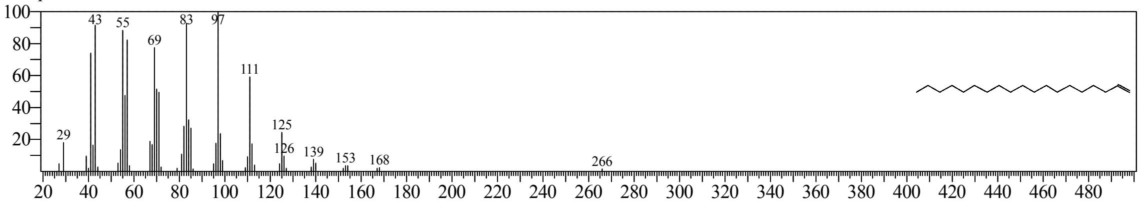

Hit#:2 Entry:100901 Library:NIST08.LIB
SI:89 Formula:C19H40O CAS:1454-84-8 MolWeight:284 RetIndex:2153
CompName:n-Nonadecanol-1 $$ 1-Nonadecanol $$ Nonadecanol $$ Nonadecan-1-ol $$ Nonadecyl alcohol $$

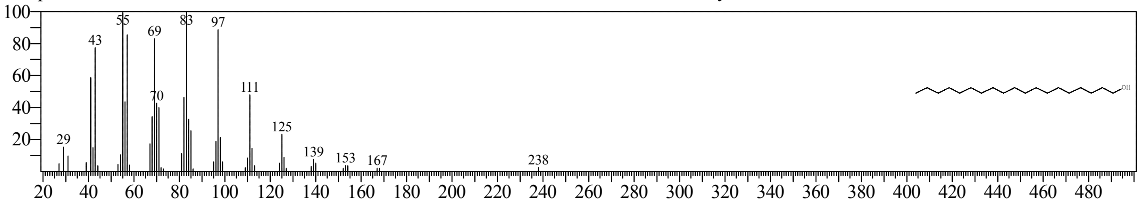

Hit#:3 Entry:61696 Library:NIST08.LIB
SI:88 Formula:C15H32O CAS:629-76-5 MolWeight:228 RetIndex:1755
CompName:n-Pentadecanol $$ n-1-Pentadecanol $$ Pentadecanol $$ Neodol 5 $$ 1-Pentadecanol $$ Pentadecan-1-ol $$ Pentadecyl alcohol $$

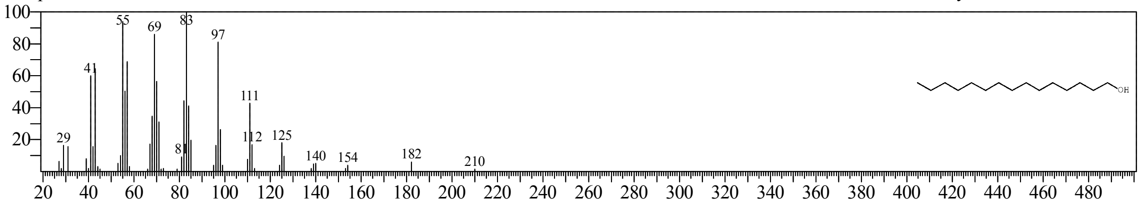

Hit#:4 Entry:153365 Library:NIST08.LIB
SI:88 Formula:C19H36Cl2O2 CAS:0-00-0 MolWeight:366 RetIndex:2423
CompName:Dichloroacetic acid, heptadecyl ester

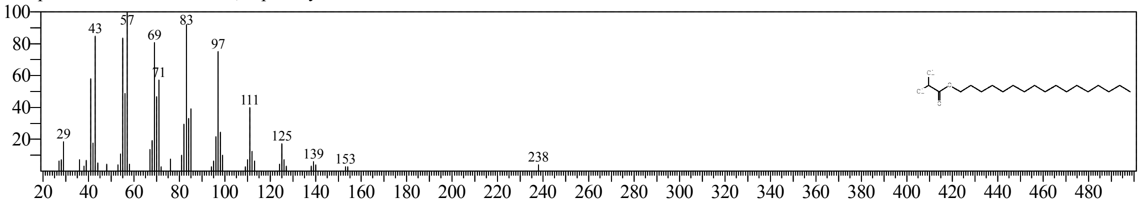

Hit#:5 Entry:25469 Library:NIST08s.LIB
SI:88 Formula:C22H46O CAS:661-19-8 MolWeight:326 RetIndex:2451
CompName:Behenic alcohol $$ 1-Docosanol $$ Docosyl alcohol $$ Docosanol-(1) $$ Cachalot BE-22 $$ Dehydag wax 22 (lanette) $$ Emery 3304 $$ Lo[illegible]

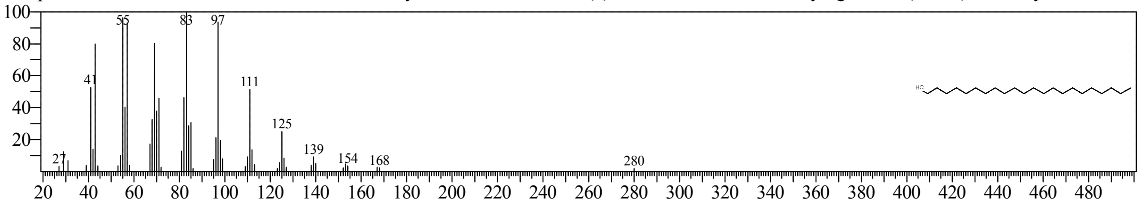

<< Target >>
Line#:6   R.Time:35.925(Scan#:6486) MassPeaks:290
RawMode:Single 35.925(6486) BasePeak:68.00(22227)
BG Mode:35.835(6468) Group 1 - Event 1

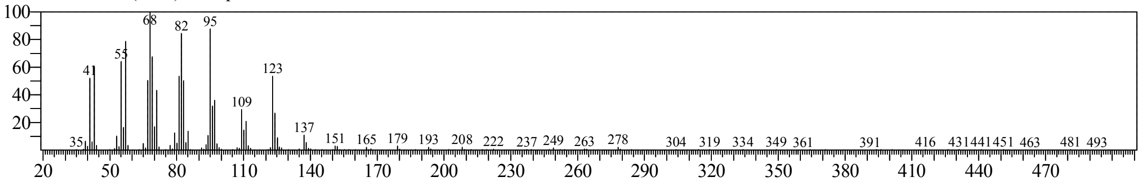

Hit#:1 Entry:908 Library:FFNSC1.3.lib
SI:95 Formula:C20 H38 CAS:504-96-1 MolWeight:278 RetIndex:1836
CompName:Neophytadiene

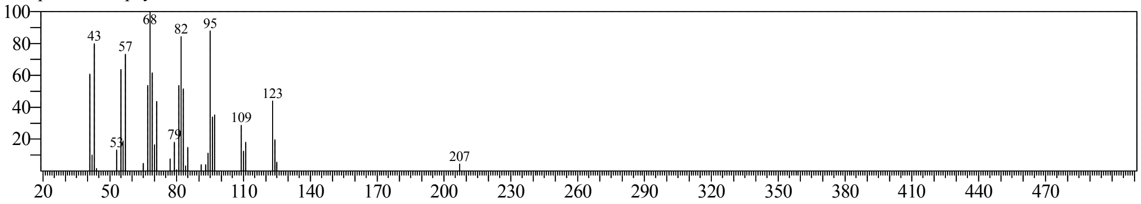

Hit#:2 Entry:109355 Library:NIST08.LIB
SI:90 Formula:C20H40O CAS:102608-53-7 MolWeight:296 RetIndex:2045
CompName:3,7,11,15-Tetramethyl-2-hexadecen-1-ol $$ (2E)-3,7,11,15-Tetramethyl-2-hexadecen-1-ol # $$

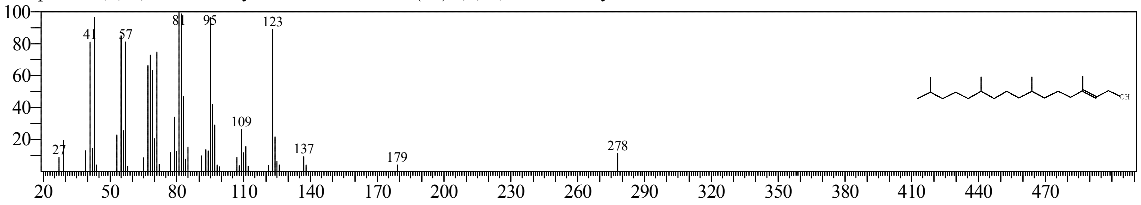

Hit#:3 Entry:60326 Library:NIST08.LIB
SI:88 Formula:C15H30O CAS:2765-11-9 MolWeight:226 RetIndex:1701
CompName:Pentadecanal-

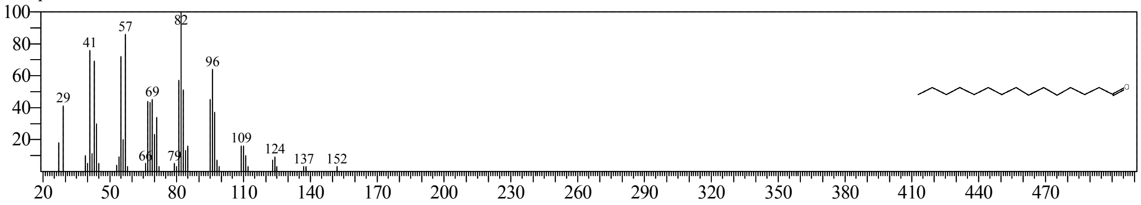

Hit#:4 Entry:89232 Library:NIST08.LIB
SI:88 Formula:C18H36O CAS:143-28-2 MolWeight:268 RetIndex:2061
CompName:Oleyl Alcohol $$ 9-Octadecen-1-ol, (Z)- $$ cis-9-Octadecen-1-ol $$ cis-9-Octadecenyl Alcohol $$ Adol 320 $$ Adol 85 $$ Atalco O $$ Cachal

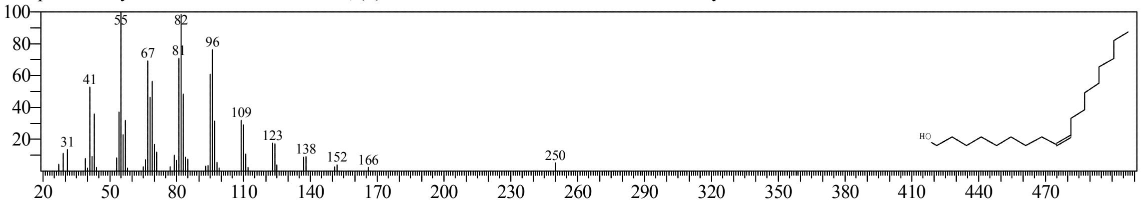

Hit#:5 Entry:1115 Library:FFNSC1.3.lib
SI:88 Formula:C22 H42 O2 CAS:76337-16-1 MolWeight:338 RetIndex:2212
CompName:Phytol acetate

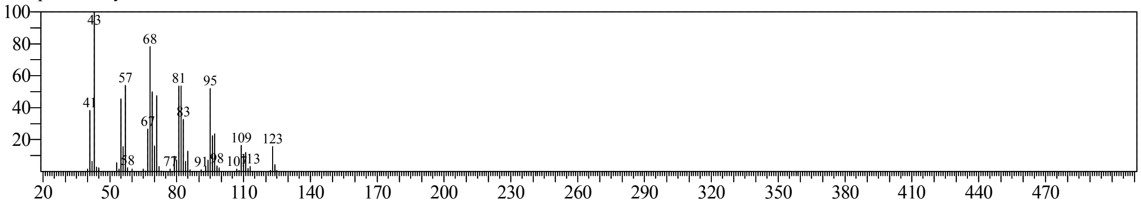

<< Target >>
Line#:7 R.Time:36.810(Scan#:6663) MassPeaks:265
RawMode:Single 36.810(6663) BasePeak:82.10(4546)
BG Mode:36.730(6647) Group 1 - Event 1

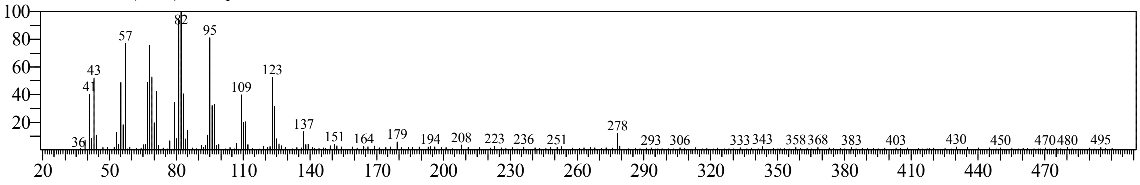

Hit#:1 Entry:109355 Library:NIST08.LIB
SI:92 Formula:C20H40O CAS:102608-53-7 MolWeight:296 RetIndex:2045
CompName:3,7,11,15-Tetramethyl-2-hexadecen-1-ol $$ (2E)-3,7,11,15-Tetramethyl-2-hexadecen-1-ol # $$

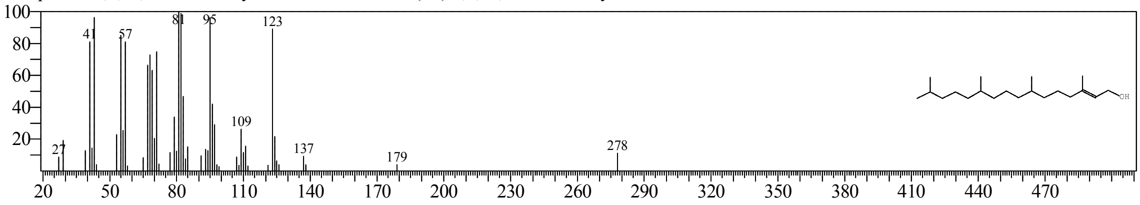

Hit#:2 Entry:908 Library:FFNSC1.3.lib
SI:90 Formula:C20 H38 CAS:504-96-1 MolWeight:278 RetIndex:1836
CompName:Neophytadiene

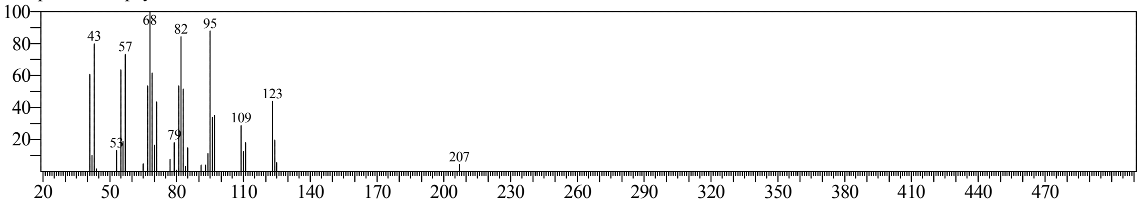

Hit#:3 Entry:96436 Library:NIST08.LIB
SI:87 Formula:C20H38 CAS:61886-66-6 MolWeight:278 RetIndex:2027
CompName:3-Eicosyne $$ 3-Icosyne # $$

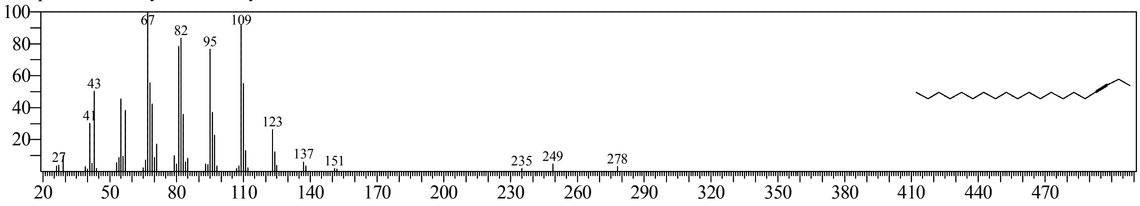

Hit#:4 Entry:86382 Library:NIST08.LIB
SI:87 Formula:C19H36 CAS:35354-38-2 MolWeight:264 RetIndex:1863
CompName:7-Octadecyne, 2-methyl- $$ 2-Methyl-7-octadecyne $$

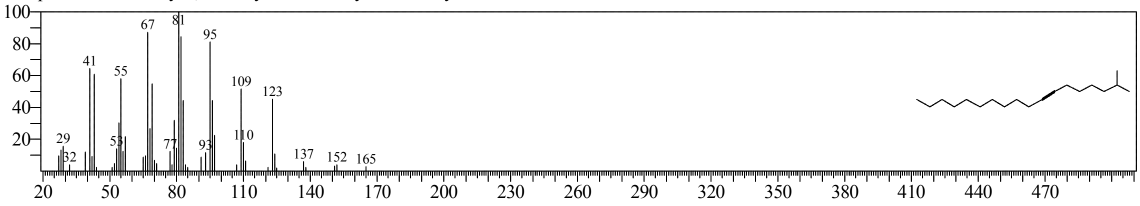

Hit#:5 Entry:96439 Library:NIST08.LIB
SI:87 Formula:C20H38 CAS:71899-38-2 MolWeight:278 RetIndex:2027
CompName:9-Eicosyne $$ 9-Icosyne # $$

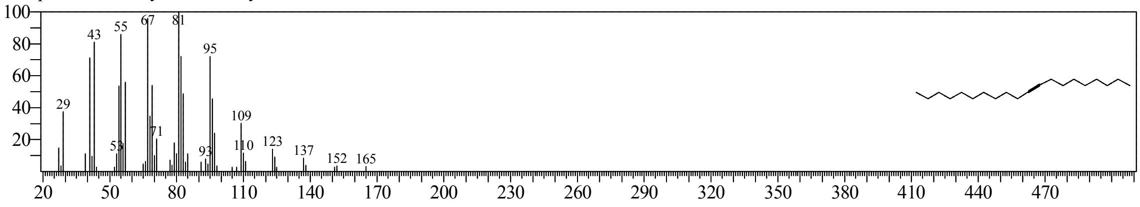

<< Target >>
Line#:8 R.Time:37.450(Scan#:6791) MassPeaks:284
RawMode:Single 37.450(6791) BasePeak:82.10(7010)
BG Mode:37.325(6766) Group 1 - Event 1

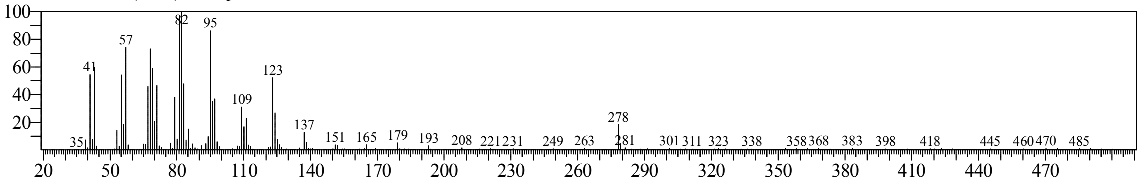

Hit#:1 Entry:109355 Library:NIST08.LIB
SI:92 Formula:C20H40O CAS:102608-53-7 MolWeight:296 RetIndex:2045
CompName:3,7,11,15-Tetramethyl-2-hexadecen-1-ol $$ (2E)-3,7,11,15-Tetramethyl-2-hexadecen-1-ol # $$

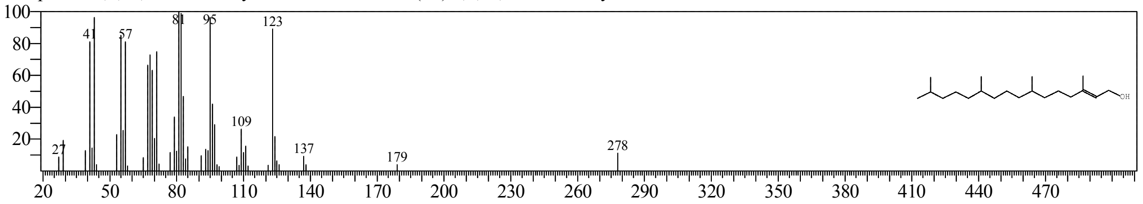

Hit#:2 Entry:908 Library:FFNSC1.3.lib
SI:91 Formula:C20 H38 CAS:504-96-1 MolWeight:278 RetIndex:1836
CompName:Neophytadiene

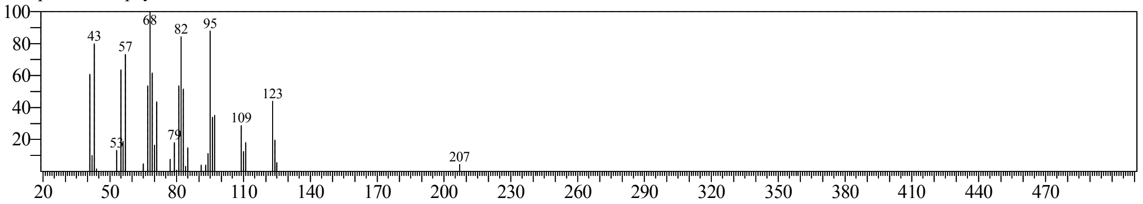

Hit#:3 Entry:86382 Library:NIST08.LIB
SI:87 Formula:C19H36 CAS:35354-38-2 MolWeight:264 RetIndex:1863
CompName:7-Octadecyne, 2-methyl- $$ 2-Methyl-7-octadecyne $$

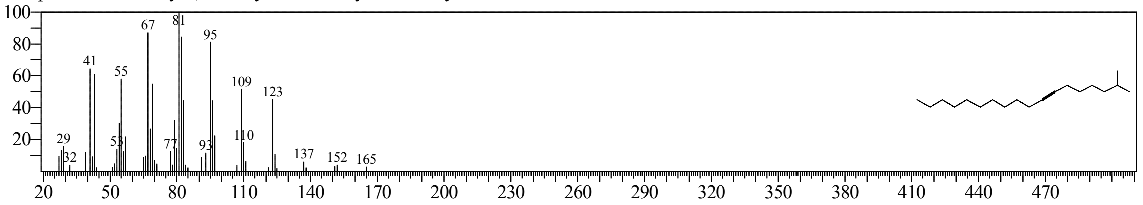

Hit#:4 Entry:89232 Library:NIST08.LIB
SI:86 Formula:C18H36O CAS:143-28-2 MolWeight:268 RetIndex:2061
CompName:Oleyl Alcohol $$ 9-Octadecen-1-ol, (Z)- $$ cis-9-Octadecen-1-ol $$ cis-9-Octadecenyl Alcohol $$ Adol 320 $$ Adol 85 $$ Atalco O $$ Cachal

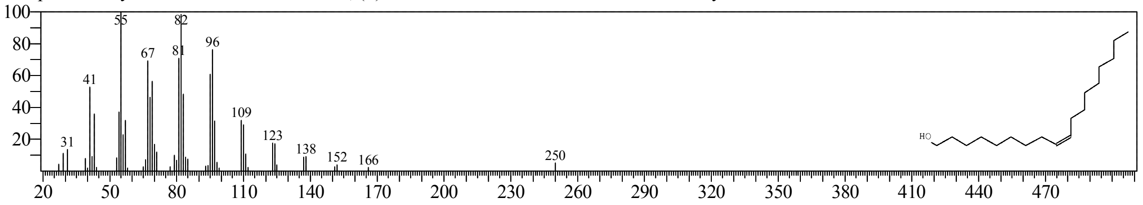

Hit#:5 Entry:96439 Library:NIST08.LIB
SI:86 Formula:C20H38 CAS:71899-38-2 MolWeight:278 RetIndex:2027
CompName:9-Eicosyne $$ 9-Icosyne # $$

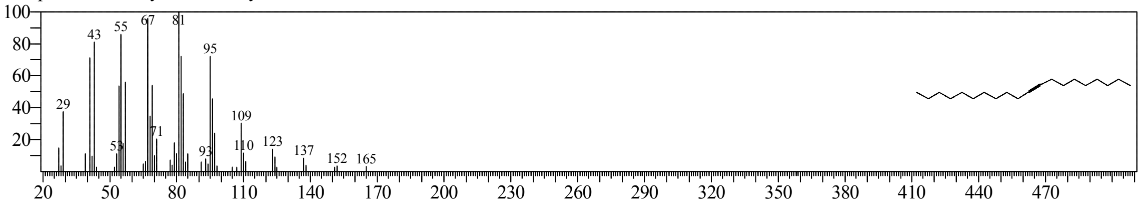

<< Target >>
Line#:9 R.Time:39.260(Scan#:7153) MassPeaks:273
RawMode:Single 39.260(7153) BasePeak:74.00(11311)
BG Mode:39.180(7137) Group 1 - Event 1

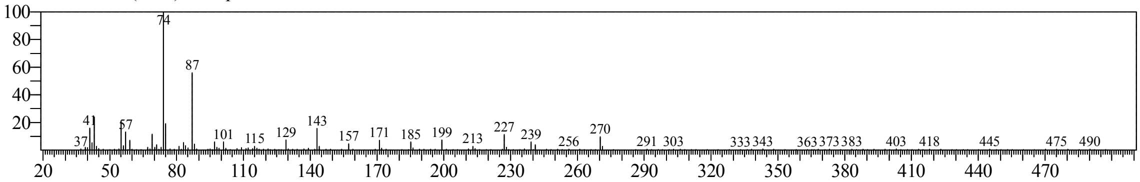

Hit#:1 Entry:587 Library:FFNSC1.3.lib
SI:93 Formula:C17 H34 O2 CAS:112-39-0 MolWeight:270 RetIndex:1925
CompName:Hexadecanoate <methyl->

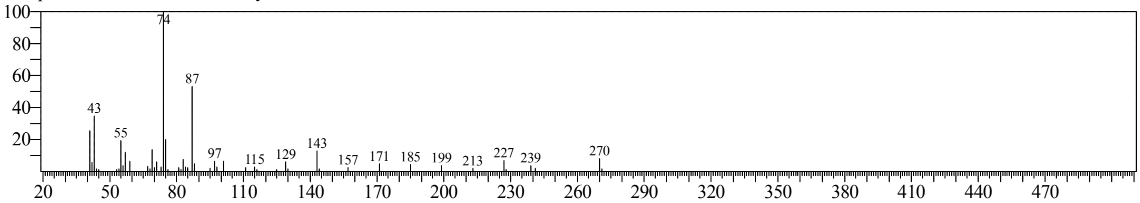

Hit#:2 Entry:90723 Library:NIST08.LIB
SI:89 Formula:C17H34O2 CAS:27147-71-3 MolWeight:270 RetIndex:2003
CompName:Hexadecanoic acid, 2-methyl- $$ 2-Methylhexadecanoic acid # $$

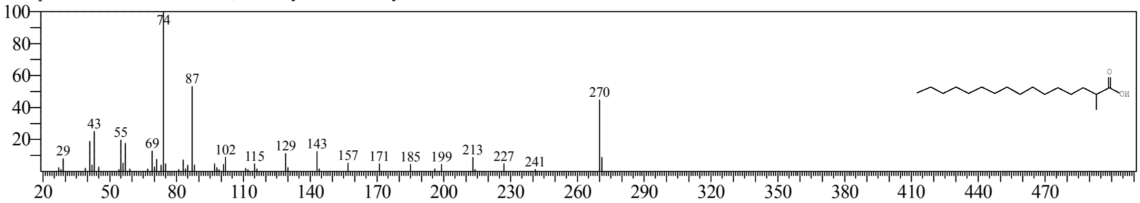

Hit#:3 Entry:80758 Library:NIST08.LIB
SI:89 Formula:C16H32O2 CAS:7132-64-1 MolWeight:256 RetIndex:1779
CompName:Pentadecanoic acid, methyl ester $$ Methyl n-pentadecanoate $$ Methyl pentadecanoate $$ n-Pentadecanoic acid methyl ester $$

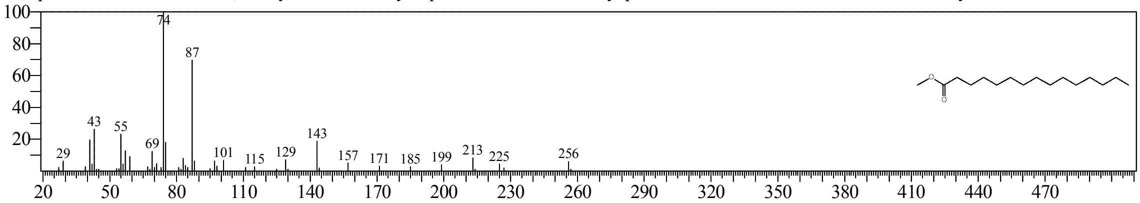

Hit#:4 Entry:90720 Library:NIST08.LIB
SI:89 Formula:C17H34O2 CAS:5129-60-2 MolWeight:270 RetIndex:1814
CompName:Pentadecanoic acid, 14-methyl-, methyl ester $$ Methyl 14-methylpentadecanoate # $$

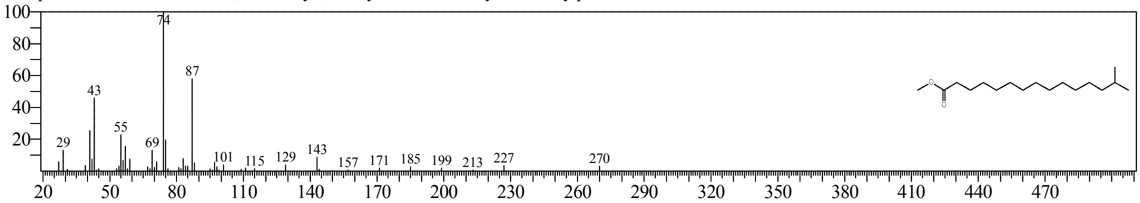

Hit#:5 Entry:61606 Library:NIST08.LIB
SI:89 Formula:C14H28O2 CAS:1731-88-0 MolWeight:228 RetIndex:1580
CompName:Tridecanoic acid, methyl ester $$ Methyl tridecanoate $$ n-Tridecanoic acid methyl ester $$ Methyl ester of tridecanoic acid $$

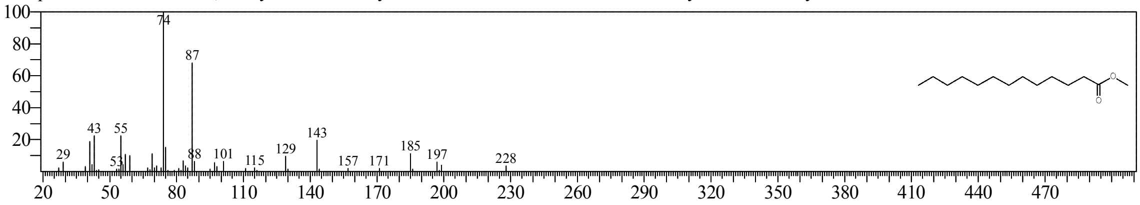

<< Target >>
Line#:10 R.Time:39.450(Scan#:7191) MassPeaks:287
RawMode:Single 39.450(7191) BasePeak:277.15(6051)
BG Mode:39.355(7172) Group 1 - Event 1

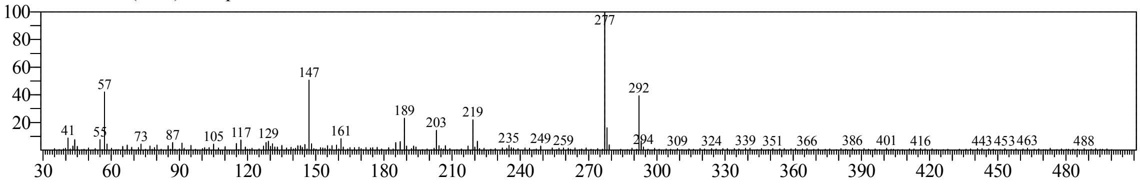

Hit#:1 Entry:106456 Library:NIST08.LIB
SI:84 Formula:C18H28O3 CAS:6386-38-5 MolWeight:292 RetIndex:2134
CompName:Benzenepropanoic acid, 3,5-bis(1,1-dimethylethyl)-4-hydroxy-, methyl ester $$ Methyl 3-(3,5-di-tert-butyl-4-hydroxyphenyl)propionate $$ Metl

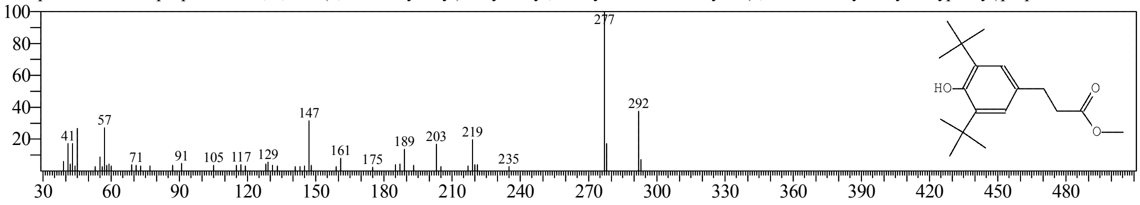

<< Target >>
Line#:11 R.Time:40.600(Scan#:7421) MassPeaks:272
RawMode:Single 40.600(7421) BasePeak:73.00(2658)
BG Mode:40.520(7405) Group 1 - Event 1

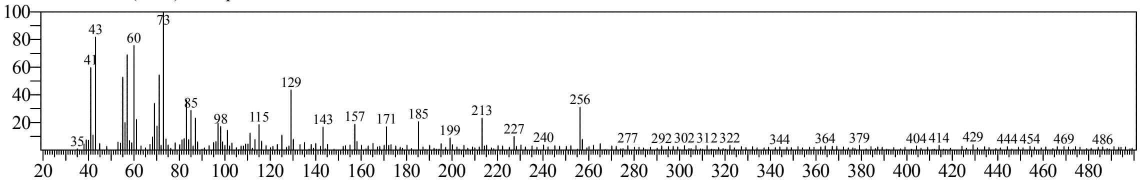

Hit#:1 Entry:626 Library:FFNSC1.3.lib
SI:91 Formula:C16 H32 O2 CAS:57-10-3 MolWeight:256 RetIndex:1977
CompName:Hexadecanoic acid <n->

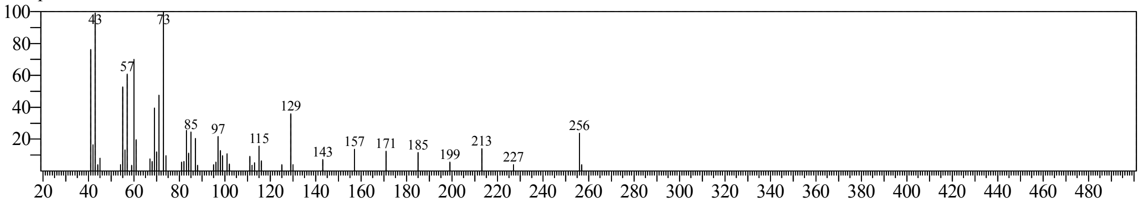

Hit#:2 Entry:189990 Library:NIST08.LIB
SI:91 Formula:C38H68O8 CAS:28474-90-0 MolWeight:652 RetIndex:4765
CompName:l-(+)-Ascorbic acid 2,6-dihexadecanoate

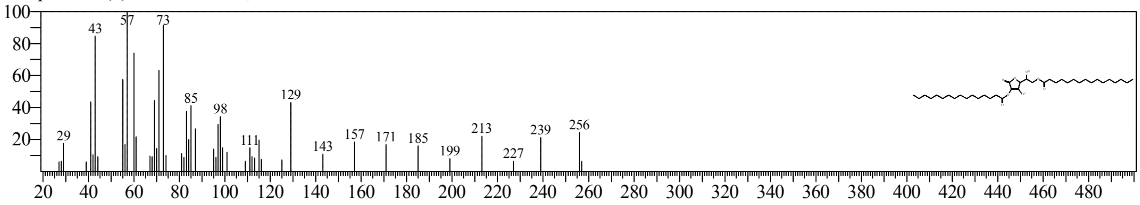

Hit#:3 Entry:20839 Library:NIST08s.LIB
SI:89 Formula:C15H30O2 CAS:1002-84-2 MolWeight:242 RetIndex:1869
CompName:Pentadecanoic acid $$ Pentadecylic acid $$ n-Pentadecanoic acid $$ n-Pentadecylic acid $$

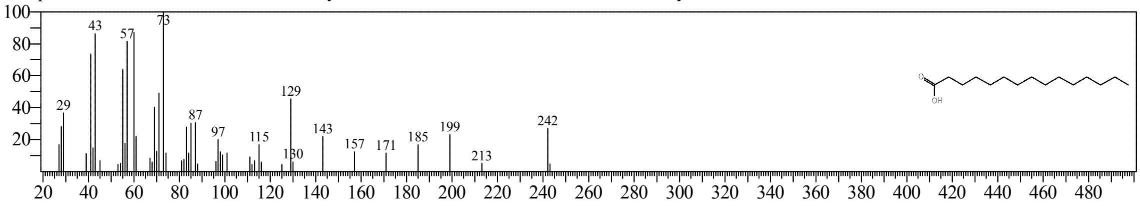

Hit#:4 Entry:61604 Library:NIST08.LIB
SI:87 Formula:C14H28O2 CAS:544-63-8 MolWeight:228 RetIndex:1769
CompName:Tetradecanoic acid $$ Myristic acid $$ n-Tetradecanoic acid $$ n-Tetradecoic acid $$ Neo-Fat 14 $$ Univol U 316S $$ 1-Tridecanecarboxylic a

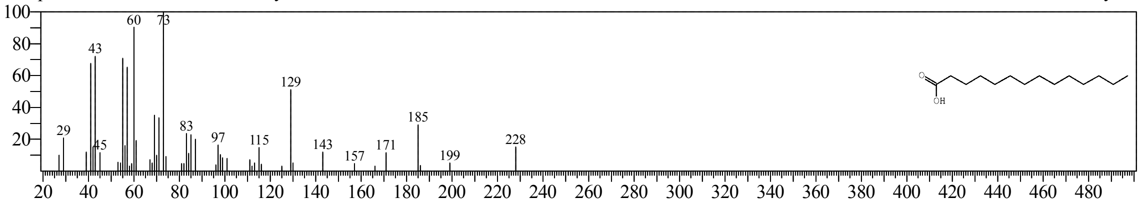

Hit#:5 Entry:100791 Library:NIST08.LIB
SI:86 Formula:C18H36O2 CAS:57-11-4 MolWeight:284 RetIndex:2167
CompName:Octadecanoic acid $$ Stearic acid $$ n-Octadecanoic acid $$ Humko Industrene R $$ Hydrofol Acid 150 $$ Hystrene S-97 $$ Hystrene T-70 $

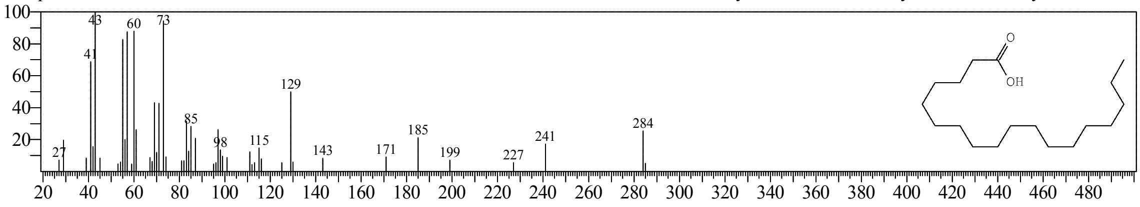

<< Target >>
Line#:12 R.Time:44.960(Scan#:8293) MassPeaks:295
RawMode:Single 44.960(8293) BasePeak:55.05(2650)
BG Mode:44.860(8273) Group 1 - Event 1

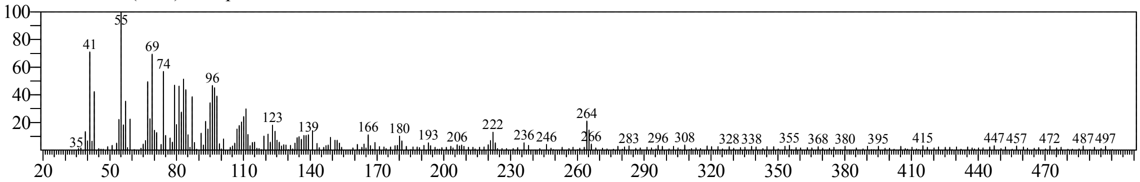

Hit#:1 Entry:24160 Library:NIST08s.LIB
SI:90 Formula:C19H36O2 CAS:1937-62-8 MolWeight:296 RetIndex:2085
CompName:9-Octadecenoic acid, methyl ester, (E)- $$ Elaidic acid, methyl ester $$ Methyl elaidate $$ Methyl trans-9-octadecenoate $$ (E)-9-Octadecenoic

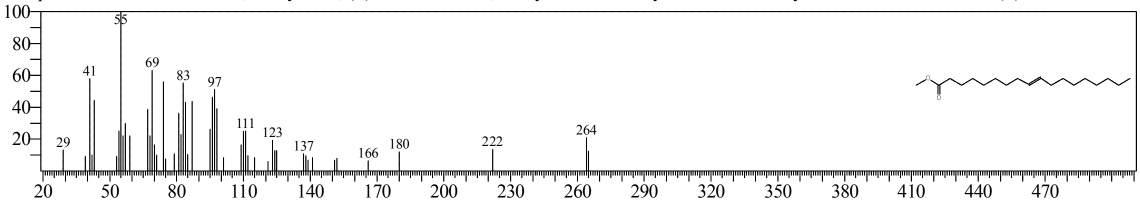

Hit#:2 Entry:109242 Library:NIST08.LIB
SI:88 Formula:C19H36O2 CAS:57396-98-2 MolWeight:296 RetIndex:2085
CompName:7-Octadecenoic acid, methyl ester $$ Methyl (7E)-7-octadecenoate # $$

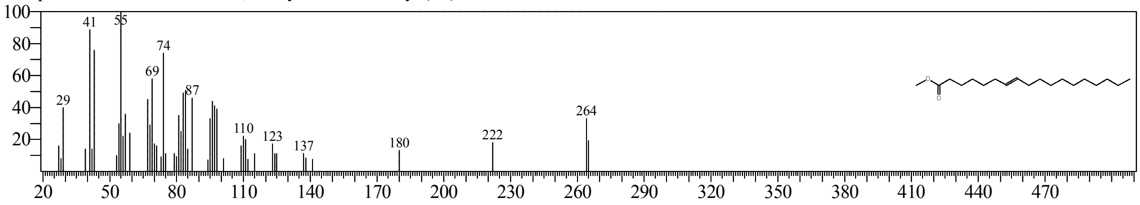

Hit#:3 Entry:109239 Library:NIST08.LIB
SI:88 Formula:C19H36O2 CAS:2345-29-1 MolWeight:296 RetIndex:2085
CompName:8-Octadecenoic acid, methyl ester $$ Methyl (8E)-8-octadecenoate # $$

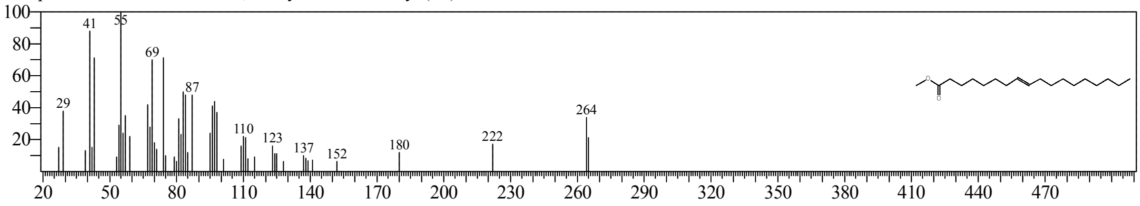

Hit#:4 Entry:109236 Library:NIST08.LIB
SI:88 Formula:C19H36O2 CAS:2777-58-4 MolWeight:296 RetIndex:2085
CompName:6-Octadecenoic acid, methyl ester, (Z)- $$ Methyl cis-6-octadecenoate $$ Methyl petroselinate $$ Methyl (6Z)-6-octadecenoate # $$

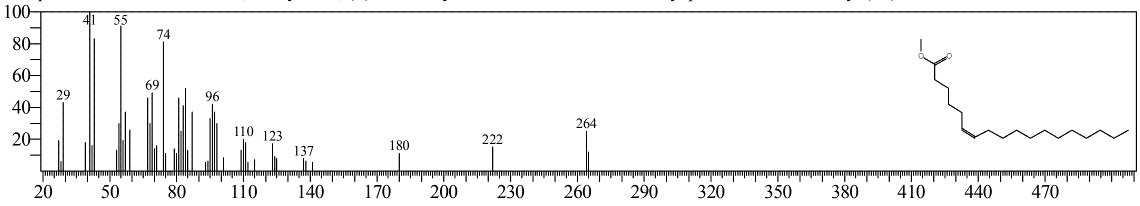

Hit#:5 Entry:109280 Library:NIST08.LIB
SI:88 Formula:C19H36O2 CAS:52355-31-4 MolWeight:296 RetIndex:2085
CompName:6-Octadecenoic acid, methyl ester $$ Methyl 6-octadecenoate $$ Methyl (6E)-6-octadecenoate # $$

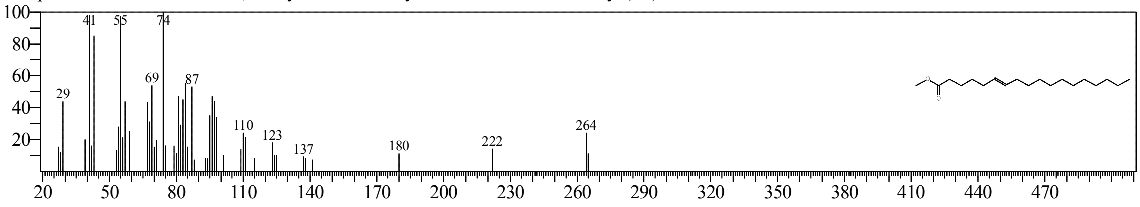

<< Target >>
Line#:13 R.Time:45.185(Scan#:8338) MassPeaks:254
RawMode:Single 45.185(8338) BasePeak:71.05(6837)
BG Mode:45.065(8314) Group 1 - Event 1

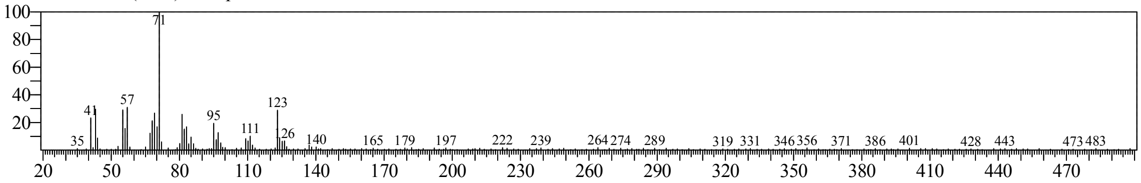

Hit#:1 Entry:24174 Library:NIST08s.LIB
SI:95 Formula:C20H40O CAS:150-86-7 MolWeight:296 RetIndex:2045
CompName:Phytol $$ 2-Hexadecen-1-ol, 3,7,11,15-tetramethyl-, [R-[R*,R*-(E)]]- $$ trans-Phytol $$ 3,7,11,15-Tetramethyl-2-hexadecen-1-ol $$ (2E)-3,7,1

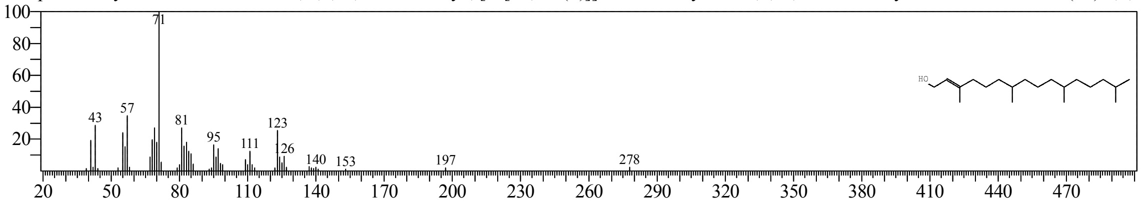

Hit#:2 Entry:421 Library:FFNSC1.3.lib
SI:91 Formula:C20 H40 O CAS:253686-88-3 MolWeight:296 RetIndex:2106
CompName:Phytol

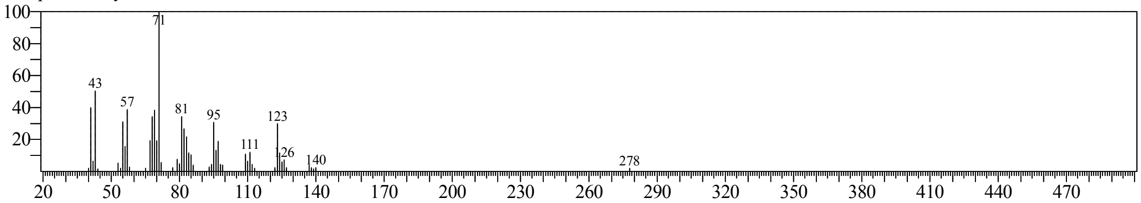

Hit#:3 Entry:18476 Library:NIST08.LIB
SI:87 Formula:C10H20O CAS:26489-01-0 MolWeight:156 RetIndex:1179
CompName:6-Octen-1-ol, 3,7-dimethyl-, (.+/-.)- $$ (.+/-.)-Citronellol $$ DL-Citronellol $$ Dihydrogeraniol $$ 6-Octen-1-ol, 3,7-dimethyl-, (.+-.)- $$ (.+-.)-

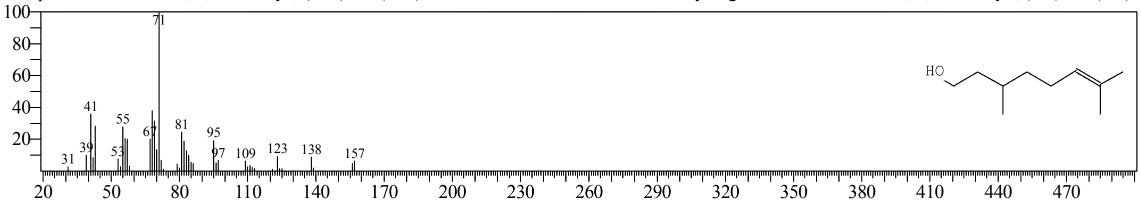

Hit#:4 Entry:18478 Library:NIST08.LIB
SI:86 Formula:C10H20O CAS:23283-97-8 MolWeight:156 RetIndex:1164
CompName:Cyclohexanol, 5-methyl-2-(1-methylethyl)-, [1S-(1.alpha.,2.beta.,5.beta.)]- $$ (1S,2R,5R)-(+)-Isomenthol $$ 2-Isopropyl-5-methylcyclohexanol

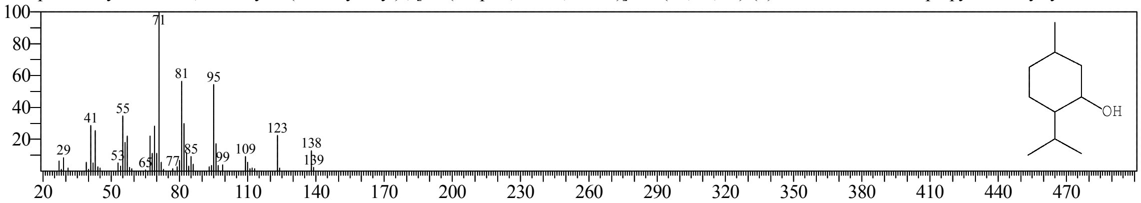

Hit#:5 Entry:9969 Library:NIST08s.LIB
SI:85 Formula:C10H20O CAS:490-99-3 MolWeight:156 RetIndex:1164
CompName:Cyclohexanol, 5-methyl-2-(1-methylethyl)-, (1.alpha.,2.beta.,5.beta.)- $$ Menthol, trans-1,3,cis-1,4- $$ Isomenthol $$ 2-Isopropyl-5-methylcycl

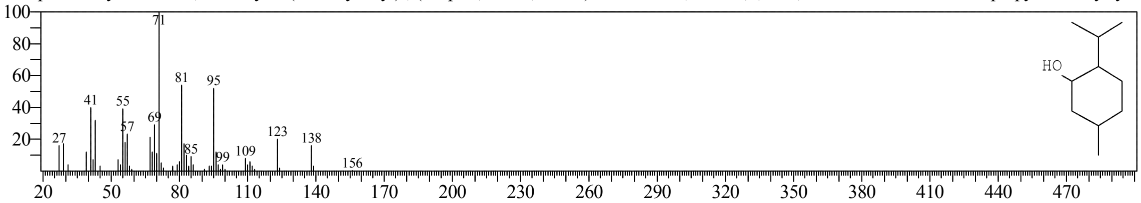

<< Target >>
Line#:14 R.Time:45.800(Scan#:8461) MassPeaks:274
RawMode:Single 45.800(8461) BasePeak:74.00(2881)
BG Mode:45.730(8447) Group 1 - Event 1

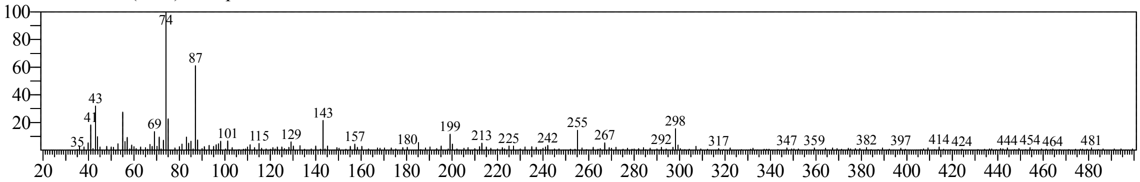

Hit#:1 Entry:110813 Library:NIST08.LIB
SI:89 Formula:C19H38O2 CAS:112-61-8 MolWeight:298 RetIndex:2077
CompName:Octadecanoic acid, methyl ester $$ Stearic acid, methyl ester $$ n-Octadecanoic acid, methyl ester $$ Kemester 9718 $$ Methyl n-octadecanoat

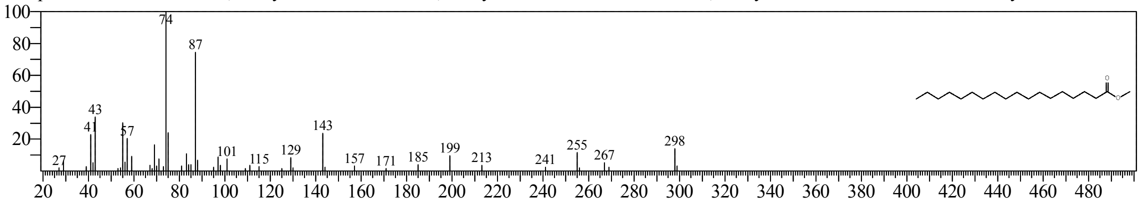

Hit#:2 Entry:24245 Library:NIST08s.LIB
SI:88 Formula:C19H38O2 CAS:5129-61-3 MolWeight:298 RetIndex:2013
CompName:Heptadecanoic acid, 16-methyl-, methyl ester $$ Methyl isostearate $$ Methyl 16-methylheptadecanoate $$

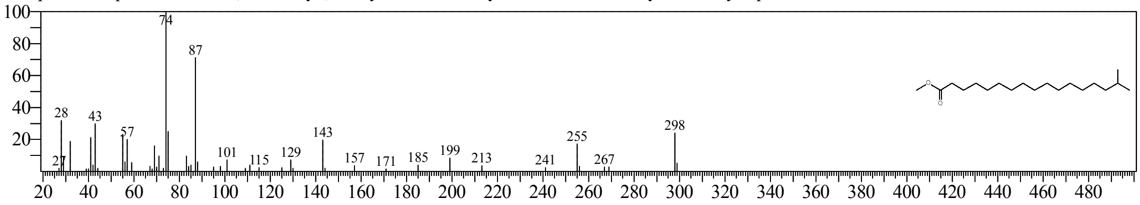

Hit#:3 Entry:70953 Library:NIST08.LIB
SI:85 Formula:C15H30O2 CAS:124-10-7 MolWeight:242 RetIndex:1680
CompName:Methyl tetradecanoate $$ Tetradecanoic acid, methyl ester $$ Myristic acid, methyl ester $$ Metholeneat 2495 $$ Methyl myristate $$ Methyl n

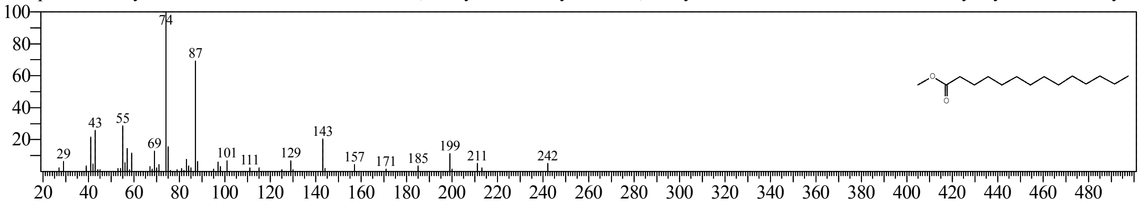

Hit#:4 Entry:80758 Library:NIST08.LIB
SI:85 Formula:C16H32O2 CAS:7132-64-1 MolWeight:256 RetIndex:1779
CompName:Pentadecanoic acid, methyl ester $$ Methyl n-pentadecanoate $$ Methyl pentadecanoate $$ n-Pentadecanoic acid methyl ester $$

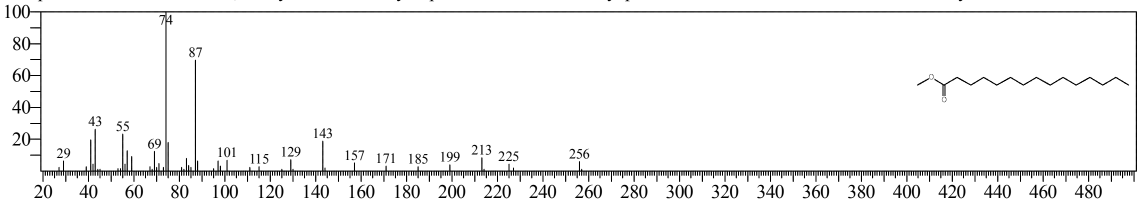

Hit#:5 Entry:70951 Library:NIST08.LIB
SI:84 Formula:C15H30O2 CAS:5129-58-8 MolWeight:242 RetIndex:1615
CompName:Tridecanoic acid, 12-methyl-, methyl ester $$ Methyl isomyristate $$ Methyl 12-methyltridecanoate $$

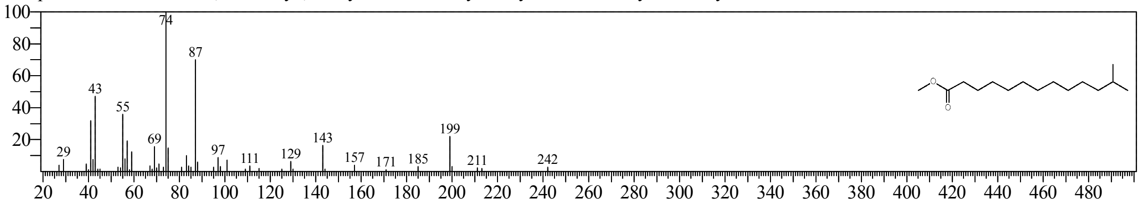

<< Target >>
Line#:15 R.Time:48.870(Scan#:9075) MassPeaks:244
RawMode:Single 48.870(9075) BasePeak:97.10(1426)
BG Mode:48.800(9061) Group 1 - Event 1

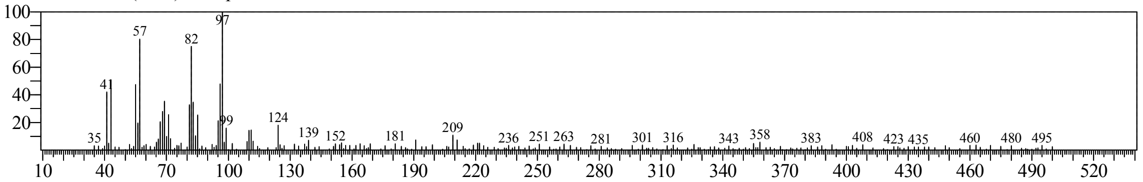

Hit#:1 Entry:179650 Library:NIST08.LIB
SI:81 Formula:C30H62O2 CAS:36645-68-8 MolWeight:454 RetIndex:3489
CompName:1,30-Triacontanediol

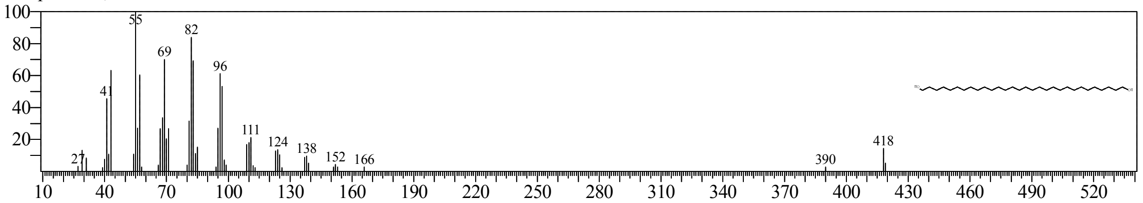

Hit#:2 Entry:186507 Library:NIST08.LIB
SI:81 Formula:C38H74 CAS:0-00-0 MolWeight:530 RetIndex:3779
CompName:1,37-Octatriacontadiene

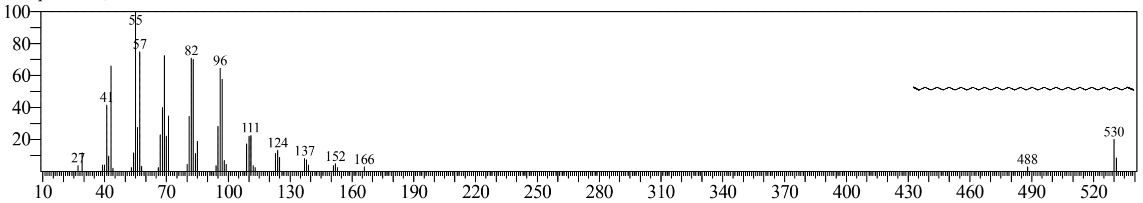

Hit#:3 Entry:60326 Library:NIST08.LIB
SI:81 Formula:C15H30O CAS:2765-11-9 MolWeight:226 RetIndex:1701
CompName:Pentadecanal-

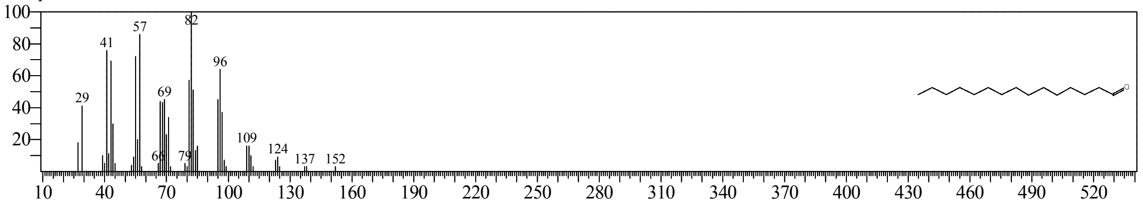

Hit#:4 Entry:89227 Library:NIST08.LIB
SI:81 Formula:C18H36O CAS:638-66-4 MolWeight:268 RetIndex:1999
CompName:Octadecanal $$ Stearaldehyde $$ Octadecyl aldehyde $$ Stearyl aldehyde $$ n-Octadecanal $$

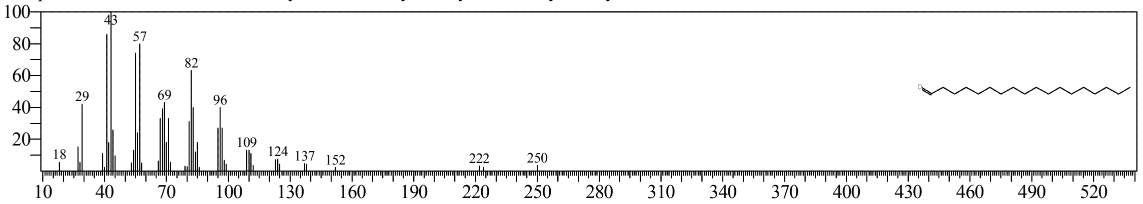

Hit#:5 Entry:16402 Library:NIST08s.LIB
SI:81 Formula:C13H26O CAS:10486-19-8 MolWeight:198 RetIndex:1502
CompName:Tridecanal $$ n-Tridecylaldehyde $$ Tridecanaldehyde $$ Tridecyl aldehyde $$ 1-Tridecanal $$ Tridecane aldehyde $$

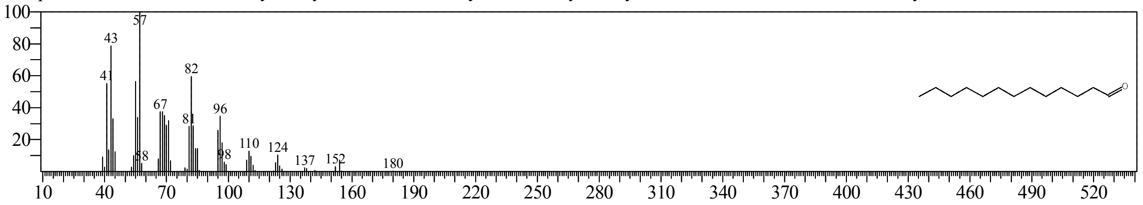

<< Target >>
Line#:16 R.Time:51.490(Scan#:9599) MassPeaks:228
RawMode:Single 51.490(9599) BasePeak:97.10(6275)
BG Mode:51.405(9582) Group 1 - Event 1

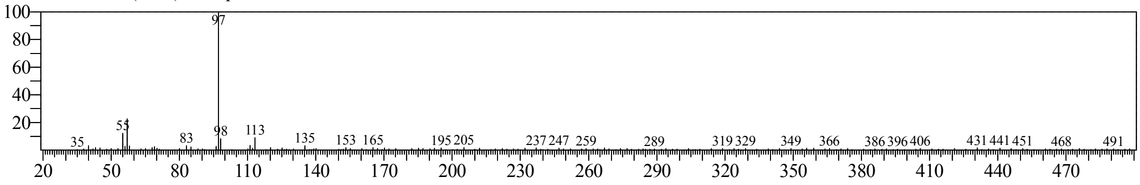

Hit#:1 Entry:58941 Library:NIST08.LIB
SI:73 Formula:C16H32 CAS:39761-73-4 MolWeight:224 RetIndex:1343
CompName:2,4,4,6,6,8,8-Heptamethyl-2-nonene

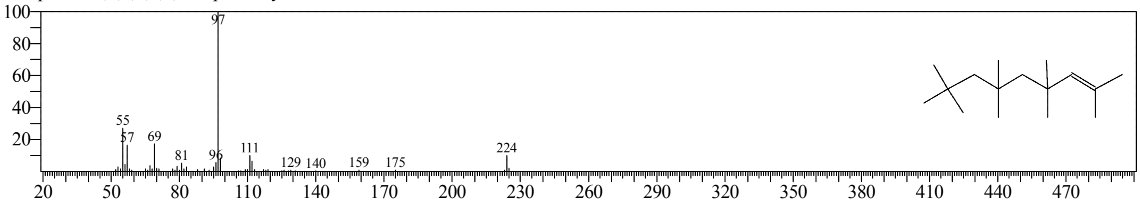

Hit#:2 Entry:2861 Library:NIST08s.LIB
SI:73 Formula:C6H12Si CAS:6224-91-5 MolWeight:112 RetIndex:545
CompName:1-(Trimethylsilyl)-1-propyne $$ 1-(Trimethylsilyl)propyne $$ Silane, trimethyl-1-propynyl- $$ Trimethyl(1-propynyl)silane # $$

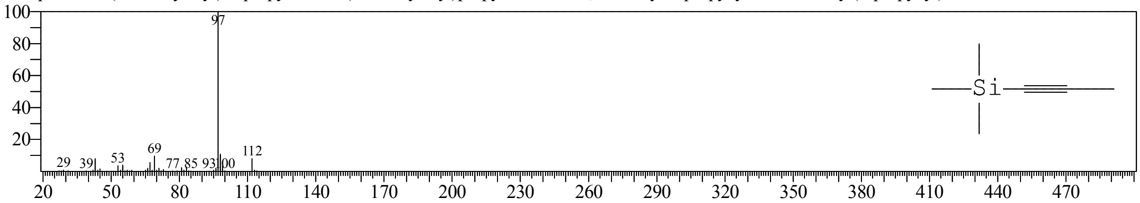

Hit#:3 Entry:158581 Library:NIST08.LIB
SI:73 Formula:C21H45O3P CAS:0-00-0 MolWeight:376 RetIndex:0
CompName:Bis(3,7-dimethyloctyl) methylphosphonate

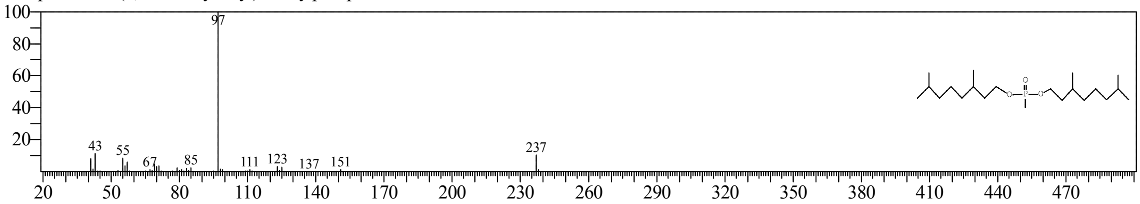

Hit#:4 Entry:125933 Library:NIST08.LIB
SI:73 Formula:C17H37O3P CAS:0-00-0 MolWeight:320 RetIndex:0
CompName:Bis(2-ethylhexyl) methylphosphonate

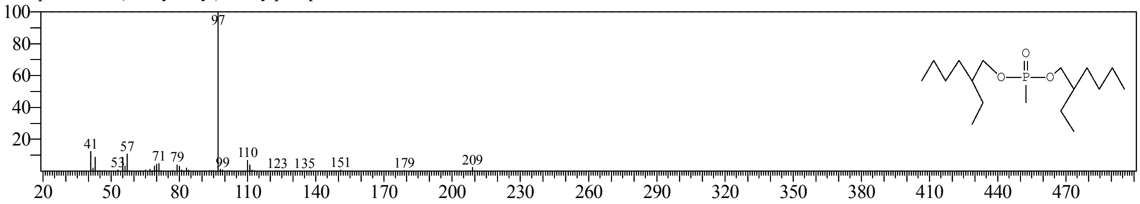

Hit#:5 Entry:163506 Library:NIST08.LIB
SI:73 Formula:C22H44O3S CAS:0-00-0 MolWeight:388 RetIndex:2895
CompName:Sulfurous acid, cyclohexylmethyl pentadecyl ester

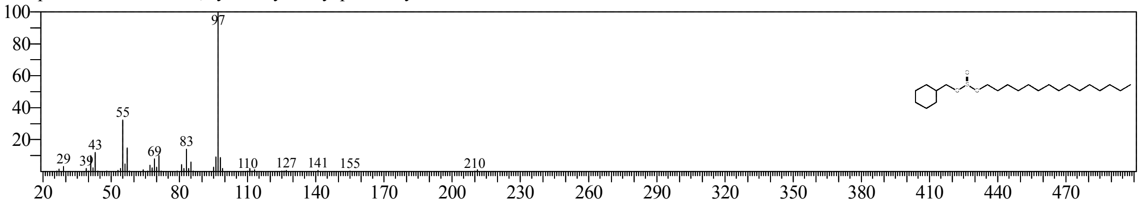

<< Target >>
Line#:17 R.Time:52.710(Scan#:9843) MassPeaks:266
RawMode:Single 52.710(9843) BasePeak:57.05(2988)
BG Mode:52.555(9812) Group 1 - Event 1

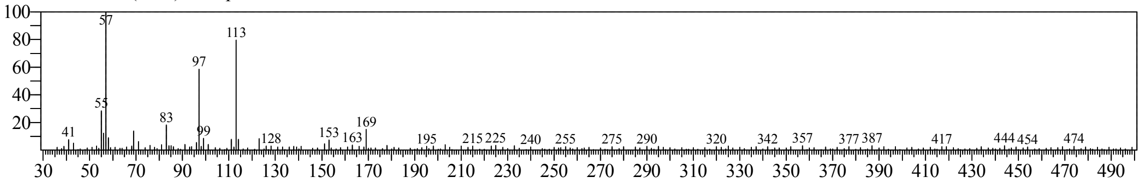

Hit#:1 Entry:58935 Library:NIST08.LIB
SI:71 Formula:C16H32 CAS:15796-04-0 MolWeight:224 RetIndex:1325
CompName:2,4,4,6,6,8,8-Heptamethyl-1-nonene

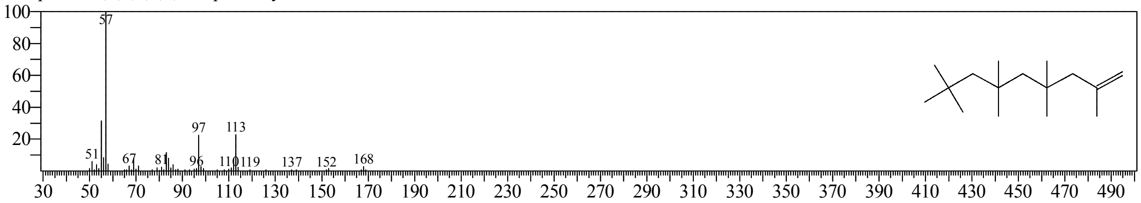

<< Target >>
Line#:18 R.Time:53.140(Scan#:9929) MassPeaks:265
RawMode:Single 53.140(9929) BasePeak:69.00(1405)
BG Mode:53.040(9909) Group 1 - Event 1

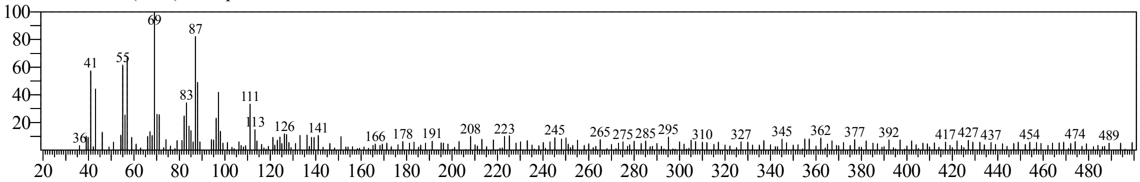

Hit#:1 Entry:128769 Library:NIST08.LIB
SI:81 Formula:C21H40O2 CAS:0-00-0 MolWeight:324 RetIndex:2243
CompName:Methacrylic acid, heptadecyl ester

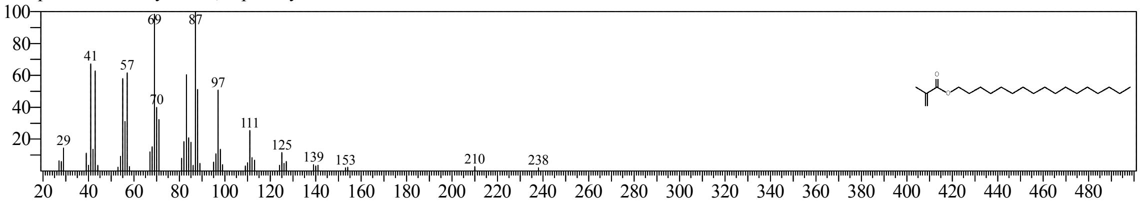

Hit#:2 Entry:137968 Library:NIST08.LIB
SI:81 Formula:C22H42O2 CAS:112-08-3 MolWeight:338 RetIndex:2343
CompName:Octadecyl methacrylate $$ Octadecyl 2-methylacrylate # $$

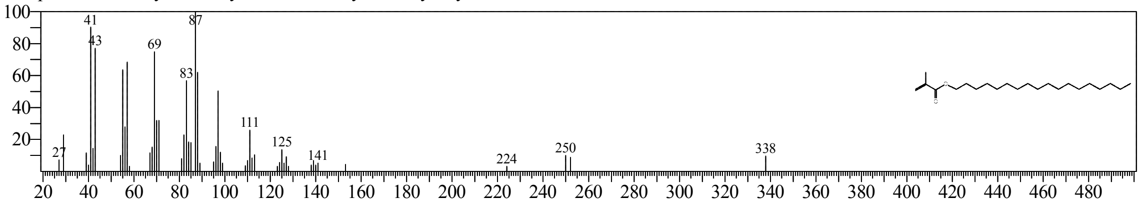

Hit#:3 Entry:119289 Library:NIST08.LIB
SI:81 Formula:C20H38O2 CAS:0-00-0 MolWeight:310 RetIndex:2144
CompName:Methacrylic acid, hexadecyl ester

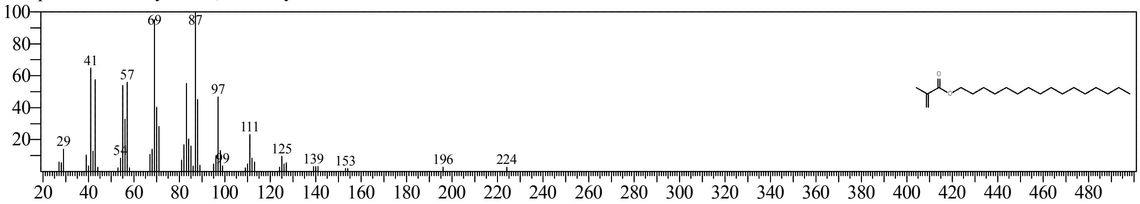

Hit#:4 Entry:99188 Library:NIST08.LIB
SI:80 Formula:C18H34O2 CAS:0-00-0 MolWeight:282 RetIndex:1945
CompName:Methacrylic acid, tetradecyl ester

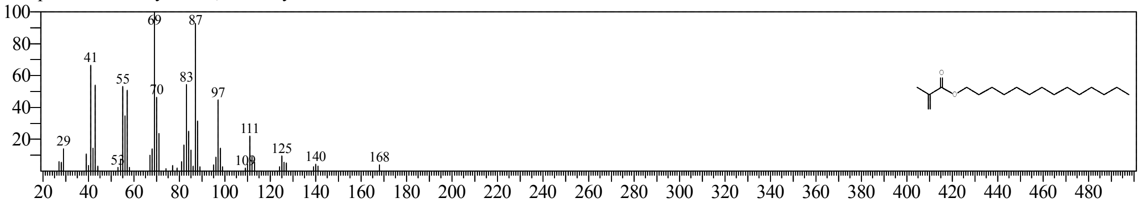

Hit#:5 Entry:109293 Library:NIST08.LIB
SI:80 Formula:C19H36O2 CAS:0-00-0 MolWeight:296 RetIndex:2044
CompName:Methacrylic acid, pentadecyl ester

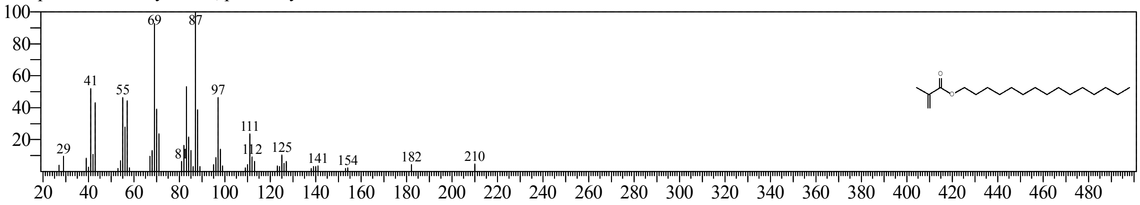

<< Target >>
Line#:19 R.Time:57.470(Scan#:10795) MassPeaks:249
RawMode:Single 57.470(10795) BasePeak:82.05(1341)
BG Mode:57.405(10782) Group 1 - Event 1

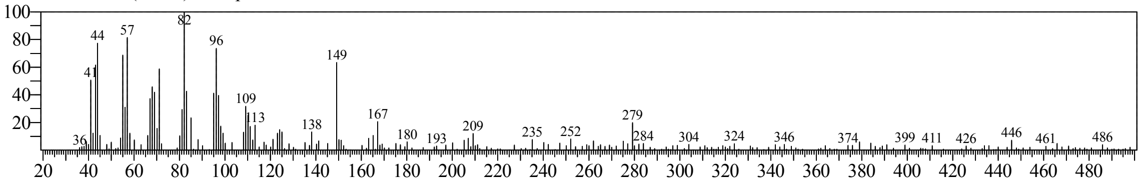

Hit#:1 Entry:87712 Library:NIST08.LIB
SI:83 Formula:C18H34O CAS:56554-87-1 MolWeight:266 RetIndex:2007
CompName:16-Octadecenal $$ (16E)-16-Octadecenal # $$

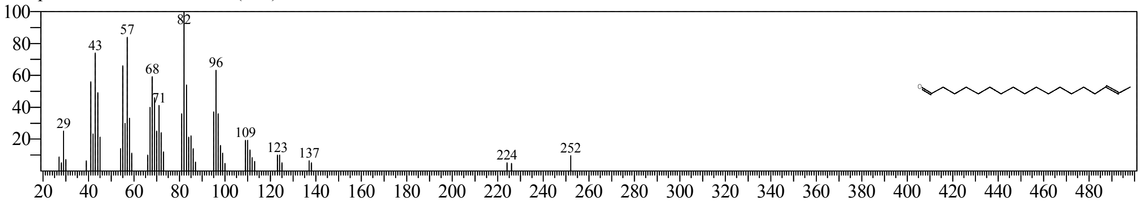

Hit#:2 Entry:20690 Library:NIST08s.LIB
SI:83 Formula:C16H32O CAS:629-80-1 MolWeight:240 RetIndex:1800
CompName:Hexadecanal $$ Palmitaldehyde $$

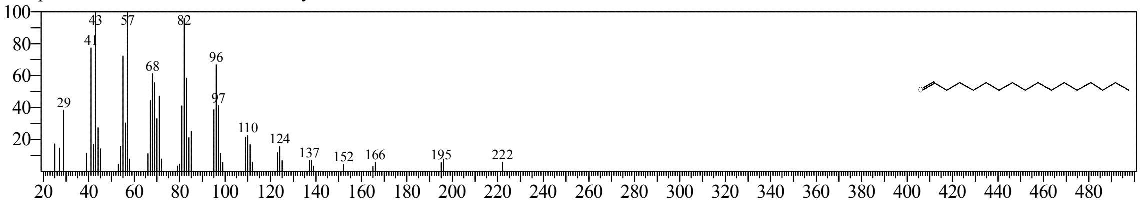

Hit#:3 Entry:99261 Library:NIST08.LIB
SI:83 Formula:C19H38O CAS:67860-04-2 MolWeight:282 RetIndex:2000
CompName:Oxirane, heptadecyl- $$ 1,2-Epoxynonadecane $$ 2-Heptadecyloxirane # $$

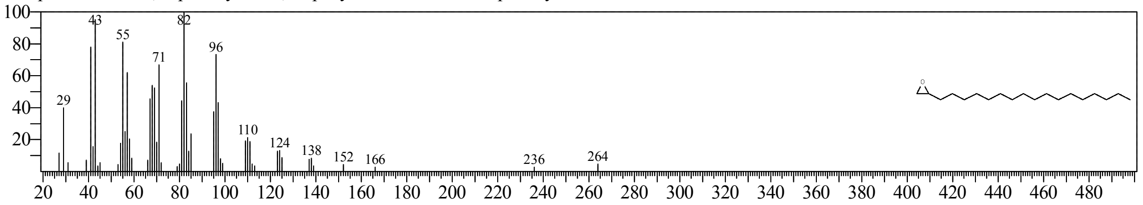

Hit#:4 Entry:87711 Library:NIST08.LIB
SI:83 Formula:C18H34O CAS:56554-86-0 MolWeight:266 RetIndex:1989
CompName:17-Octadecenal

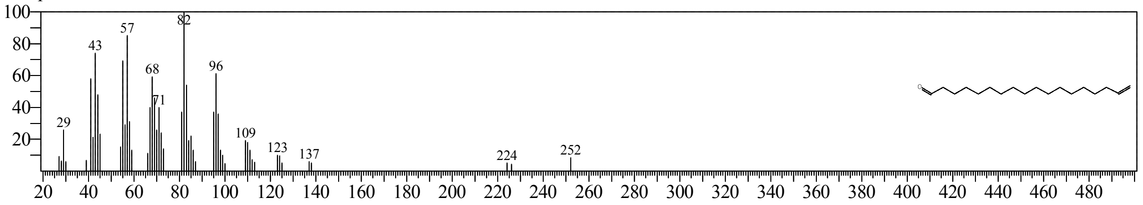

Hit#:5 Entry:22613 Library:NIST08s.LIB
SI:83 Formula:C18H36O CAS:638-66-4 MolWeight:268 RetIndex:1999
CompName:Octadecanal $$ Stearaldehyde $$ Octadecyl aldehyde $$ Stearyl aldehyde $$ n-Octadecanal $$

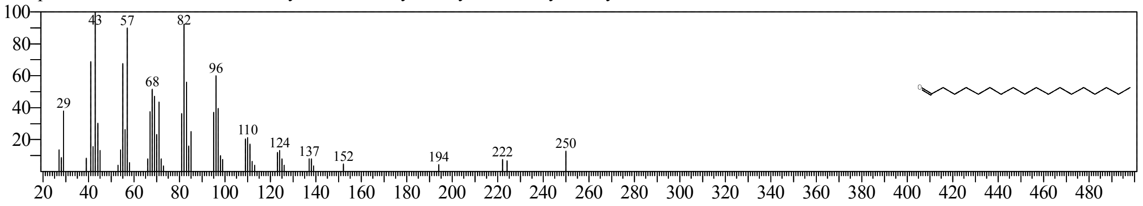

Supplement: S1 Table — (PDF) [file pone.0206982.s001.pdf]
