# Supplementary material for: Chemical composition and larvicidal activities of Azolla pinnata extracts against Aedes (Diptera:Culicidae)
Source: PLoS One. 2018 Nov 6;13(11):e0206982. doi: 10.1371/journal.pone.0206982 (PMC6219802; doi:10.1371/journal.pone.0206982)

Sample Information

LIBRARY SEARCH
ID : A
Sample Name : SOX_2000
Date :12/3/2018

Library

<< Target >>
Line#:1 R.Time:53.190(Scan#:9939) MassPeaks:318
RawMode:Single 53.190(9939) BasePeak:69.05(32233)
BG Mode:53.090(9919) Group 1 - Event 1

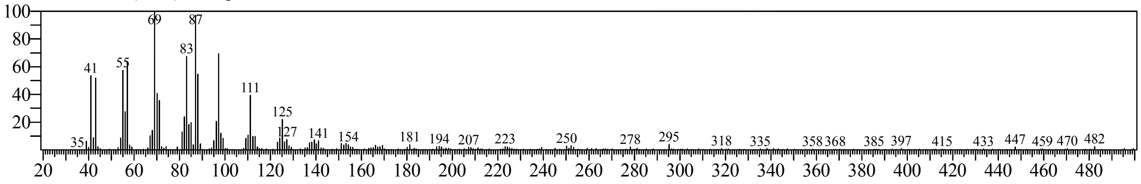

Hit#:1 Entry:128769 Library:NIST08.LIB
SI:94 Formula:C21H40O2 CAS:0-00-0 MolWeight:324 RetIndex:2243
CompName:Methacrylic acid, heptadecyl ester

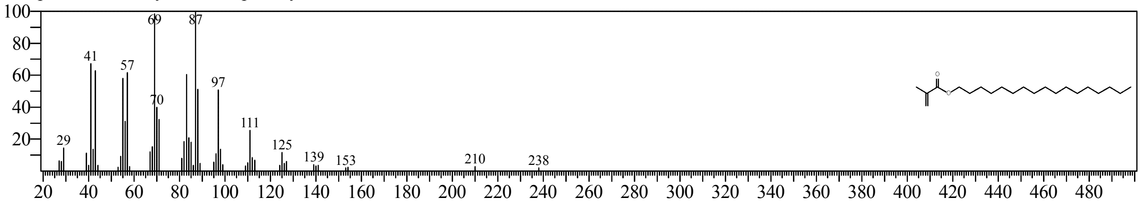

Hit#:2 Entry:146279 Library:NIST08.LIB
SI:93 Formula:C23H44O2 CAS:0-00-0 MolWeight:352 RetIndex:2442
CompName:Methacrylic acid, nonadecyl ester

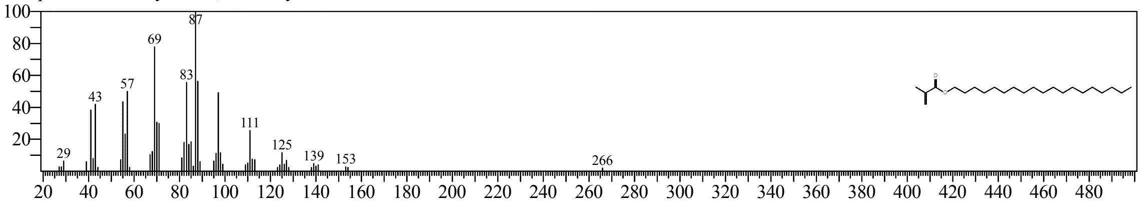

Hit#:3 Entry:119289 Library:NIST08.LIB
SI:92 Formula:C20H38O2 CAS:0-00-0 MolWeight:310 RetIndex:2144
CompName:Methacrylic acid, hexadecyl ester

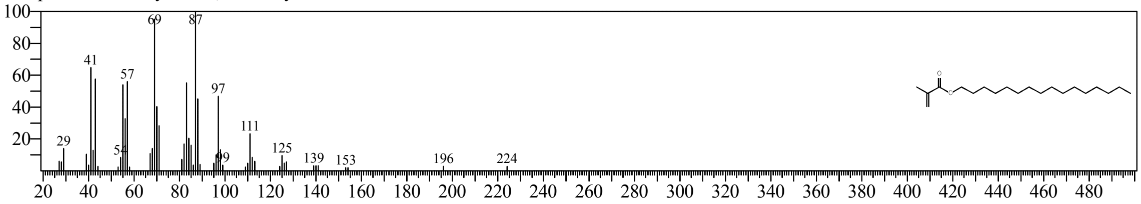

Hit#:4 Entry:109293 Library:NIST08.LIB
SI:92 Formula:C19H36O2 CAS:0-00-0 MolWeight:296 RetIndex:2044
CompName:Methacrylic acid, pentadecyl ester

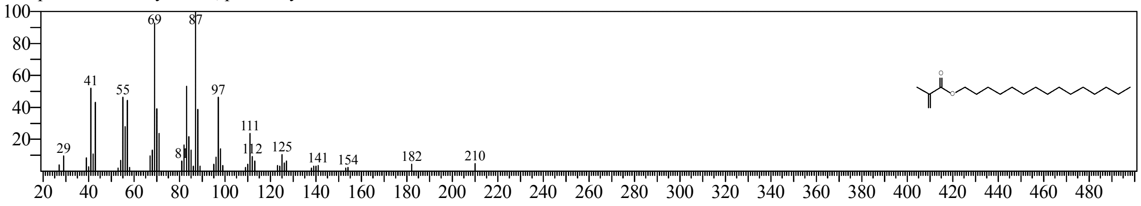

Hit#:5 Entry:137968 Library:NIST08.LIB
SI:92 Formula:C22H42O2 CAS:112-08-3 MolWeight:338 RetIndex:2343
CompName:Octadecyl methacrylate $$ Octadecyl 2-methylacrylate # $$

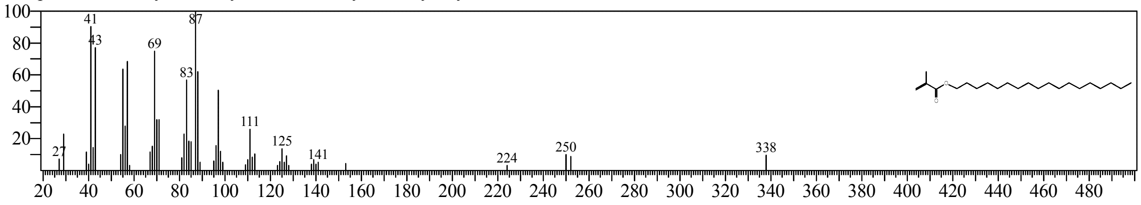

<< Target >>
Line#:2   R.Time:52.745(Scan#:9850) MassPeaks:253
RawMode:Single 52.745(9850) BasePeak:57.05(54805)
BG Mode:52.675(9836) Group 1 - Event 1

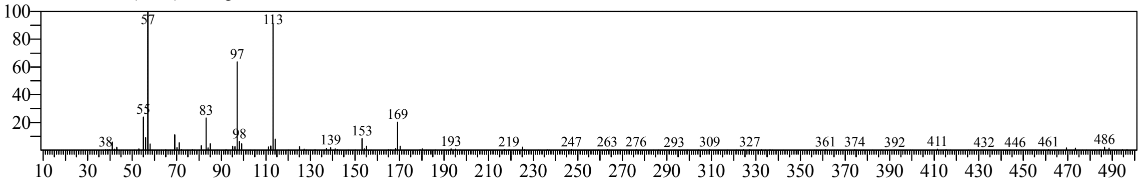

Hit#:1 Entry:58935 Library:NIST08.LIB
SI:77 Formula:C16H32 CAS:15796-04-0 MolWeight:224 RetIndex:1325
CompName:2,4,4,6,6,8,8-Heptamethyl-1-nonene

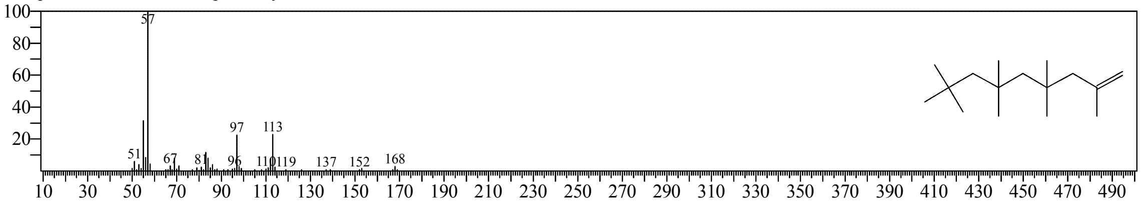

Hit#:2 Entry:41366 Library:NIST08.LIB
SI:76 Formula:C9H20B2O3 CAS:58163-56-7 MolWeight:198 RetIndex:0
CompName:Borinic acid, diethyl-, (2-ethyl-1,3,2-dioxaborolan-4-yl)methyl ester $$ (2-Ethyl-1,3,2-dioxaborolan-4-yl)methyl diethylborinate # $$

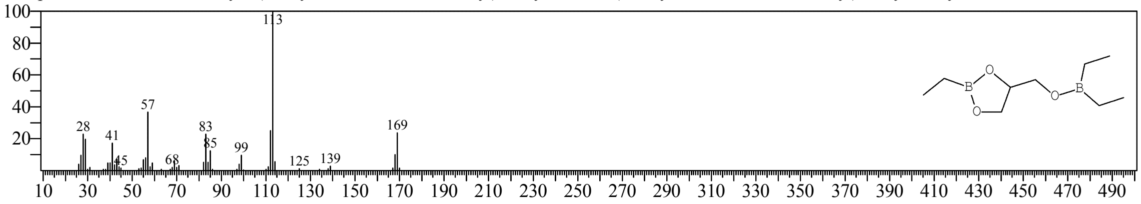

Hit#:3 Entry:24572 Library:NIST08.LIB
SI:75 Formula:C12H24 CAS:123-48-8 MolWeight:168 RetIndex:1030
CompName:3-Heptene, 2,2,4,6,6-pentamethyl- $$ 2,2,4,6,6-Pentamethylheptene-3 $$ 2,2,4,6,6-Pentamethyl-3-heptene $$ 2,2,4,6,6-Pentamethyl-3-heptene,(

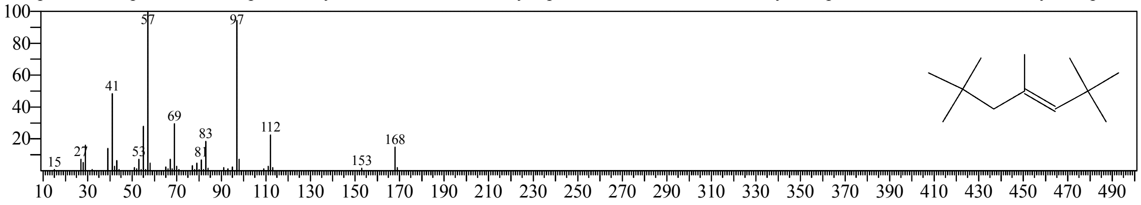

Hit#:4 Entry:165157 Library:NIST08.LIB
SI:75 Formula:C28H56 CAS:55255-73-7 MolWeight:392 RetIndex:2344
CompName:6-Tridecene, 2,2,4,10,12,12-hexamethyl-7-(3,5,5-trimethylhexyl)- $$ 2,2,4,10,12,12-Hexamethyl-7-(3,5,5-trimethylhexyl)-6-tridecene $$

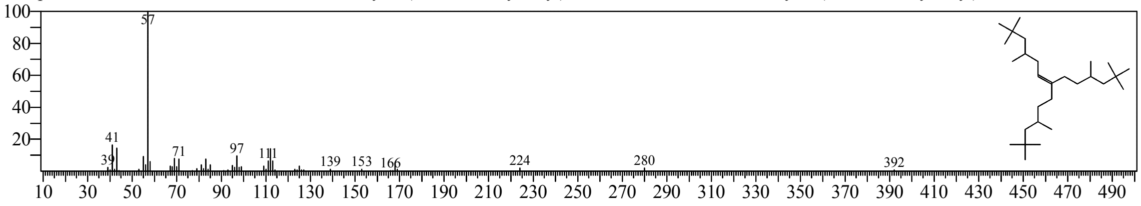

Hit#:5 Entry:178403 Library:NIST08.LIB
SI:75 Formula:C26H54O3S CAS:0-00-0 MolWeight:446 RetIndex:3165
CompName:Sulfurous acid, 2-ethylhexyl octadecyl ester

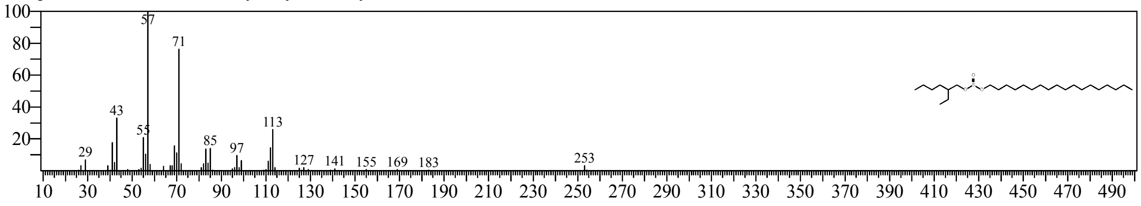

<< Target >>
Line#:3   R.Time:25.455(Scan#:4392) MassPeaks:249
RawMode:Single 25.455(4392) BasePeak:149.05(106143)
BG Mode:25.330(4367) Group 1 - Event 1

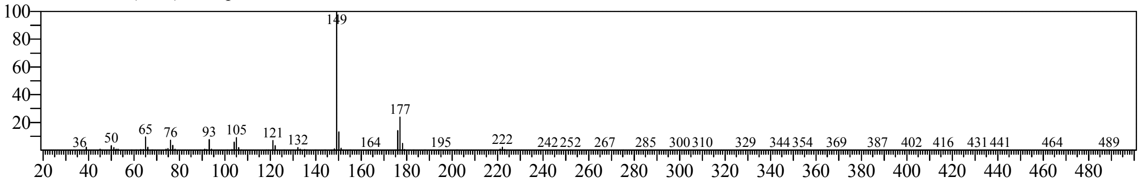

Hit#:1 Entry:57030 Library:NIST08.LIB
SI:97 Formula:C12H14O4 CAS:84-66-2 MolWeight:222 RetIndex:1639
CompName:Diethyl Phthalate $$ 1,2-Benzenedicarboxylic acid, diethyl ester $$ Phthalic acid, diethyl ester $$ o-Benzenedicarboxylic acid, diethyl ester $$

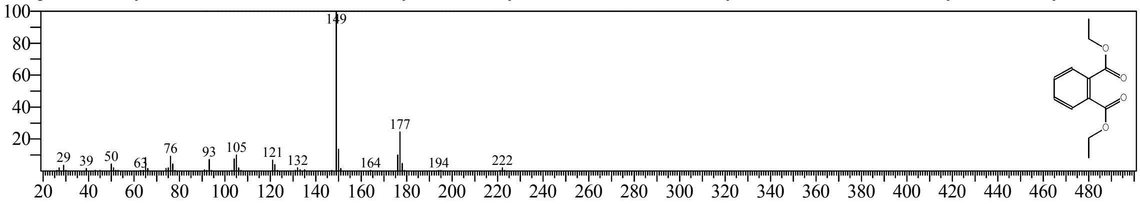

Hit#:2 Entry:1318 Library:FFNSC1.3.lib
SI:97 Formula:C21 H29 N O3 CAS:67634-12-2 MolWeight:343 RetIndex:1589
CompName:Benzoic acid <2-[[[4-(4-hydroxy-4-methylpentyl)-, 3-cyclohexen-1-yl]methylene]amino]-, methyl-> ester

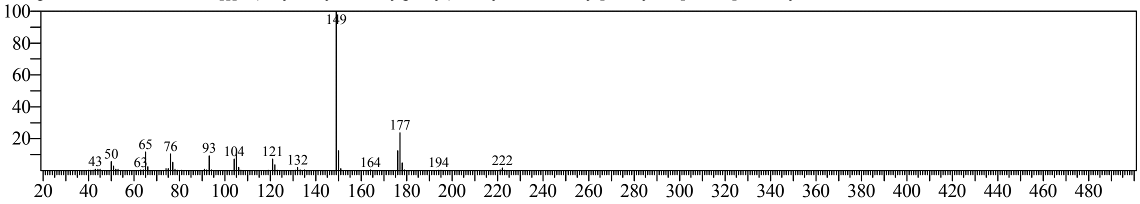

Hit#:3 Entry:66452 Library:NIST08.LIB
SI:88 Formula:C13H16O4 CAS:0-00-0 MolWeight:236 RetIndex:1674
CompName:Phthalic acid, ethyl isoporpyl ester

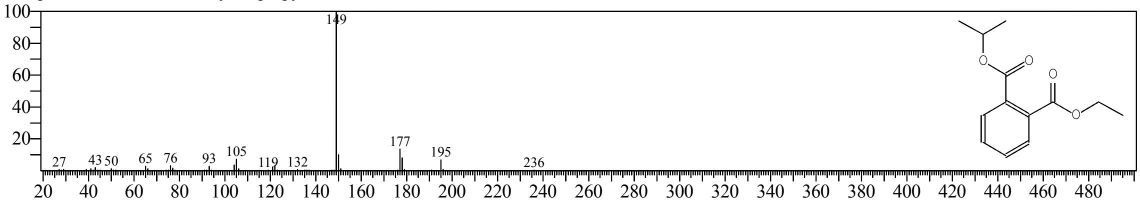

Hit#:4 Entry:81995 Library:NIST08.LIB
SI:84 Formula:C15H14O4 CAS:0-00-0 MolWeight:258 RetIndex:1943
CompName:Phthalic acid, ethyl pent-2-en-4-yn-1-yl ester

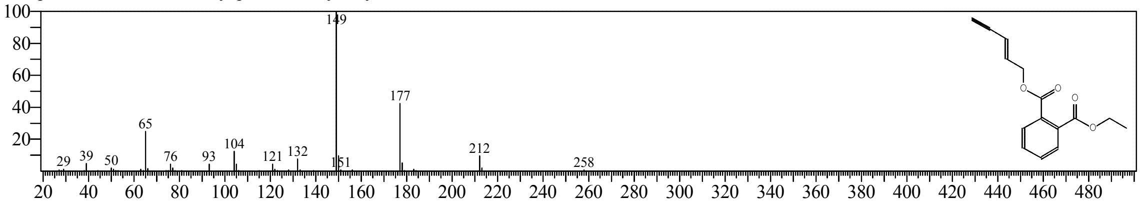

Hit#:5 Entry:120548 Library:NIST08.LIB
SI:84 Formula:C19H20O4 CAS:0-00-0 MolWeight:312 RetIndex:2361
CompName:Phthalic acid, ethyl 4-isopropylphenyl ester

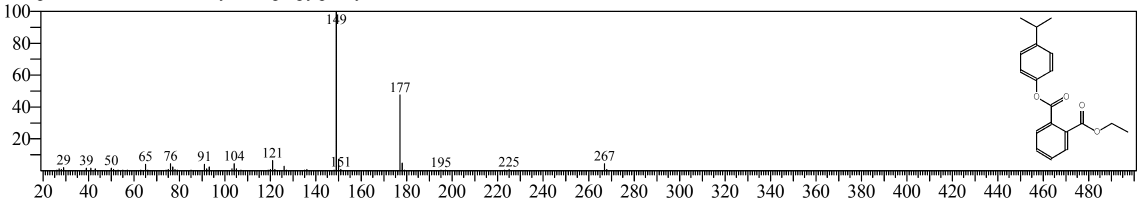

<< Target >>
Line#:4   R.Time:26.860(Scan#:4673) MassPeaks:243
RawMode:Single 26.860(4673) BasePeak:149.05(2649)
BG Mode:26.770(4655) Group 1 - Event 1

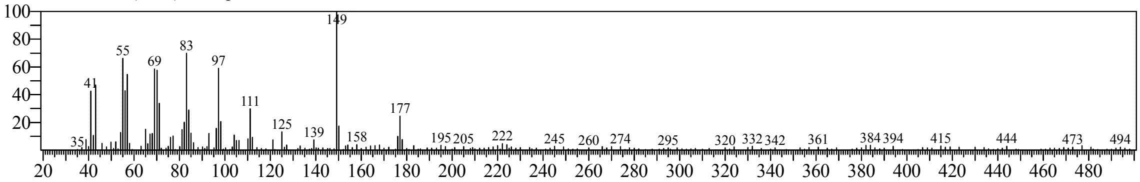

Hit#:1 Entry:124673 Library:NIST08.LIB
SI:80 Formula:C18H35ClO2 CAS:0-00-0 MolWeight:318 RetIndex:2139
CompName:Chloroacetic acid, 4-hexadecyl ester

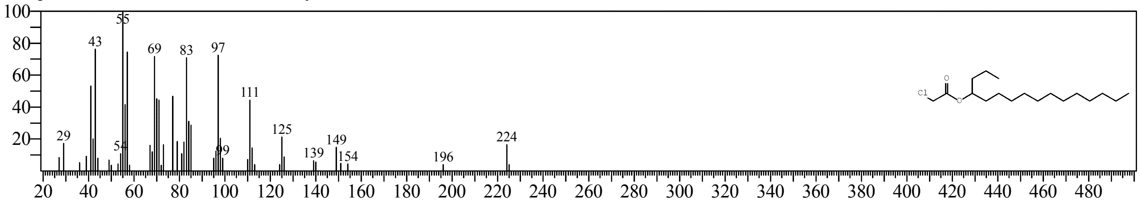

Hit#:2 Entry:145946 Library:NIST08.LIB
SI:79 Formula:C18H34Cl2O2 CAS:0-00-0 MolWeight:352 RetIndex:2259
CompName:Dichloroacetic acid, 4-hexadecyl ester

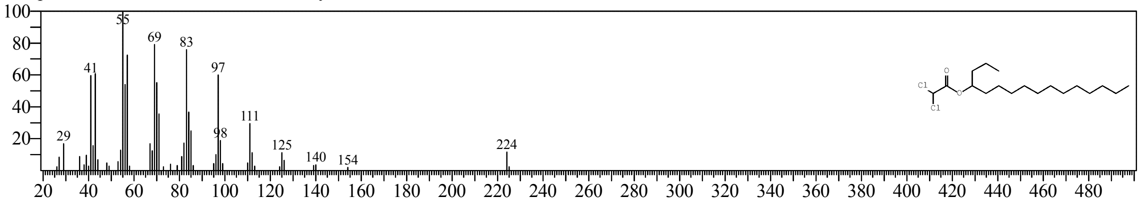

Hit#:3 Entry:124333 Library:NIST08.LIB
SI:79 Formula:C14H23F5O2 CAS:0-00-0 MolWeight:318 RetIndex:1276
CompName:Undecyl pentafluoropropionate

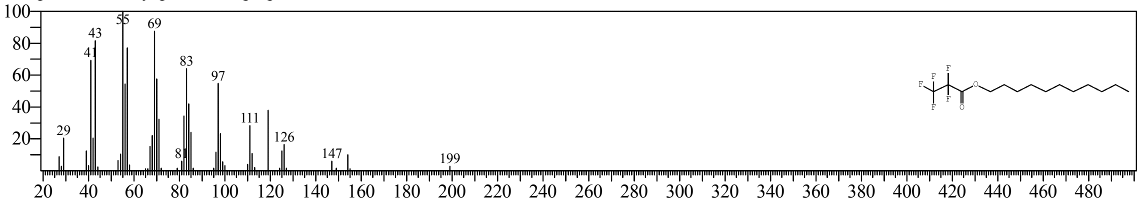

Hit#:4 Entry:19321 Library:NIST08s.LIB
SI:79 Formula:C16H32 CAS:629-73-2 MolWeight:224 RetIndex:1602
CompName:1-Hexadecene $$ .alpha.-Hexadecene $$ n-Hexadec-1-ene $$ Cetene $$ 1-Cetene $$ Hexadecylene-1 $$ Hexadec-1-ene $$ Hexadecene-1 $$ N

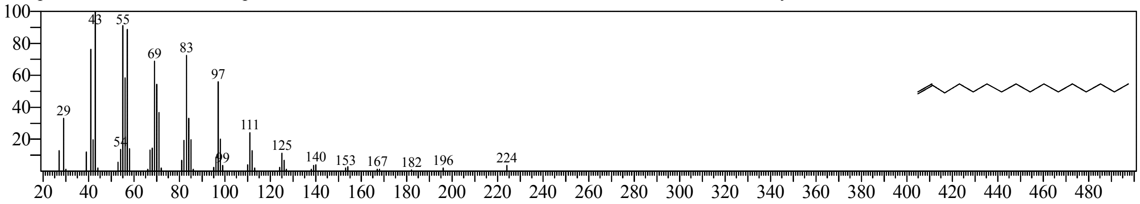

Hit#:5 Entry:14098 Library:NIST08s.LIB
SI:79 Formula:C13H26 CAS:2437-56-1 MolWeight:182 RetIndex:1304
CompName:1-Tridecene $$ n-Tridec-1-ene $$ 1-C13H26 $$ Tridecene-1 $$ .alpha.-Tridecene $$

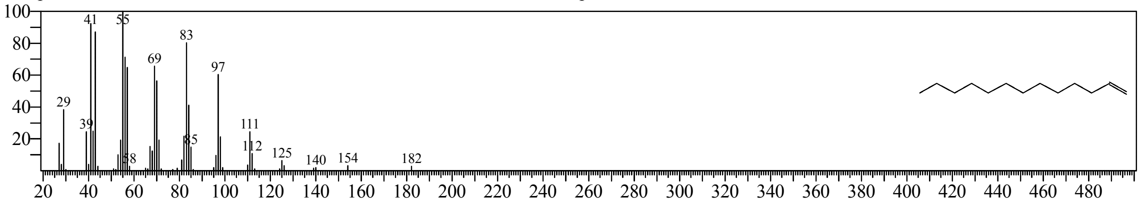

<< Target >>
Line#:5   R.Time:29.410(Scan#:5183) MassPeaks:256
RawMode:Single 29.410(5183) BasePeak:97.10(8880)
BG Mode:29.315(5164) Group 1 - Event 1

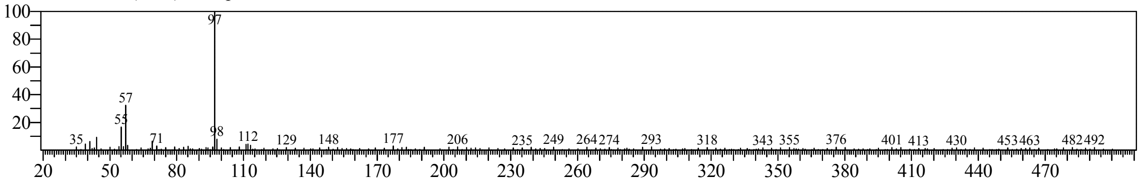

Hit#:1 Entry:150441 Library:NIST08.LIB
SI:72 Formula:C20H40O3S CAS:0-00-0 MolWeight:360 RetIndex:2696
CompName:Sulfurous acid, cyclohexylmethyl tridecyl ester

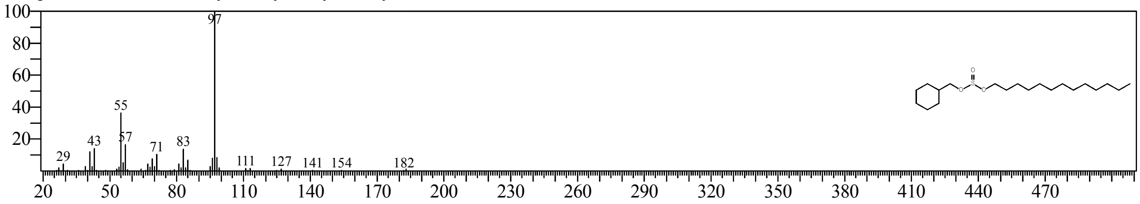

Hit#:2 Entry:163506 Library:NIST08.LIB
SI:72 Formula:C22H44O3S CAS:0-00-0 MolWeight:388 RetIndex:2895
CompName:Sulfurous acid, cyclohexylmethyl pentadecyl ester

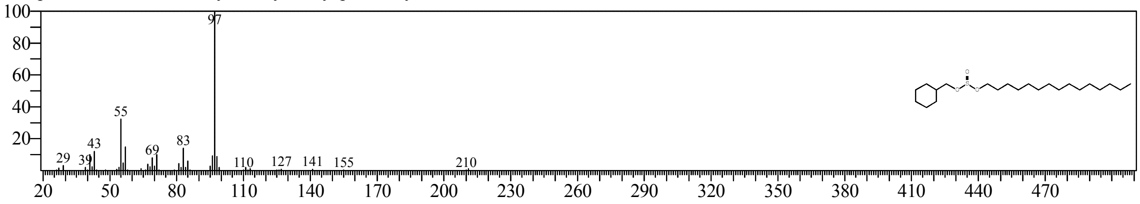

Hit#:3 Entry:65030 Library:NIST08.LIB
SI:72 Formula:C11H22O3S CAS:0-00-0 MolWeight:234 RetIndex:1801
CompName:Sulfurous acid, butyl cyclohexylmethyl ester

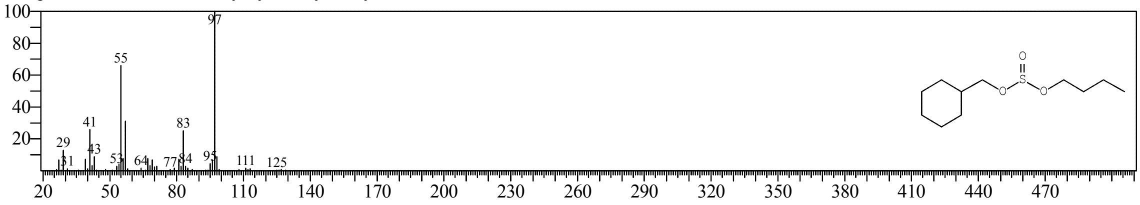

Hit#:4 Entry:94735 Library:NIST08.LIB
SI:72 Formula:C14H28O3S CAS:0-00-0 MolWeight:276 RetIndex:2100
CompName:Sulfurous acid, cyclohexylmethyl heptyl ester

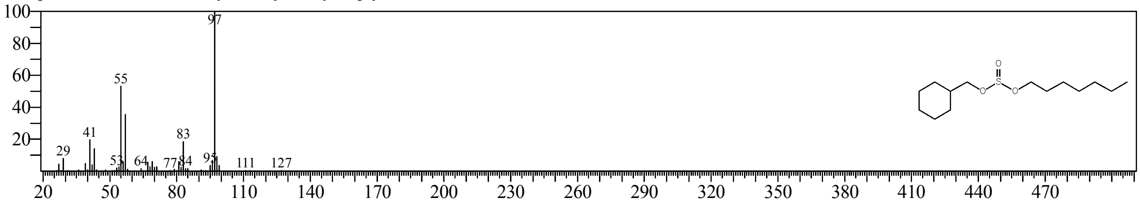

Hit#:5 Entry:84585 Library:NIST08.LIB
SI:72 Formula:C13H26O3S CAS:0-00-0 MolWeight:262 RetIndex:2000
CompName:Sulfurous acid, cyclohexylmethyl hexyl ester

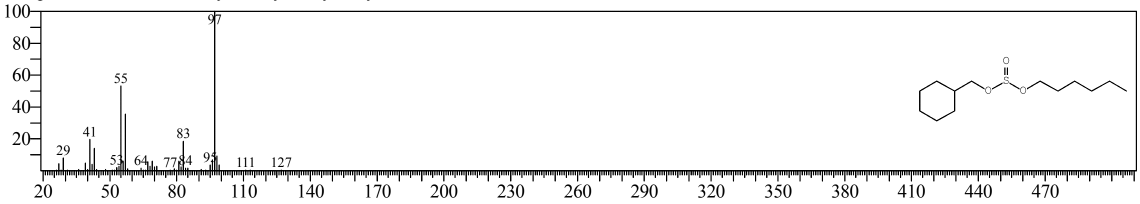

<< Target >>
Line#:6 R.Time:31.005(Scan#:5502) MassPeaks:217
RawMode:Single 31.005(5502) BasePeak:57.10(6637)
BG Mode:30.905(5482) Group 1 - Event 1

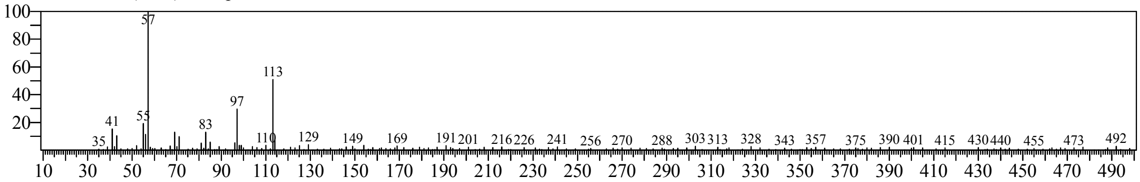

Hit#:1 Entry:19525 Library:NIST08s.LIB
SI:75 Formula:C16H34 CAS:4390-04-9 MolWeight:226 RetIndex:1294
CompName:Nonane, 2,2,4,4,6,8,8-heptamethyl- $$ 2,2,4,4,6,8,8-Heptamethylnonane $$ HMN $$ Permethyl 101A $$

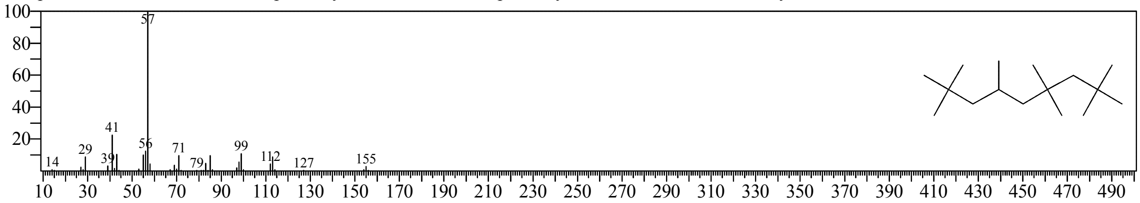

Hit#:2 Entry:178403 Library:NIST08.LIB
SI:75 Formula:C26H54O3S CAS:0-00-0 MolWeight:446 RetIndex:3165
CompName:Sulfurous acid, 2-ethylhexyl octadecyl ester

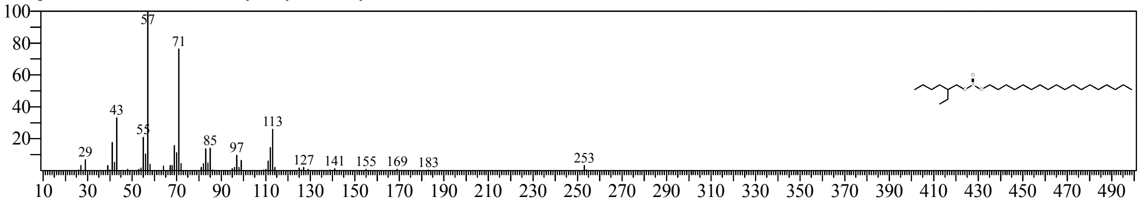

Hit#:3 Entry:172745 Library:NIST08.LIB
SI:75 Formula:C24H50O3S CAS:0-00-0 MolWeight:418 RetIndex:2966
CompName:Sulfurous acid, 2-ethylhexyl hexadecyl ester

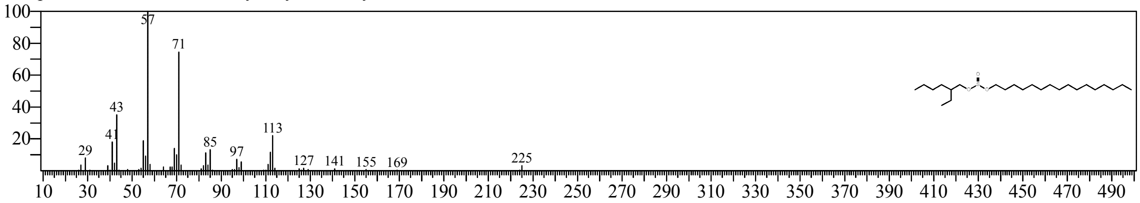

Hit#:4 Entry:169152 Library:NIST08.LIB
SI:75 Formula:C23H48O3S CAS:0-00-0 MolWeight:404 RetIndex:2867
CompName:Sulfurous acid, 2-ethylhexyl pentadecyl ester

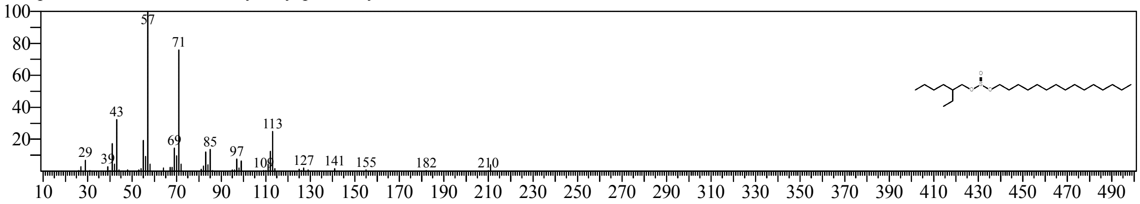

Hit#:5 Entry:175844 Library:NIST08.LIB
SI:74 Formula:C25H52O3S CAS:0-00-0 MolWeight:432 RetIndex:3065
CompName:Sulfurous acid, 2-ethylhexyl heptadecyl ester

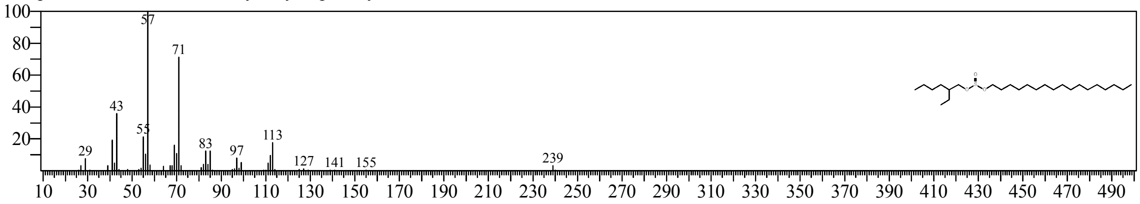

<< Target >>
Line#:7   R.Time:33.520(Scan#:6005) MassPeaks:287
RawMode:Single 33.520(6005) BasePeak:69.00(25720)
BG Mode:33.270(5955) Group 1 - Event 1

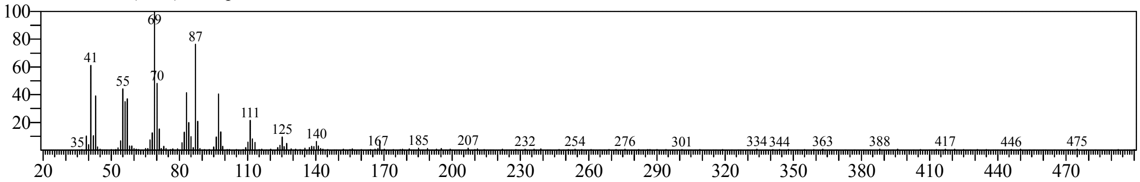

Hit#:1 Entry:79189 Library:NIST08.LIB
SI:97 Formula:C16H30O2 CAS:0-00-0 MolWeight:254 RetIndex:1746
CompName:Methacrylic acid, dodecyl ester

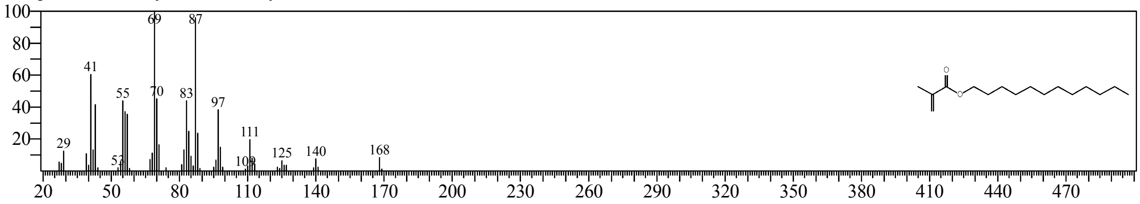

Hit#:2 Entry:99188 Library:NIST08.LIB
SI:95 Formula:C18H34O2 CAS:0-00-0 MolWeight:282 RetIndex:1945
CompName:Methacrylic acid, tetradecyl ester

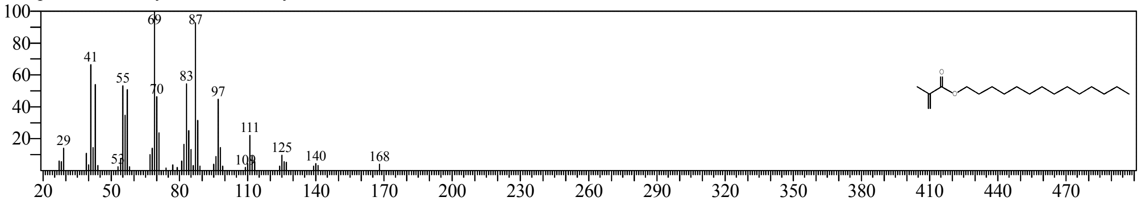

Hit#:3 Entry:79186 Library:NIST08.LIB
SI:94 Formula:C16H30O2 CAS:142-90-5 MolWeight:254 RetIndex:1746
CompName:2-Propenoic acid, 2-methyl-, dodecyl ester $$ Methacrylic acid, dodecyl ester $$ Acrylic acid, 2-methyl-, dodecyl ester $$ Dodecyl methacrylate

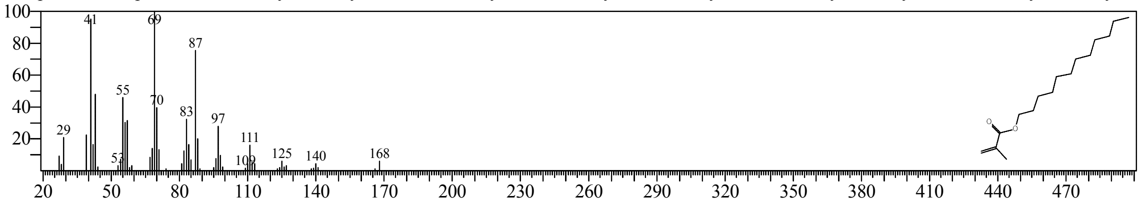

Hit#:4 Entry:60191 Library:NIST08.LIB
SI:94 Formula:C14H26O2 CAS:3179-47-3 MolWeight:226 RetIndex:1547
CompName:2-Propenoic acid, 2-methyl-, decyl ester $$ n-Decyl methacrylate $$ Methacrylic acid, decyl ester $$ Decyl methacrylate $$ Decyl 2-methylacry

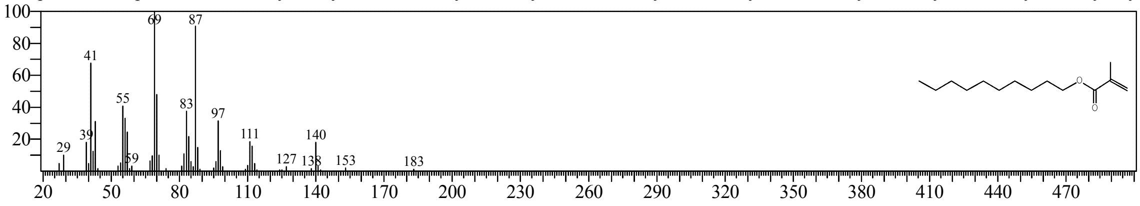

Hit#:5 Entry:109293 Library:NIST08.LIB
SI:93 Formula:C19H36O2 CAS:0-00-0 MolWeight:296 RetIndex:2044
CompName:Methacrylic acid, pentadecyl ester

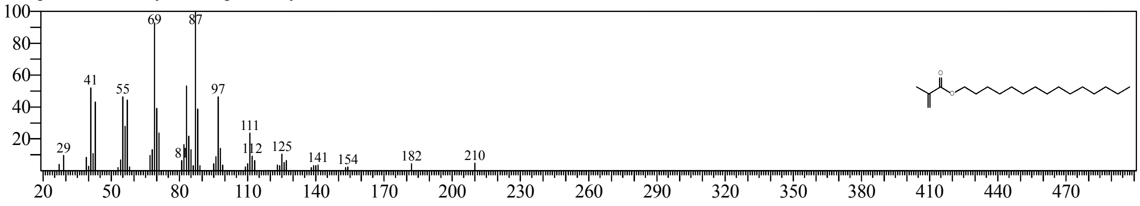

<< Target >>
Line#:8 R.Time:33.795(Scan#:6060) MassPeaks:252
RawMode:Single 33.795(6060) BasePeak:69.05(8268)
BG Mode:33.730(6047) Group 1 - Event 1

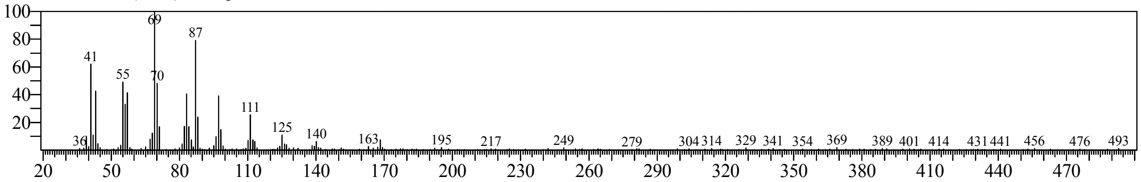

Hit#:1 Entry:79189 Library:NIST08.LIB
SI:96 Formula:C16H30O2 CAS:0-00-0 MolWeight:254 RetIndex:1746
CompName:Methacrylic acid, dodecyl ester

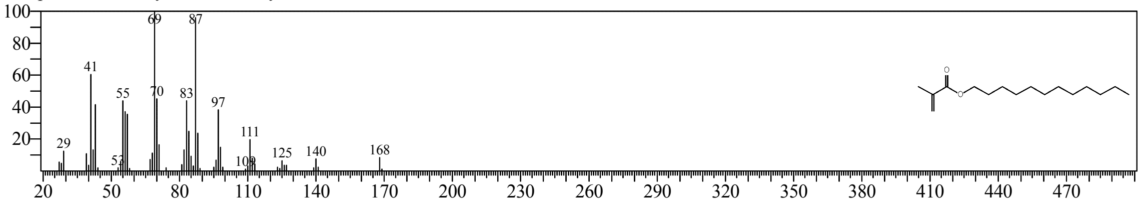

Hit#:2 Entry:99188 Library:NIST08.LIB
SI:94 Formula:C18H34O2 CAS:0-00-0 MolWeight:282 RetIndex:1945
CompName:Methacrylic acid, tetradecyl ester

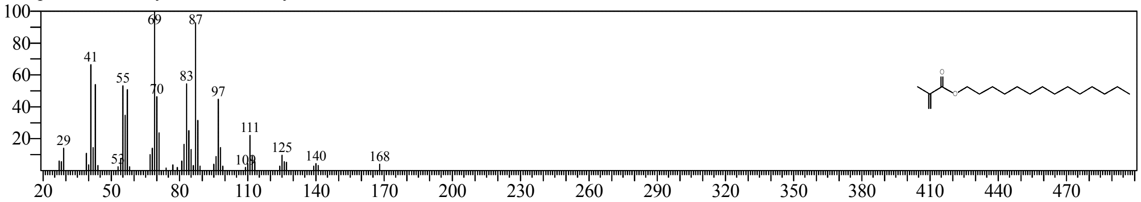

Hit#:3 Entry:21688 Library:NIST08s.LIB
SI:93 Formula:C16H30O2 CAS:142-90-5 MolWeight:254 RetIndex:1746
CompName:2-Propenoic acid, 2-methyl-, dodecyl ester $$ Methacrylic acid, dodecyl ester $$ Acrylic acid, 2-methyl-, dodecyl ester $$ Dodecyl methacrylate

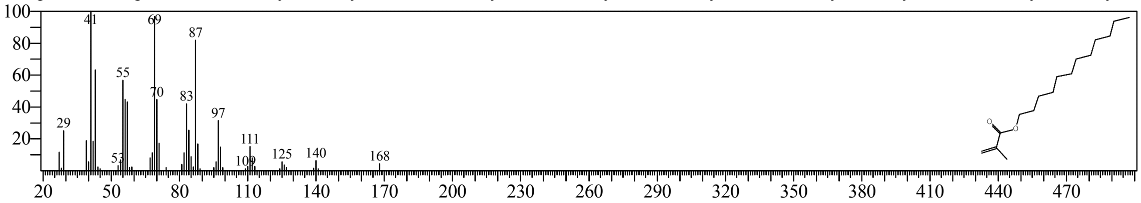

Hit#:4 Entry:109293 Library:NIST08.LIB
SI:93 Formula:C19H36O2 CAS:0-00-0 MolWeight:296 RetIndex:2044
CompName:Methacrylic acid, pentadecyl ester

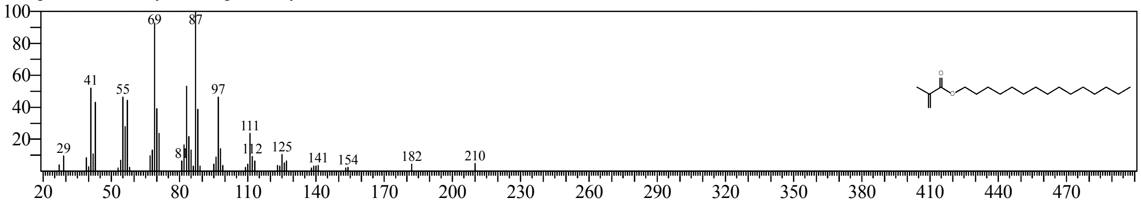

Hit#:5 Entry:119289 Library:NIST08.LIB
SI:93 Formula:C20H38O2 CAS:0-00-0 MolWeight:310 RetIndex:2144
CompName:Methacrylic acid, hexadecyl ester

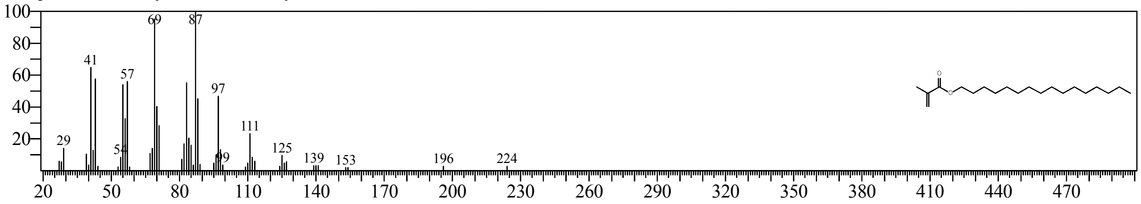

<< Target >>
Line#:9   R.Time:34.480(Scan#:6197) MassPeaks:224
RawMode:Single 34.480(6197) BasePeak:70.05(2228)
BG Mode:34.405(6182) Group 1 - Event 1

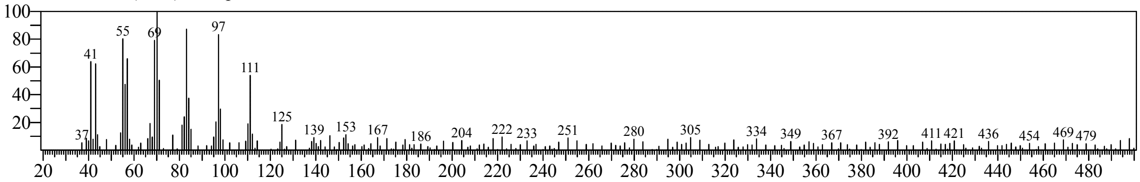

Hit#:1 Entry:22496 Library:NIST08s.LIB
SI:87 Formula:C19H38 CAS:18435-45-5 MolWeight:266 RetIndex:1900
CompName:1-Nonadecene

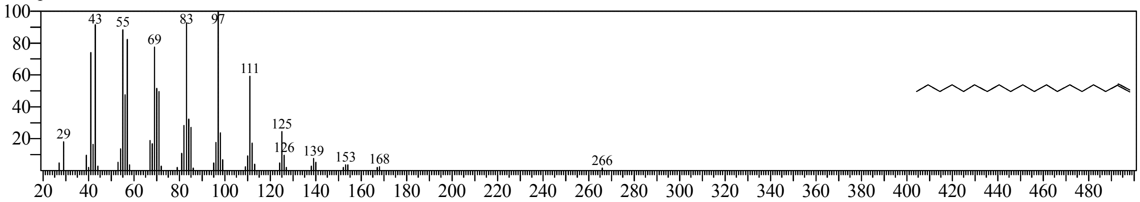

Hit#:2 Entry:61696 Library:NIST08.LIB
SI:86 Formula:C15H32O CAS:629-76-5 MolWeight:228 RetIndex:1755
CompName:n-Pentadecanol $$ n-1-Pentadecanol $$ Pentadecanol $$ Neodol 5 $$ 1-Pentadecanol $$ Pentadecan-1-ol $$ Pentadecyl alcohol $$

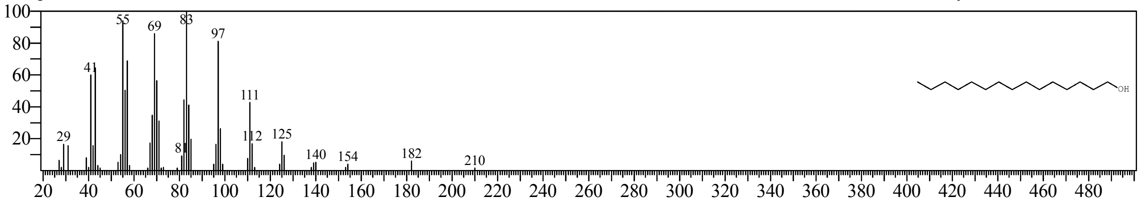

Hit#:3 Entry:100901 Library:NIST08.LIB
SI:86 Formula:C19H40O CAS:1454-84-8 MolWeight:284 RetIndex:2153
CompName:n-Nonadecanol-1 $$ 1-Nonadecanol $$ Nonadecanol $$ Nonadecan-1-ol $$ Nonadecyl alcohol $$

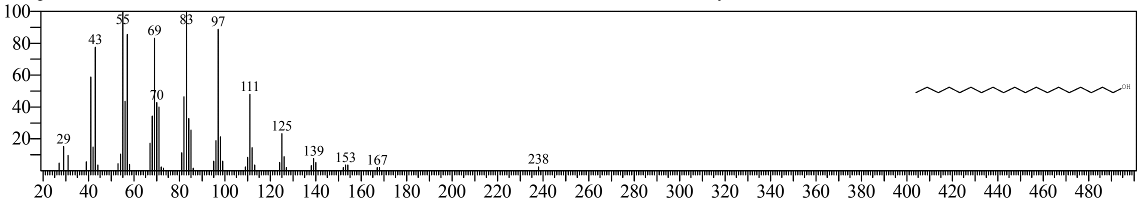

Hit#:4 Entry:127533 Library:NIST08.LIB
SI:86 Formula:C23H46 CAS:27519-02-4 MolWeight:322 RetIndex:2315
CompName:9-Tricosene, (Z)- $$ (Z)-9-Tricosene $$ cis-9-Tricosene $$ Muscalure $$ (9Z)-Tricosene $$ (9Z)-9-Tricosene # $$

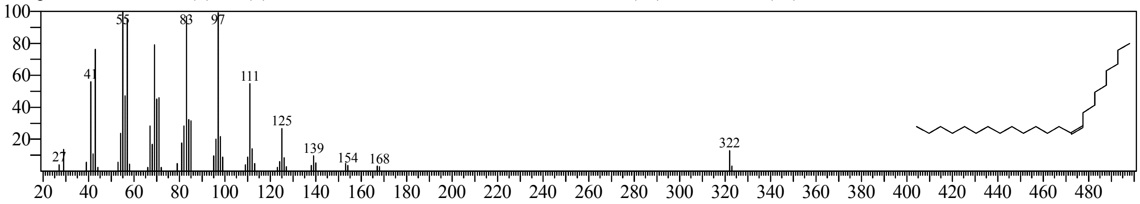

Hit#:5 Entry:25469 Library:NIST08s.LIB
SI:85 Formula:C22H46O CAS:661-19-8 MolWeight:326 RetIndex:2451
CompName:Behenic alcohol $$ 1-Docosanol $$ Docosyl alcohol $$ Docosanol-(1) $$ Cachalot BE-22 $$ Dehydag wax 22 (lanette) $$ Emery 3304 $$ Lo

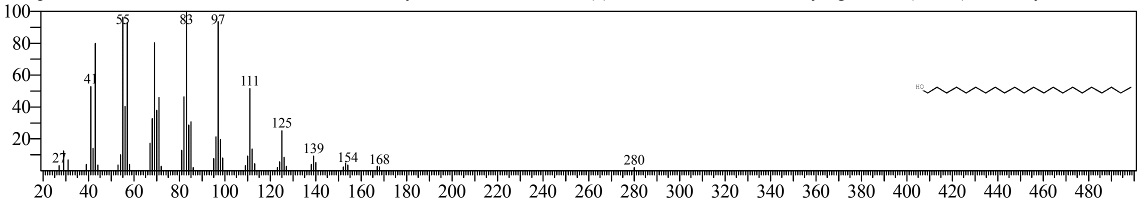

<< Target >>
Line#:10 R.Time:35.980(Scan#:6497) MassPeaks:268
RawMode:Single 35.980(6497) BasePeak:68.05(17547)
BG Mode:35.890(6479) Group 1 - Event 1

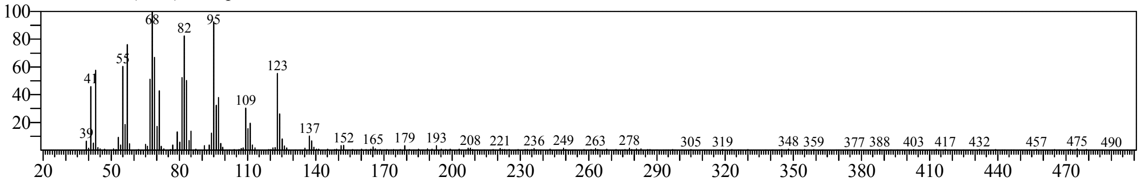

Hit#:1 Entry:908 Library:FFNSC1.3.lib
SI:94 Formula:C20 H38 CAS:504-96-1 MolWeight:278 RetIndex:1836
CompName:Neophytadiene

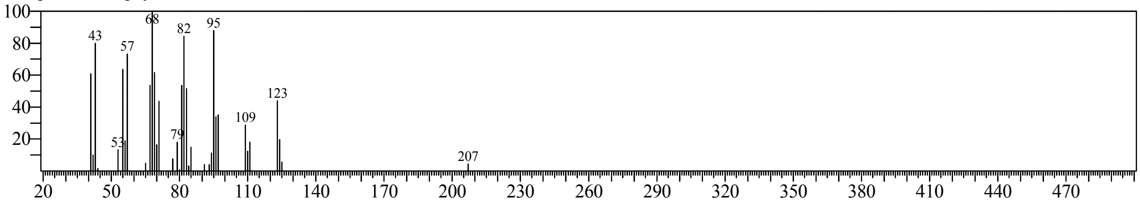

Hit#:2 Entry:109355 Library:NIST08.LIB
SI:90 Formula:C20H40O CAS:102608-53-7 MolWeight:296 RetIndex:2045
CompName:3,7,11,15-Tetramethyl-2-hexadecen-1-ol $$ (2E)-3,7,11,15-Tetramethyl-2-hexadecen-1-ol # $$

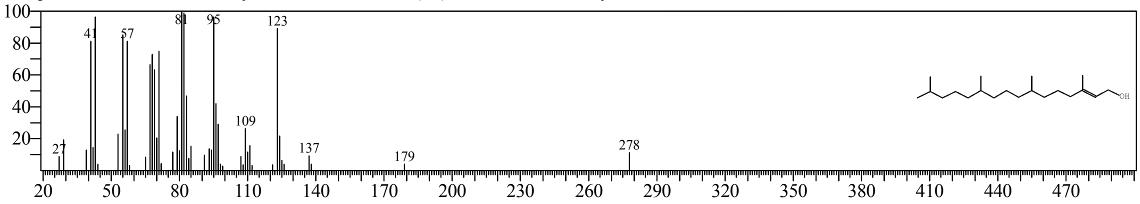

Hit#:3 Entry:22616 Library:NIST08s.LIB
SI:88 Formula:C18H36O CAS:143-28-2 MolWeight:268 RetIndex:2061
CompName:Oleyl Alcohol $$ 9-Octadecen-1-ol, (Z)- $$ cis-9-Octadecen-1-ol $$ cis-9-Octadecenyl Alcohol $$ Adol 320 $$ Adol 85 $$ Atalco O $$ Cachal

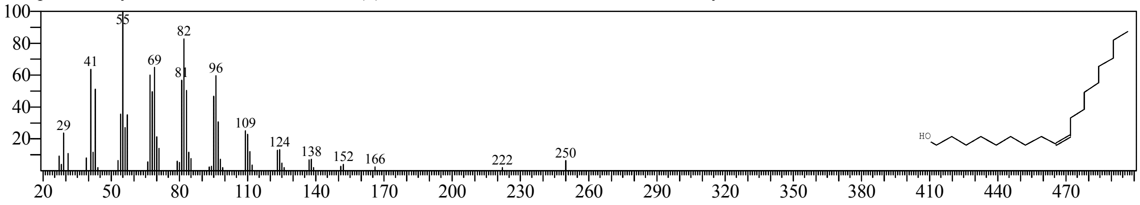

Hit#:4 Entry:1115 Library:FFNSC1.3.lib
SI:87 Formula:C22 H42 O2 CAS:76337-16-1 MolWeight:338 RetIndex:2212
CompName:Phytol acetate

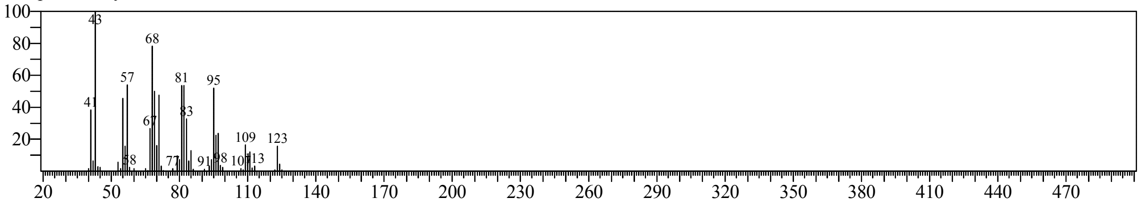

Hit#:5 Entry:89240 Library:NIST08.LIB
SI:87 Formula:C18H36O CAS:7390-81-0 MolWeight:268 RetIndex:1901
CompName:Oxirane, hexadecyl- $$ 1,2-Epoxyoctadecane $$ 2-Hexadecyloxirane # $$

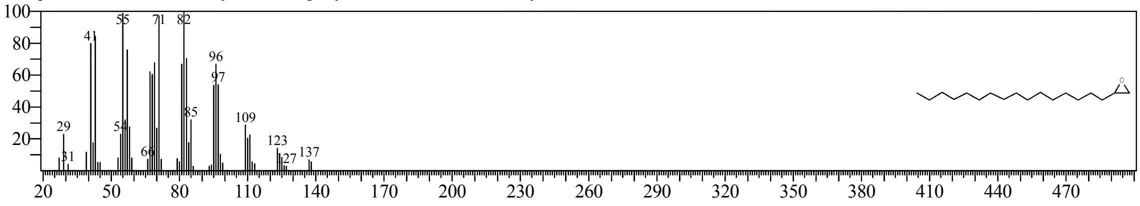

<< Target >>
Line#:11 R.Time:36.865(Scan#:6674) MassPeaks:246
RawMode:Single 36.865(6674) BasePeak:82.10(3450)
BG Mode:36.775(6656) Group 1 - Event 1

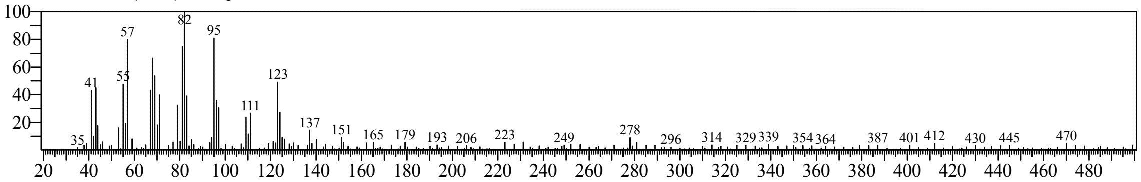

Hit#:1 Entry:908 Library:FFNSC1.3.lib
SI:88 Formula:C20 H38 CAS:504-96-1 MolWeight:278 RetIndex:1836
CompName:Neophytadiene

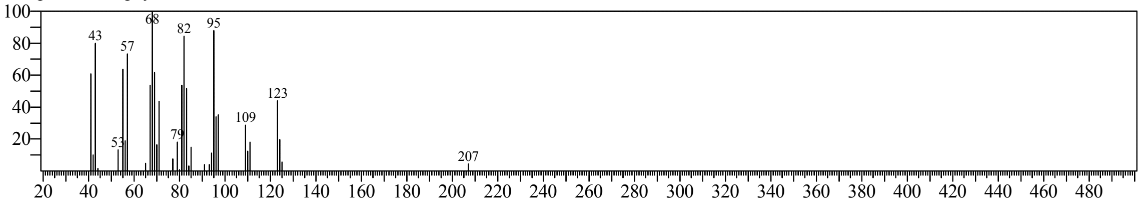

Hit#:2 Entry:109355 Library:NIST08.LIB
SI:88 Formula:C20H40O CAS:102608-53-7 MolWeight:296 RetIndex:2045
CompName:3,7,11,15-Tetramethyl-2-hexadecen-1-ol $$ (2E)-3,7,11,15-Tetramethyl-2-hexadecen-1-ol # $$

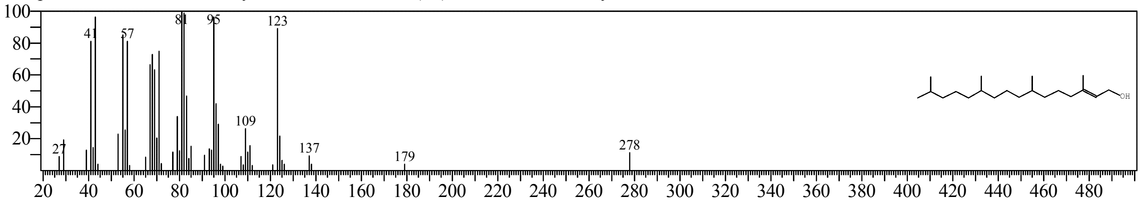

Hit#:3 Entry:96436 Library:NIST08.LIB
SI:84 Formula:C20H38 CAS:61886-66-6 MolWeight:278 RetIndex:2027
CompName:3-Eicosyne $$ 3-Icosyne # $$

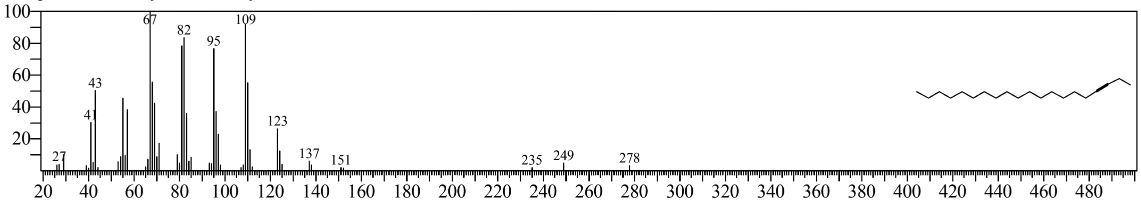

Hit#:4 Entry:60326 Library:NIST08.LIB
SI:83 Formula:C15H30O CAS:2765-11-9 MolWeight:226 RetIndex:1701
CompName:Pentadecanal-

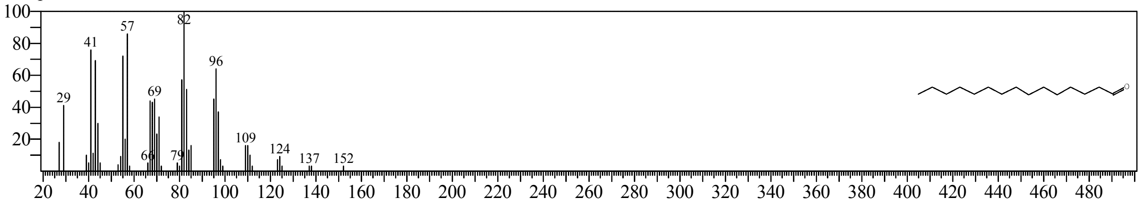

Hit#:5 Entry:89232 Library:NIST08.LIB
SI:83 Formula:C18H36O CAS:143-28-2 MolWeight:268 RetIndex:2061
CompName:Oleyl Alcohol $$ 9-Octadecen-1-ol, (Z)- $$ cis-9-Octadecen-1-ol $$ cis-9-Octadecenyl Alcohol $$ Adol 320 $$ Adol 85 $$ Atalco O $$ Cachal

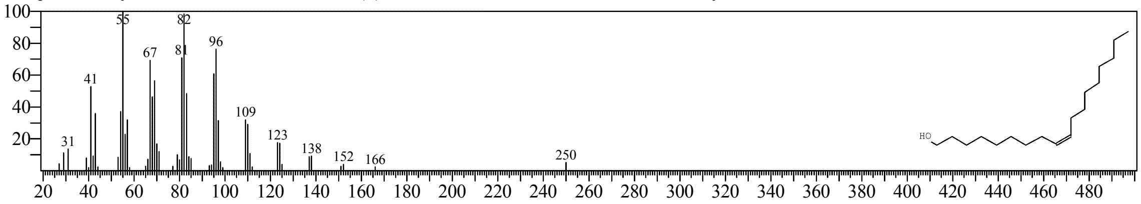

<< Target >>
Line#:12 R.Time:37.320(Scan#:6765) MassPeaks:265
RawMode:Single 37.320(6765) BasePeak:69.05(9137)
BG Mode:37.205(6742) Group 1 - Event 1

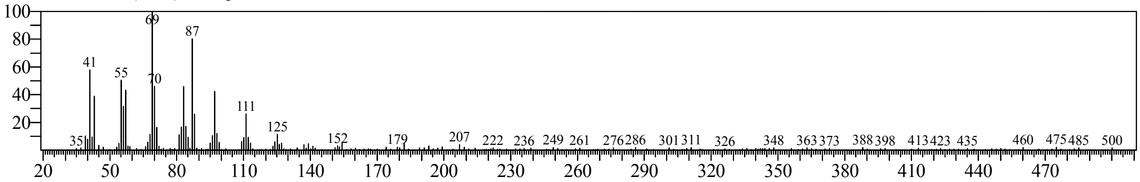

Hit#:1 Entry:109293 Library:NIST08.LIB
SI:93 Formula:C19H36O2 CAS:0-00-0 MolWeight:296 RetIndex:2044
CompName:Methacrylic acid, pentadecyl ester

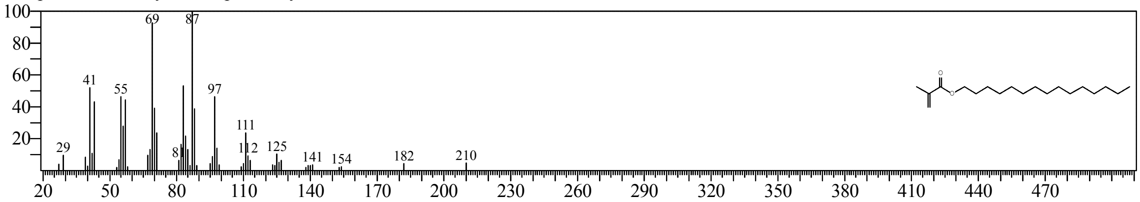

Hit#:2 Entry:89149 Library:NIST08.LIB
SI:92 Formula:C17H32O2 CAS:0-00-0 MolWeight:268 RetIndex:1846
CompName:Methacrylic acid, tridecyl ester

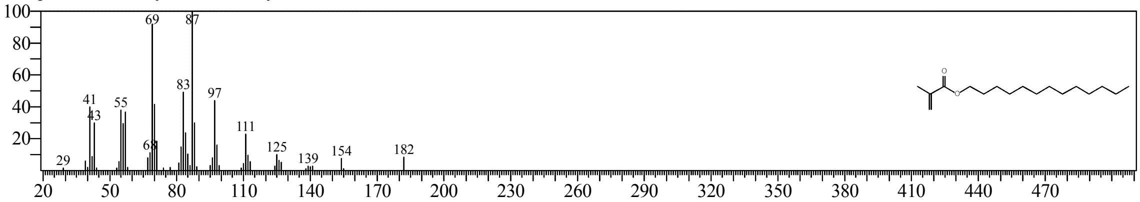

Hit#:3 Entry:79189 Library:NIST08.LIB
SI:92 Formula:C16H30O2 CAS:0-00-0 MolWeight:254 RetIndex:1746
CompName:Methacrylic acid, dodecyl ester

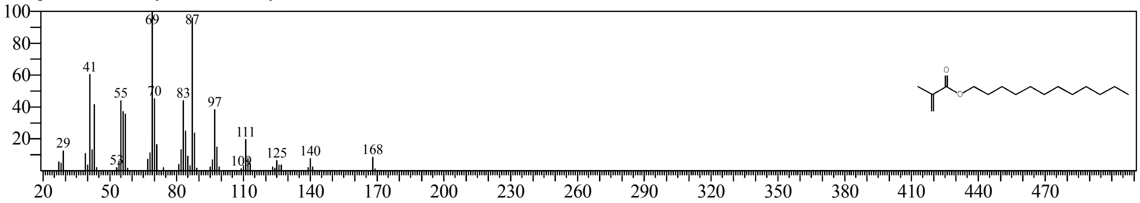

Hit#:4 Entry:99188 Library:NIST08.LIB
SI:92 Formula:C18H34O2 CAS:0-00-0 MolWeight:282 RetIndex:1945
CompName:Methacrylic acid, tetradecyl ester

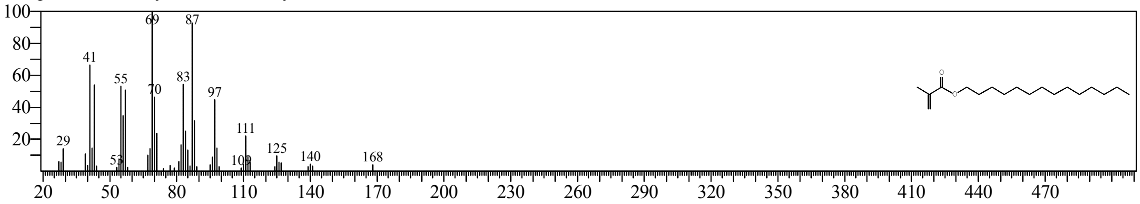

Hit#:5 Entry:119289 Library:NIST08.LIB
SI:92 Formula:C20H38O2 CAS:0-00-0 MolWeight:310 RetIndex:2144
CompName:Methacrylic acid, hexadecyl ester

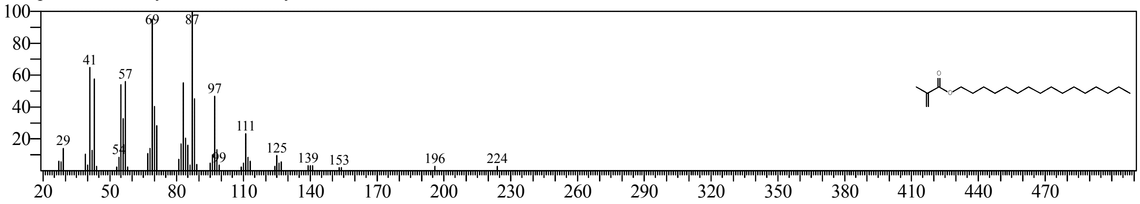

<< Target >>
Line#:13   R.Time:37.500(Scan#:6801) MassPeaks:252
RawMode:Single 37.500(6801) BasePeak:81.05(4578)
BG Mode:37.435(6788) Group 1 - Event 1

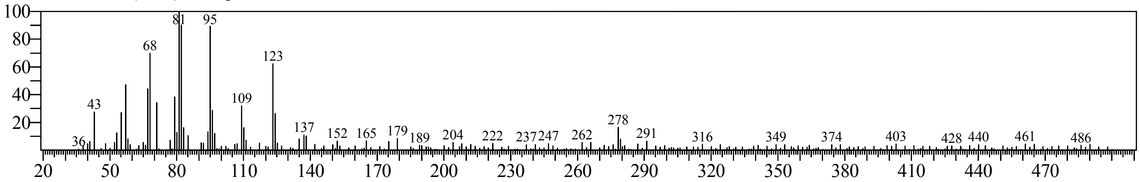

Hit#:1 Entry:109355 Library:NIST08.LIB
SI:79 Formula:C20H40O CAS:102608-53-7 MolWeight:296 RetIndex:2045
CompName:3,7,11,15-Tetramethyl-2-hexadecen-1-ol $$ (2E)-3,7,11,15-Tetramethyl-2-hexadecen-1-ol # $$

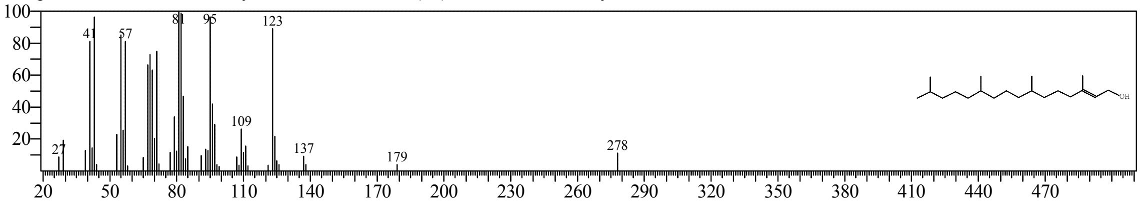

Hit#:2 Entry:908 Library:FFNSC1.3.lib
SI:77 Formula:C20 H38 CAS:504-96-1 MolWeight:278 RetIndex:1836
CompName:Neophytadiene

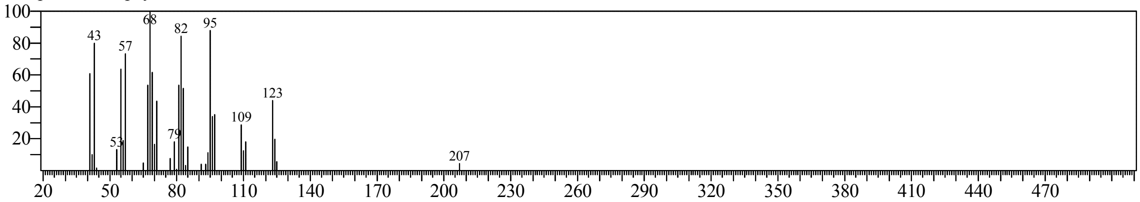

Hit#:3 Entry:96436 Library:NIST08.LIB
SI:77 Formula:C20H38 CAS:61886-66-6 MolWeight:278 RetIndex:2027
CompName:3-Eicosyne $$ 3-Icosyne # $$

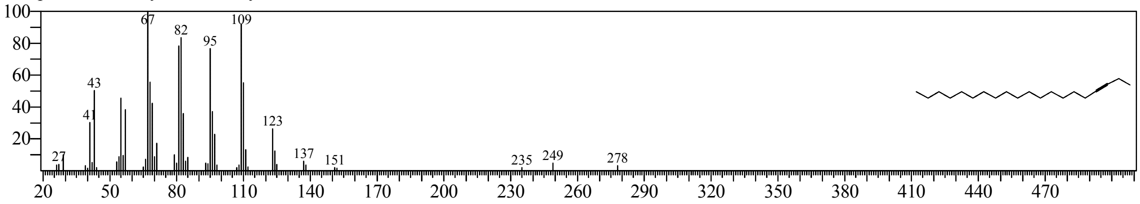

Hit#:4 Entry:86382 Library:NIST08.LIB
SI:76 Formula:C19H36 CAS:35354-38-2 MolWeight:264 RetIndex:1863
CompName:7-Octadecyne, 2-methyl- $$ 2-Methyl-7-octadecyne $$

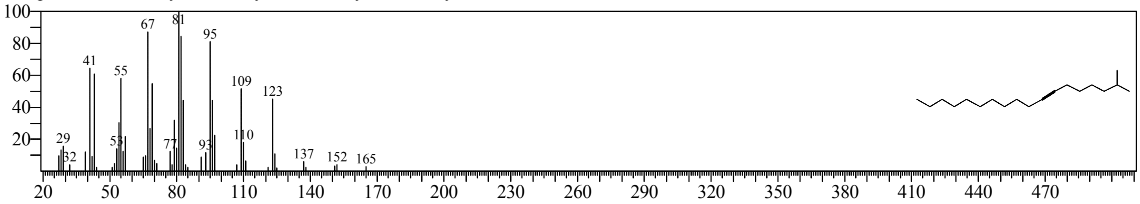

Hit#:5 Entry:59 Library:FFNSC1.3.lib
SI:76 Formula:C15 H28 O2 CAS:7540-53-6 MolWeight:240 RetIndex:1624
CompName:Citronellyl valerate

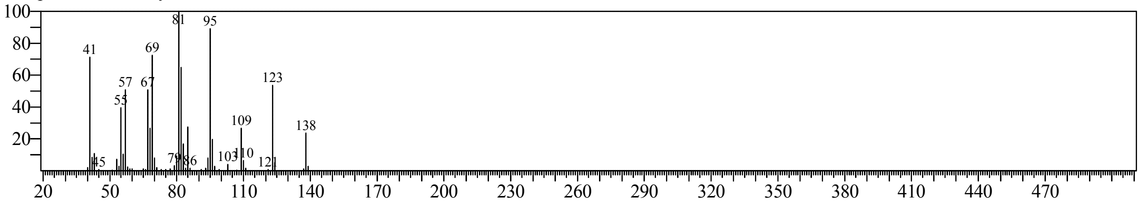

<< Target >>
Line#:14   R.Time:39.305(Scan#:7162) MassPeaks:297
RawMode:Single 39.305(7162) BasePeak:74.00(12031)
BG Mode:39.240(7149) Group 1 - Event 1

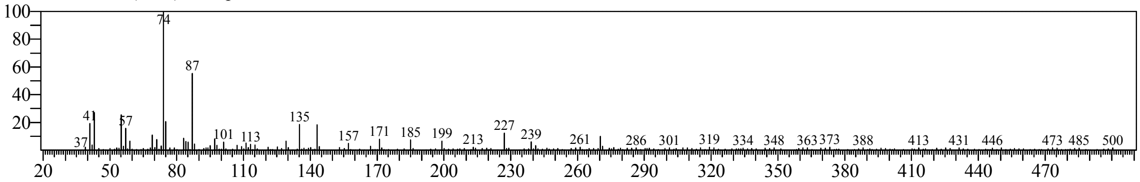

Hit#:1 Entry:90719 Library:NIST08.LIB
SI:90 Formula:C17H34O2 CAS:112-39-0 MolWeight:270 RetIndex:1878
CompName:Hexadecanoic acid, methyl ester $$ Palmitic acid, methyl ester $$ n-Hexadecanoic acid methyl ester $$ Metholene 2216 $$ Methyl hexadecano

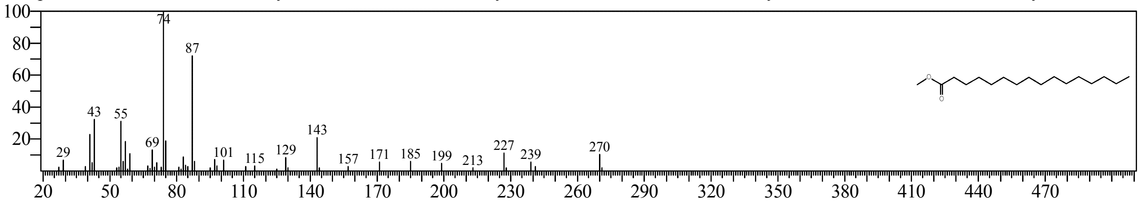

Hit#:2 Entry:22757 Library:NIST08s.LIB
SI:86 Formula:C17H34O2 CAS:5129-60-2 MolWeight:270 RetIndex:1814
CompName:Pentadecanoic acid, 14-methyl-, methyl ester $$ Methyl 14-methylpentadecanoate # $$

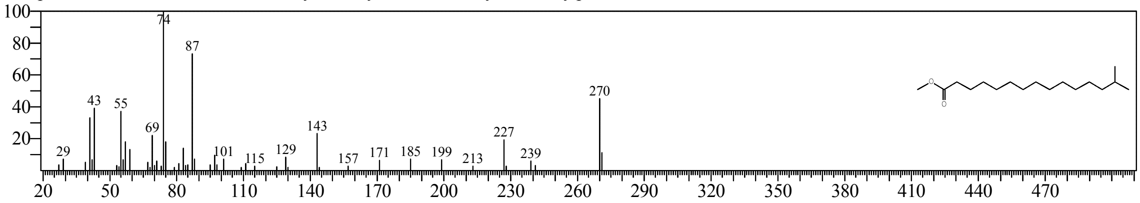

Hit#:3 Entry:90722 Library:NIST08.LIB
SI:86 Formula:C17H34O2 CAS:5487-50-3 MolWeight:270 RetIndex:1814
CompName:Pentadecanoic acid, 13-methyl-, methyl ester $$ Methyl 13-methylpentadecanoate # $$

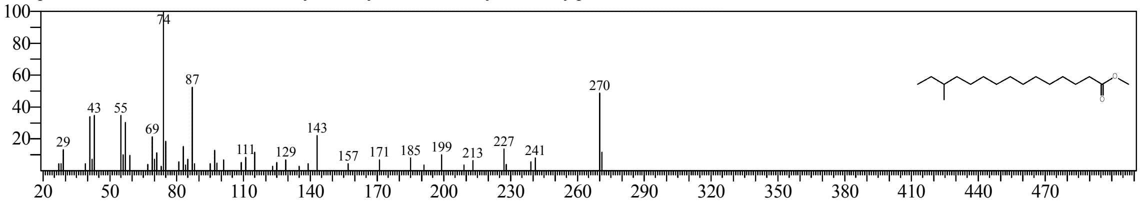

Hit#:4 Entry:80758 Library:NIST08.LIB
SI:86 Formula:C16H32O2 CAS:7132-64-1 MolWeight:256 RetIndex:1779
CompName:Pentadecanoic acid, methyl ester $$ Methyl n-pentadecanoate $$ Methyl pentadecanoate $$ n-Pentadecanoic acid methyl ester $$

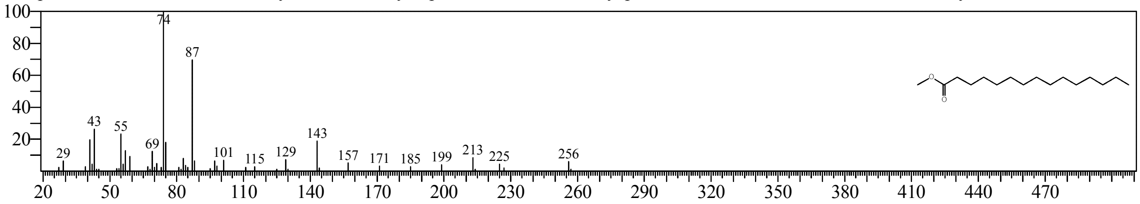

Hit#:5 Entry:120734 Library:NIST08.LIB
SI:85 Formula:C20H40O2 CAS:1731-94-8 MolWeight:312 RetIndex:2177
CompName:Nonadecanoic acid, methyl ester $$ Methyl nonadecanoate $$ n-Nonadecanoic acid methyl ester $$

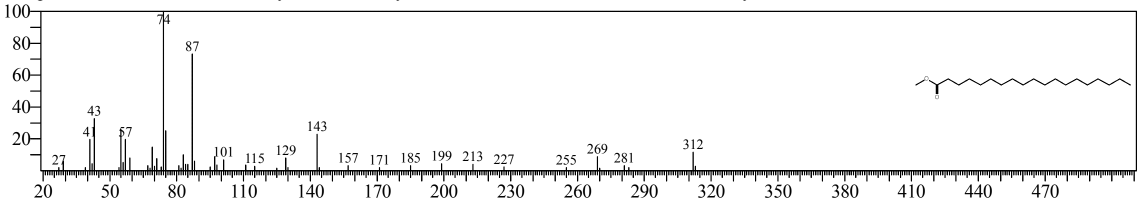

<< Target >>
Line#:15 R.Time:39.495(Scan#:7200) MassPeaks:305
RawMode:Single 39.495(7200) BasePeak:277.20(7295)
BG Mode:39.395(7180) Group 1 - Event 1

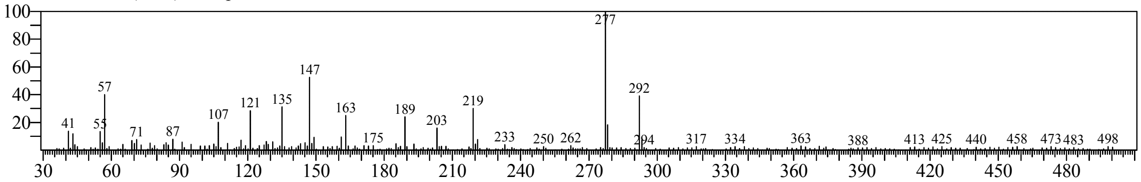

Hit#:1 Entry:106456 Library:NIST08.LIB
SI:74 Formula:C18H28O3 CAS:6386-38-5 MolWeight:292 RetIndex:2134
CompName:Benzenepropanoic acid, 3,5-bis(1,1-dimethylethyl)-4-hydroxy-, methyl ester $$ Methyl 3-(3,5-di-tert-butyl-4-hydroxyphenyl)propionate $$ Metl

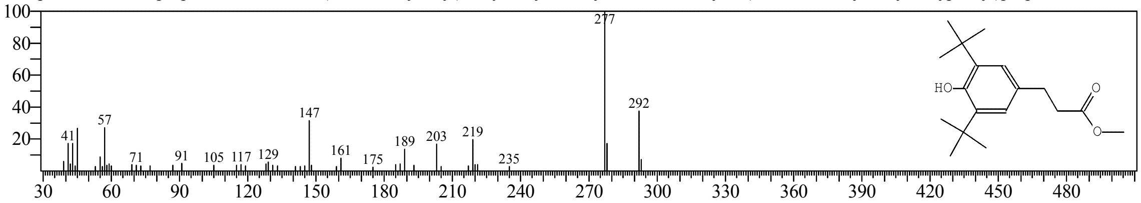

<< Target >>
Line#:16 R.Time:40.660(Scan#:7433) MassPeaks:293
RawMode:Single 40.660(7433) BasePeak:73.05(7339)
BG Mode:40.550(7411) Group 1 - Event 1

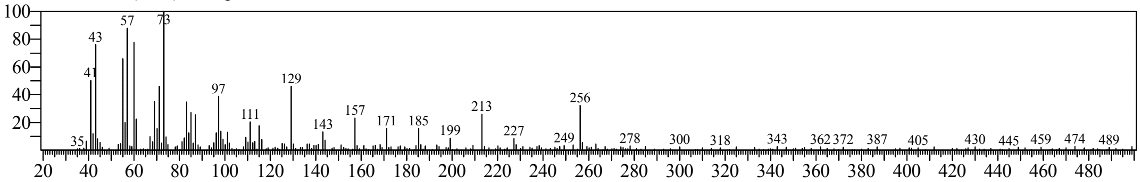

Hit#:1 Entry:189990 Library:NIST08s.LIB
SI:93 Formula:C38H68O8 CAS:28474-90-0 MolWeight:652 RetIndex:4765
CompName:l-(+)-Ascorbic acid 2,6-dihexadecanoate

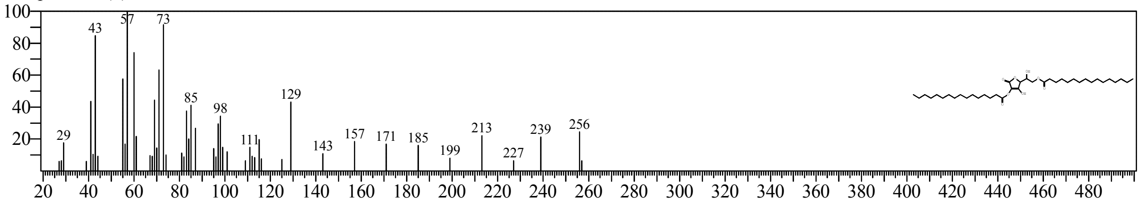

Hit#:2 Entry:21864 Library:NIST08s.LIB
SI:92 Formula:C16H32O2 CAS:57-10-3 MolWeight:256 RetIndex:1968
CompName:n-Hexadecanoic acid $$ Hexadecanoic acid $$ n-Hexadecoic acid $$ Palmitic acid $$ Pentadecanecarboxylic acid $$ 1-Pentadecanecarboxylic

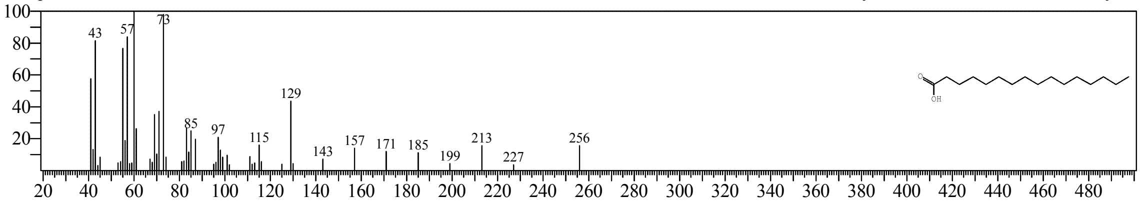

Hit#:3 Entry:20839 Library:NIST08s.LIB
SI:89 Formula:C15H30O2 CAS:1002-84-2 MolWeight:242 RetIndex:1869
CompName:Pentadecanoic acid $$ Pentadecylic acid $$ n-Pentadecanoic acid $$ n-Pentadecylic acid $$

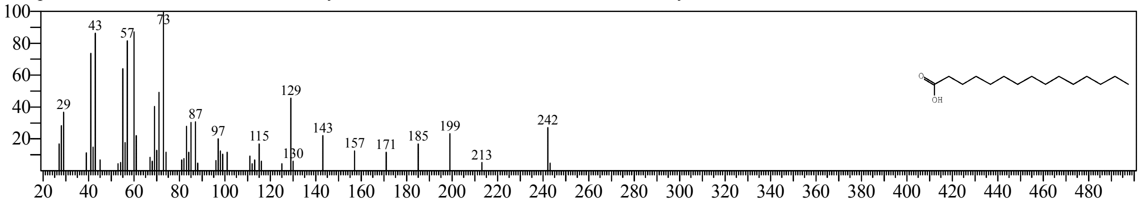

Hit#:4 Entry:23514 Library:NIST08s.LIB
SI:88 Formula:C18H36O2 CAS:57-11-4 MolWeight:284 RetIndex:2167
CompName:Octadecanoic acid $$ Stearic acid $$ n-Octadecanoic acid $$ Humko Industrene R $$ Hydrofol Acid 150 $$ Hystrene S-97 $$ Hystrene T-70 $

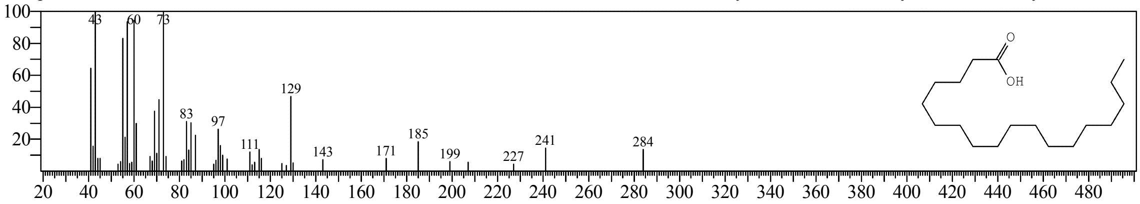

Hit#:5 Entry:120718 Library:NIST08.LIB
SI:87 Formula:C20H40O2 CAS:506-30-9 MolWeight:312 RetIndex:2366
CompName:Eicosanoic acid $$ Arachic acid $$ Arachidic acid $$ Icosanoic acid $$ n-Eicosanoic acid $$ Arachidic acid (synthetic) $$

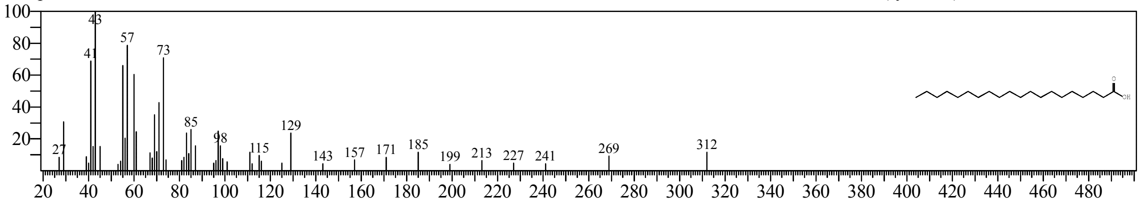

<< Target >>
Line#:17 R.Time:40.815(Scan#:7464) MassPeaks:263
RawMode:Single 40.815(7464) BasePeak:69.05(25832)
BG Mode:40.725(7446) Group 1 - Event 1

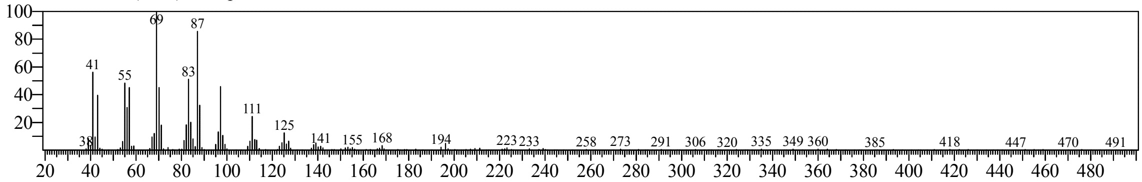

Hit#:1 Entry:109293 Library:NIST08.LIB
SI:96 Formula:C19H36O2 CAS:0-00-0 MolWeight:296 RetIndex:2044
CompName:Methacrylic acid, pentadecyl ester

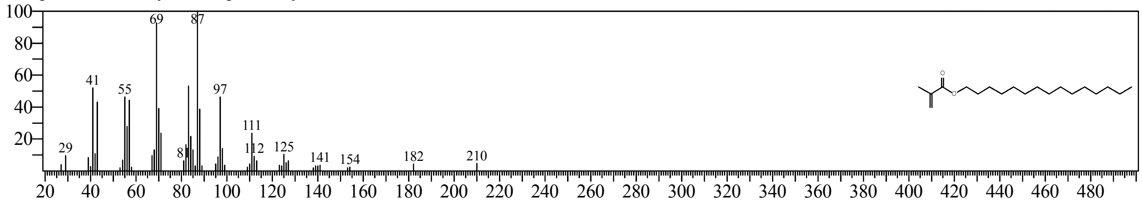

Hit#:2 Entry:99188 Library:NIST08.LIB
SI:95 Formula:C18H34O2 CAS:0-00-0 MolWeight:282 RetIndex:1945
CompName:Methacrylic acid, tetradecyl ester

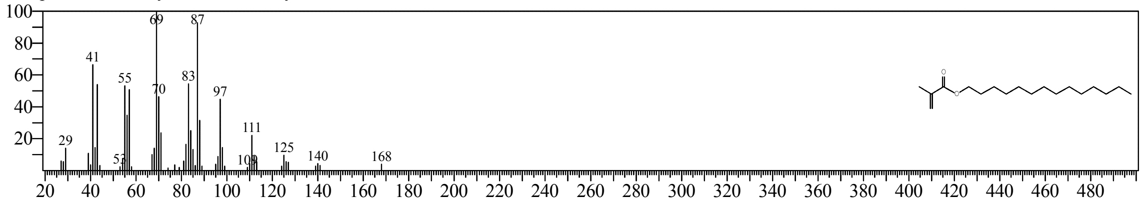

Hit#:3 Entry:79189 Library:NIST08.LIB
SI:94 Formula:C16H30O2 CAS:0-00-0 MolWeight:254 RetIndex:1746
CompName:Methacrylic acid, dodecyl ester

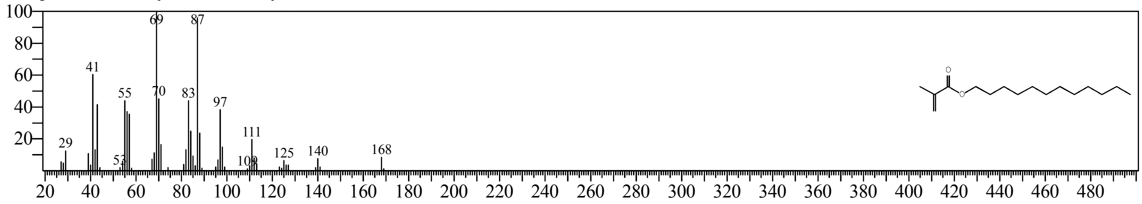

Hit#:4 Entry:119289 Library:NIST08.LIB
SI:94 Formula:C20H38O2 CAS:0-00-0 MolWeight:310 RetIndex:2144
CompName:Methacrylic acid, hexadecyl ester

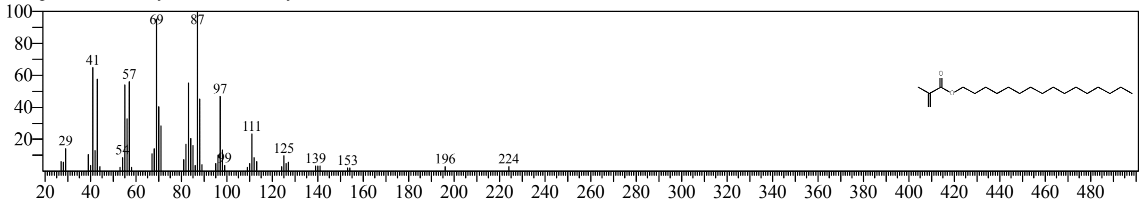

Hit#:5 Entry:89149 Library:NIST08.LIB
SI:94 Formula:C17H32O2 CAS:0-00-0 MolWeight:268 RetIndex:1846
CompName:Methacrylic acid, tridecyl ester

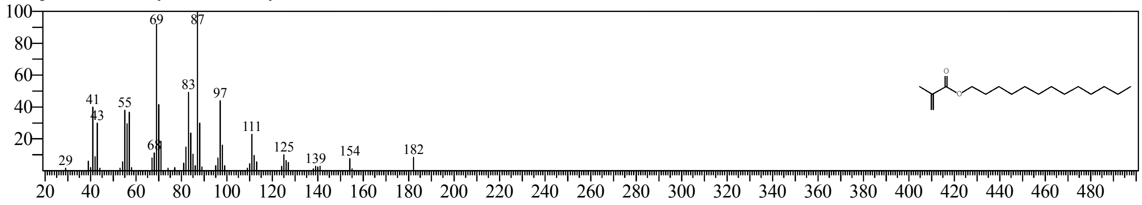

<< Target >>
Line#:18   R.Time:41.235(Scan#:7548) MassPeaks:236
RawMode:Single 41.235(7548) BasePeak:97.10(69120)
BG Mode:41.140(7529) Group 1 - Event 1

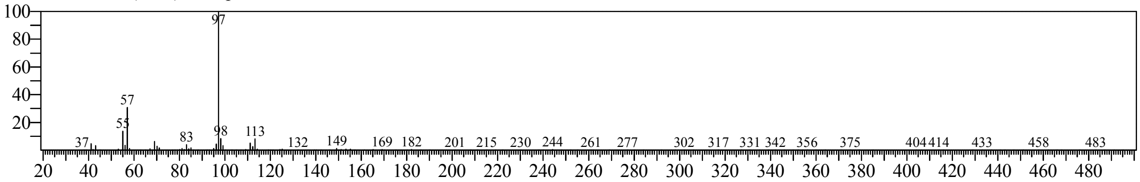

Hit#:1 Entry:163506 Library:NIST08.LIB
SI:85 Formula:C22H44O3S CAS:0-00-0 MolWeight:388 RetIndex:2895
CompName:Sulfurous acid, cyclohexylmethyl pentadecyl ester

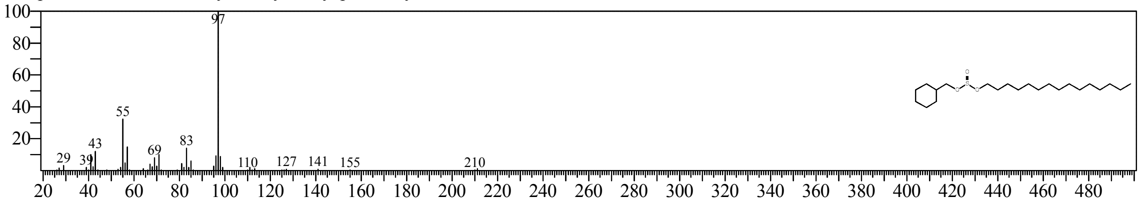

Hit#:2 Entry:175422 Library:NIST08.LIB
SI:85 Formula:C25H50O3S CAS:0-00-0 MolWeight:430 RetIndex:3193
CompName:Sulfurous acid, cyclohexylmethyl octadecyl ester

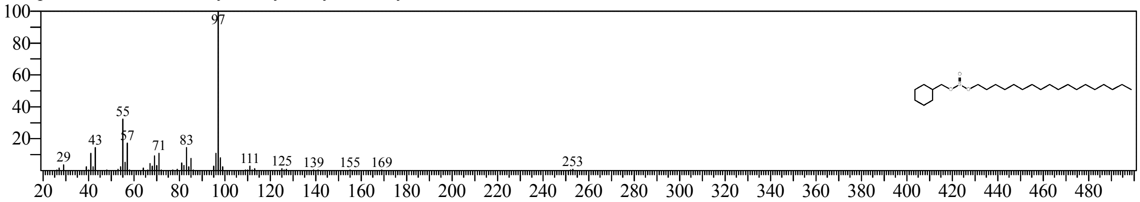

Hit#:3 Entry:157624 Library:NIST08.LIB
SI:85 Formula:C21H42O3S CAS:0-00-0 MolWeight:374 RetIndex:2795
CompName:Sulfurous acid, cyclohexylmethyl tetradecyl ester

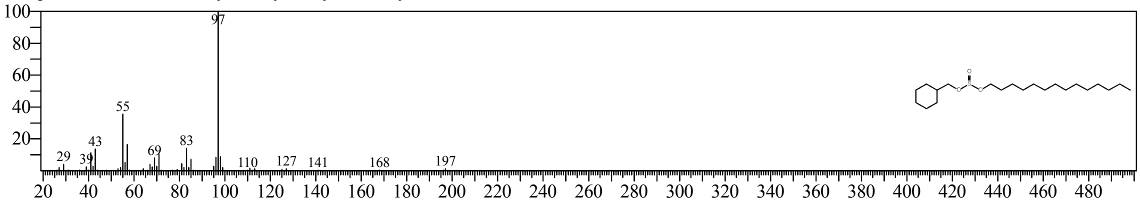

Hit#:4 Entry:150441 Library:NIST08.LIB
SI:85 Formula:C20H40O3S CAS:0-00-0 MolWeight:360 RetIndex:2696
CompName:Sulfurous acid, cyclohexylmethyl tridecyl ester

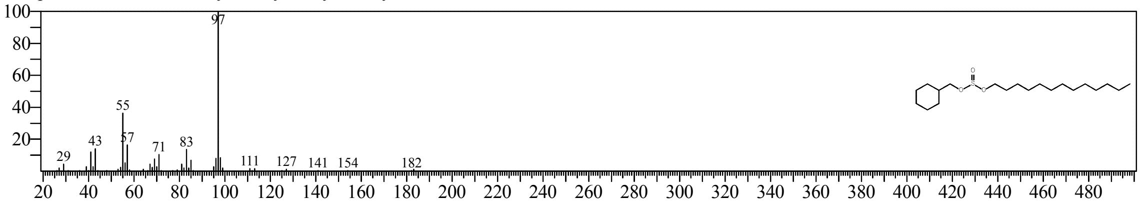

Hit#:5 Entry:172226 Library:NIST08.LIB
SI:85 Formula:C24H48O3S CAS:0-00-0 MolWeight:416 RetIndex:3094
CompName:Sulfurous acid, cyclohexylmethyl heptadecyl ester

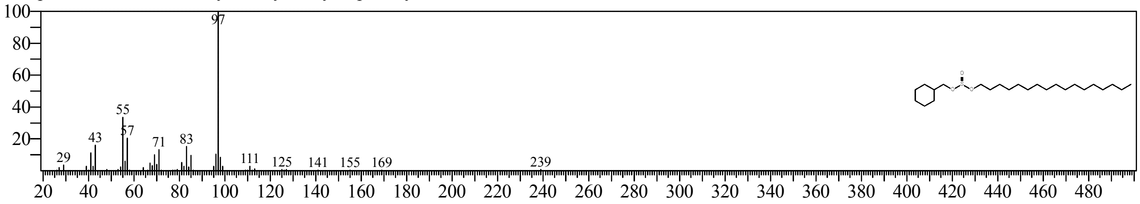

<< Target >>
Line#:19 R.Time:42.620(Scan#:7825) MassPeaks:291
RawMode:Single 42.620(7825) BasePeak:57.05(38012)
BG Mode:42.545(7810) Group 1 - Event 1

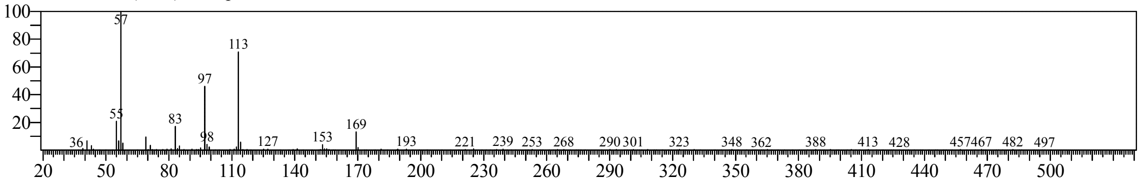

Hit#:1 Entry:58935 Library:NIST08.LIB
SI:80 Formula:C16H32 CAS:15796-04-0 MolWeight:224 RetIndex:1325
CompName:2,4,4,6,6,8,8-Heptamethyl-1-nonene

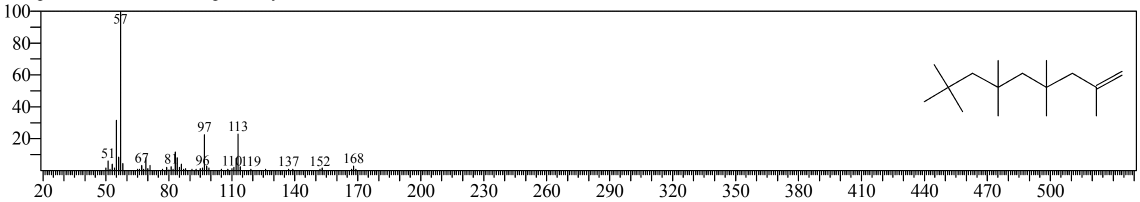

Hit#:2 Entry:188758 Library:NIST08.LIB
SI:78 Formula:C42H86 CAS:55470-97-8 MolWeight:590 RetIndex:3473
CompName:Octadecane, 2,2,4,15,17,17-hexamethyl-7,12-bis(3,5,5-trimethylhexyl)- $$ 2,2,4,15,17,17-Hexamethyl-7,12-di(3',5',5'-trimethylhexyl)octadecan

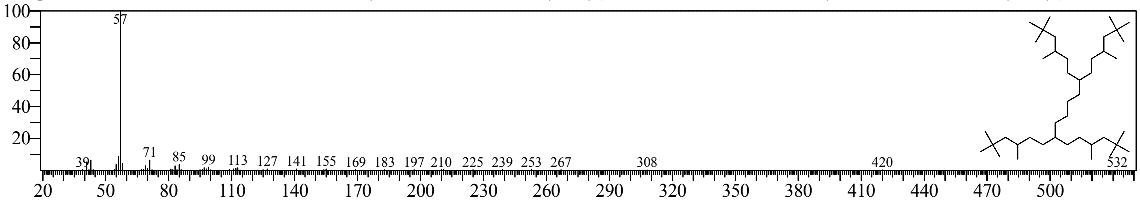

Hit#:3 Entry:19532 Library:NIST08s.LIB
SI:77 Formula:C16H34 CAS:4390-04-9 MolWeight:226 RetIndex:1294
CompName:Nonane, 2,2,4,4,6,8,8-heptamethyl- $$ 2,2,4,4,6,8,8-Heptamethylnonane $$ HMN $$ Permethyl 101A $$

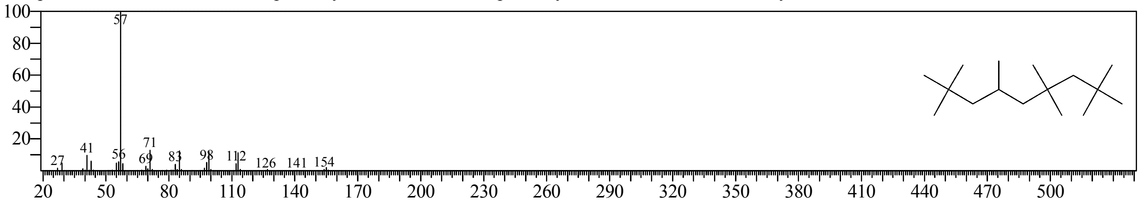

Hit#:4 Entry:165157 Library:NIST08.LIB
SI:76 Formula:C28H56 CAS:55255-73-7 MolWeight:392 RetIndex:2344
CompName:6-Tridecene, 2,2,4,10,12,12-hexamethyl-7-(3,5,5-trimethylhexyl)- $$ 2,2,4,10,12,12-Hexamethyl-7-(3,5,5-trimethylhexyl)-6-tridecene $$

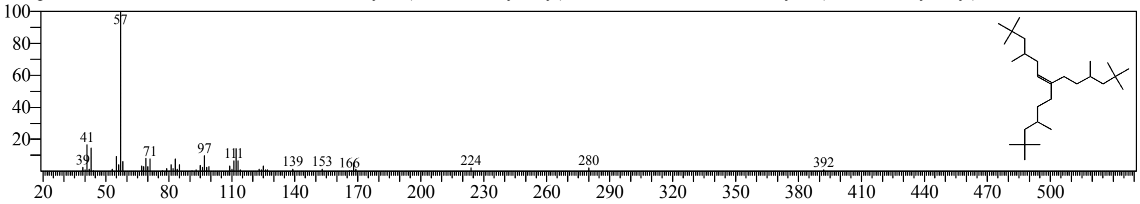

Hit#:5 Entry:165852 Library:NIST08.LIB
SI:76 Formula:C28H58 CAS:3035-75-4 MolWeight:394 RetIndex:2295
CompName:Tridecane, 2,2,4,10,12,12-hexamethyl-7-(3,5,5-trimethylhexyl)- $$ 2,2,4,10,12,12-Hexamethyl-7-(3,5,5-trimethylhexyl)tridecane $$

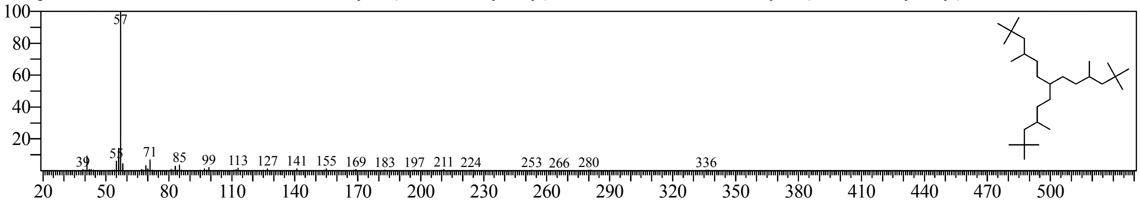

<< Target >>
Line#:20 R.Time:44.120(Scan#:8125) MassPeaks:291
RawMode:Single 44.120(8125) BasePeak:57.05(14404)
BG Mode:44.040(8109) Group 1 - Event 1

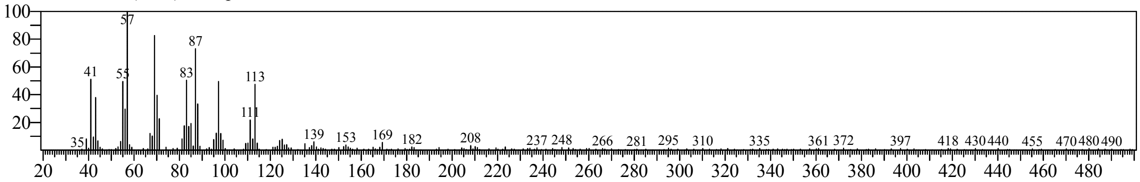

Hit#:1 Entry:109293 Library:NIST08.LIB
SI:92 Formula:C19H36O2 CAS:0-00-0 MolWeight:296 RetIndex:2044
CompName:Methacrylic acid, pentadecyl ester

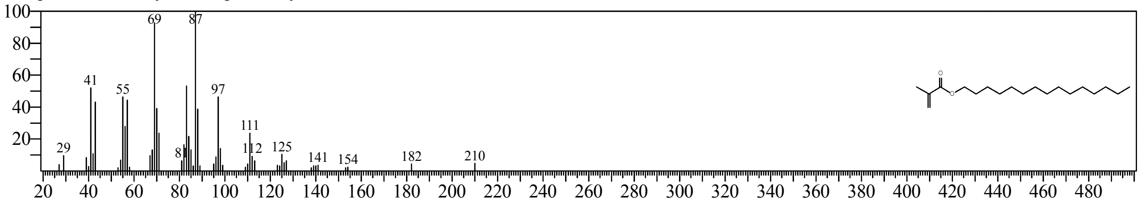

Hit#:2 Entry:146279 Library:NIST08.LIB
SI:92 Formula:C23H44O2 CAS:0-00-0 MolWeight:352 RetIndex:2442
CompName:Methacrylic acid, nonadecyl ester

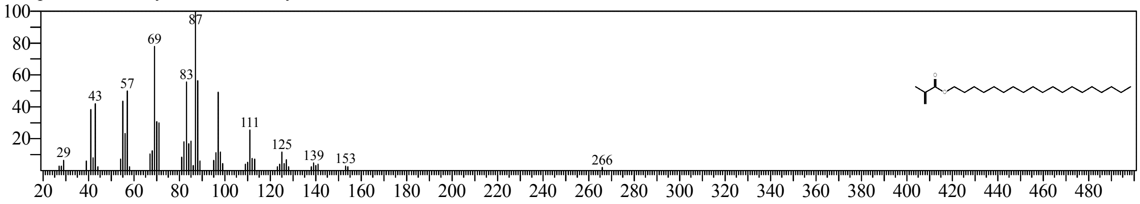

Hit#:3 Entry:128769 Library:NIST08.LIB
SI:91 Formula:C21H40O2 CAS:0-00-0 MolWeight:324 RetIndex:2243
CompName:Methacrylic acid, heptadecyl ester

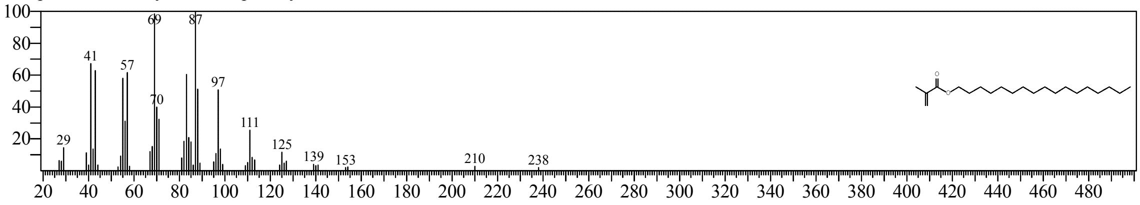

Hit#:4 Entry:119289 Library:NIST08.LIB
SI:91 Formula:C20H38O2 CAS:0-00-0 MolWeight:310 RetIndex:2144
CompName:Methacrylic acid, hexadecyl ester

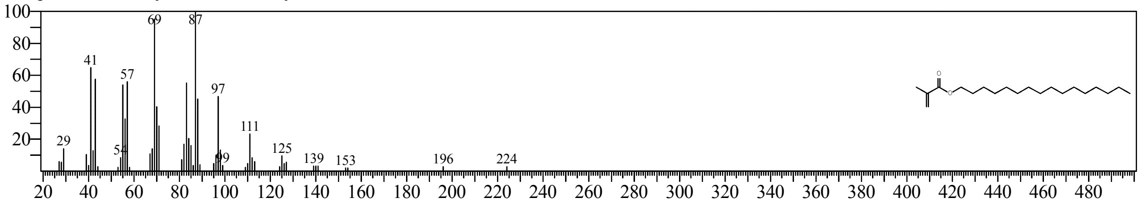

Hit#:5 Entry:99188 Library:NIST08.LIB
SI:90 Formula:C18H34O2 CAS:0-00-0 MolWeight:282 RetIndex:1945
CompName:Methacrylic acid, tetradecyl ester

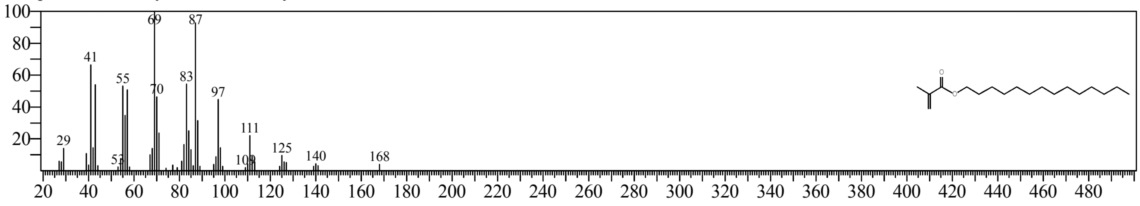

<< Target >>
Line#:21 R.Time:44.560(Scan#:8213) MassPeaks:269
RawMode:Single 44.560(8213) BasePeak:83.10(6856)
BG Mode:44.485(8198) Group 1 - Event 1

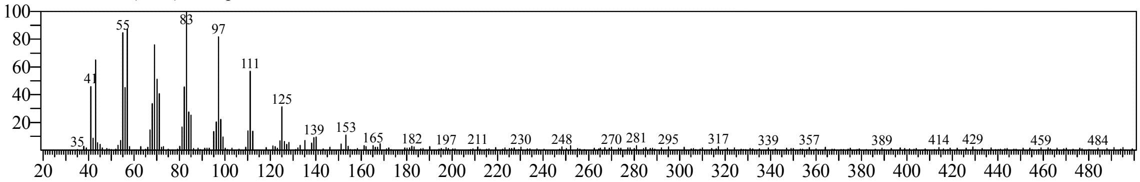

Hit#:1 Entry:25469 Library:NIST08s.LIB
SI:93 Formula:C22H46O CAS:661-19-8 MolWeight:326 RetIndex:2451
CompName:Behenic alcohol $$ 1-Docosanol $$ Docosyl alcohol $$ Docosanol-(1) $$ Cachalot BE-22 $$ Dehydag wax 22 (lanette) $$ Emery 3304 $$ Lo

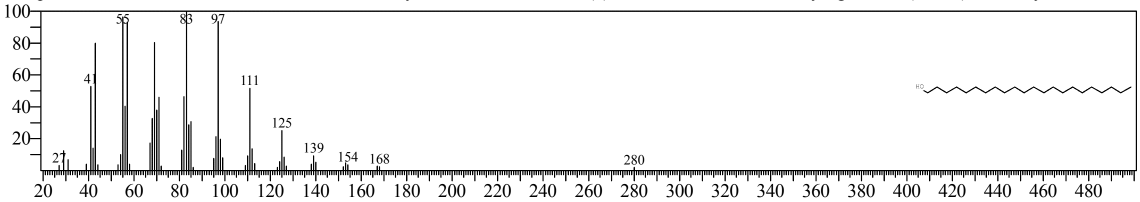

Hit#:2 Entry:100901 Library:NIST08.LIB
SI:93 Formula:C19H40O CAS:1454-84-8 MolWeight:284 RetIndex:2153
CompName:n-Nonadecanol-1 $$ 1-Nonadecanol $$ Nonadecanol $$ Nonadecan-1-ol $$ Nonadecyl alcohol $$

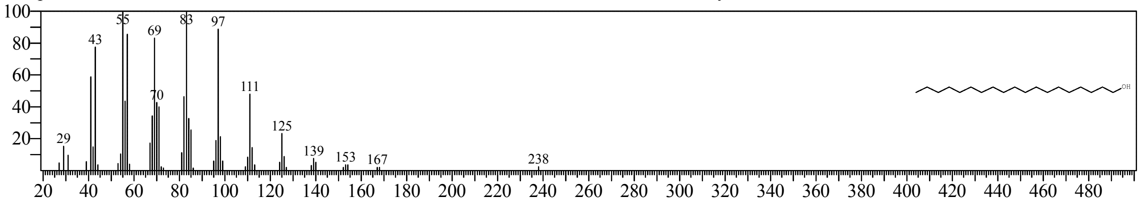

Hit#:3 Entry:147498 Library:NIST08.LIB
SI:93 Formula:C24H50O CAS:506-51-4 MolWeight:354 RetIndex:2650
CompName:n-Tetracosanol-1 $$ Lignoceric alcohol $$ Lignoceryl alcohol $$ 1-Tetracosanol $$ Tetracosan-1-ol $$ Tetracosyl alcohol $$ Lignocerol $$

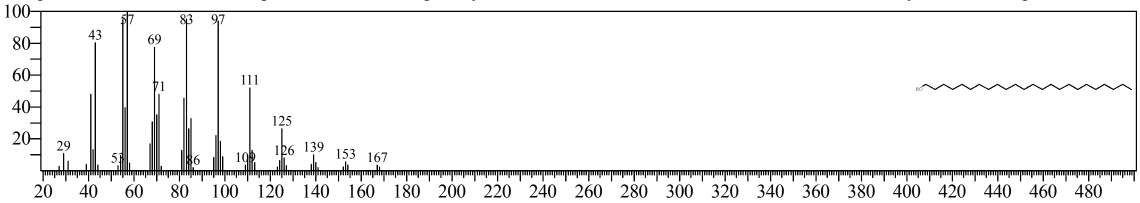

Hit#:4 Entry:166592 Library:NIST08.LIB
SI:93 Formula:C27H56O CAS:2004-39-9 MolWeight:396 RetIndex:2948
CompName:1-Heptacosanol $$ Heptacosan-1-ol $$ Heptacosanol $$

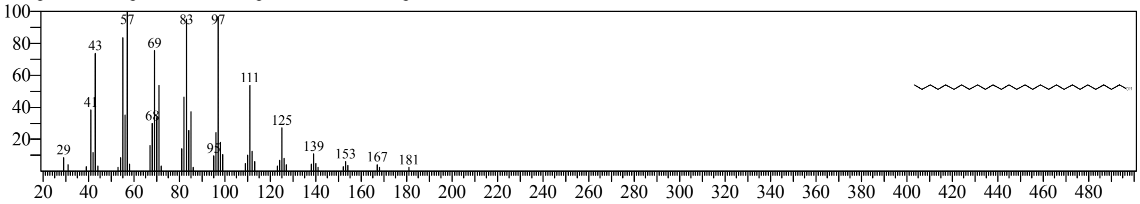

Hit#:5 Entry:120820 Library:NIST08.LIB
SI:92 Formula:C21H44O CAS:15594-90-8 MolWeight:312 RetIndex:2351
CompName:1-Heneicosanol $$ Henicosan-1-ol $$

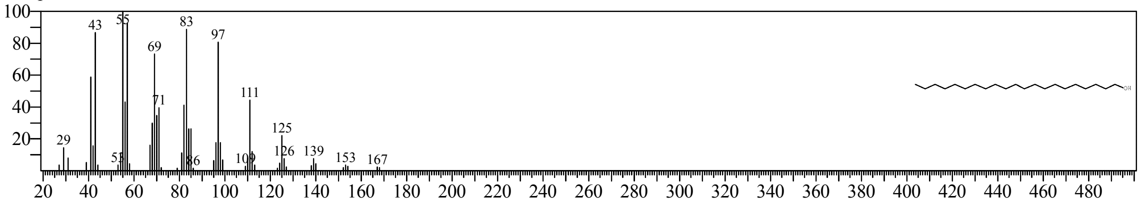

<< Target >>
Line#:22 R.Time:46.515(Scan#:8604) MassPeaks:254
RawMode:Single 46.515(8604) BasePeak:57.05(6037)
BG Mode:46.430(8587) Group 1 - Event 1

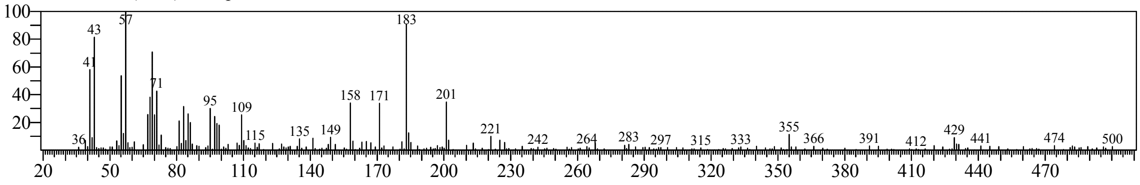

Hit#:1 Entry:19488 Library:NIST08s.LIB
SI:75 Formula:C14H26O2 CAS:2146-71-6 MolWeight:226 RetIndex:1570
CompName:Dodecanoic acid, ethenyl ester $$ Lauric acid, vinyl ester $$ Vinyl laurate $$ Vinyl dodecanoate $$

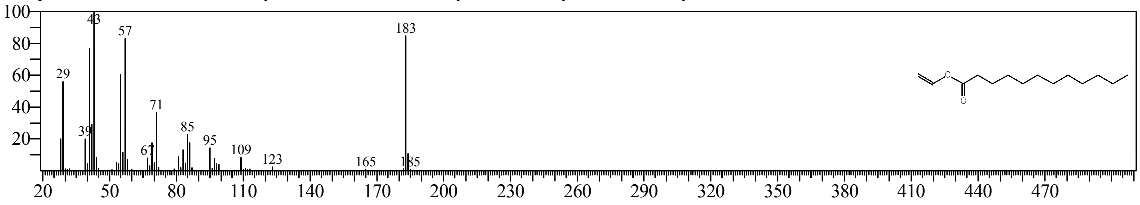

Hit#:2 Entry:126671 Library:NIST08.LIB
SI:75 Formula:C18H27NO4 CAS:1956-11-2 MolWeight:321 RetIndex:2450
CompName:4-Nitrophenyl laurate $$ Dodecanoic acid, 4-nitrophenyl ester $$

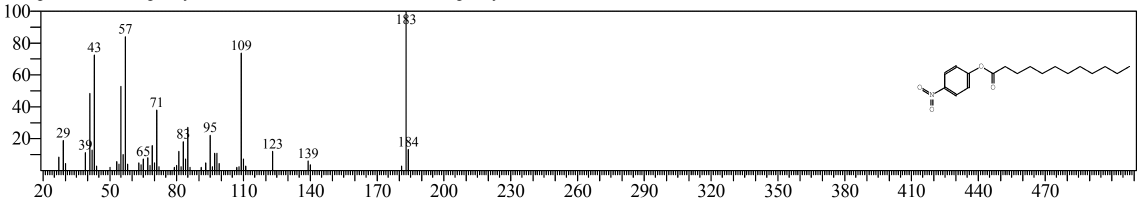

Hit#:3 Entry:120719 Library:NIST08.LIB
SI:73 Formula:C20H40O2 CAS:84713-06-4 MolWeight:312 RetIndex:2112
CompName:Dodecanoic acid, isooctyl ester $$ 1-Propylpentyl laurate # $$

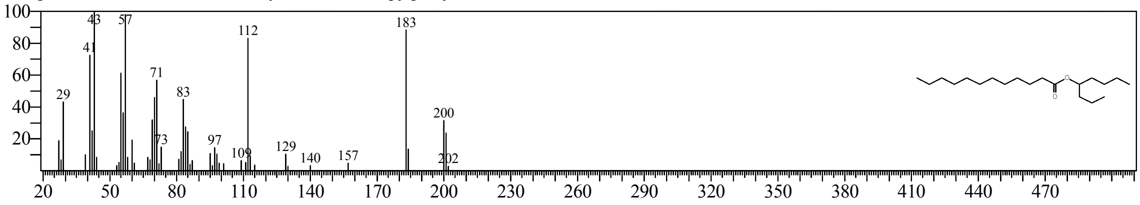

Hit#:4 Entry:167186 Library:NIST08.LIB
SI:72 Formula:C24H46O4 CAS:105-74-8 MolWeight:398 RetIndex:2741
CompName:Lauroyl peroxide $$ di-Lauroyl peroxide $$ Peroxide, bis(1-oxododecyl) $$ Alperox C $$ Dodecanoyl peroxide $$ Laurydol $$ LYP 97 $$ Per

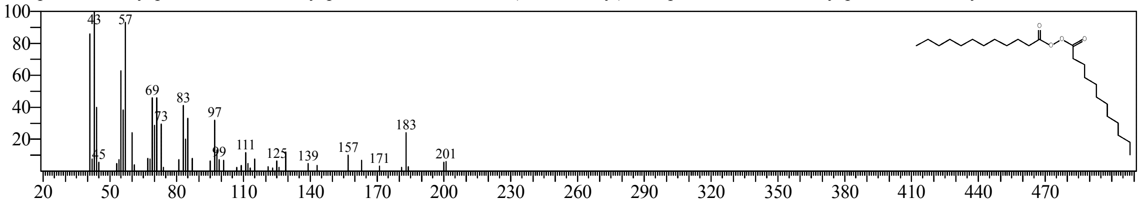

Hit#:5 Entry:99255 Library:NIST08.LIB
SI:72 Formula:C19H38O CAS:29804-22-6 MolWeight:282 RetIndex:1898
CompName:Oxirane, 2-decyl-3-(5-methylhexyl)-, cis- $$.+/-.;-cis-7,8-Epoxy-2-methyloctadecane $$ 2-Decyl-3-(5-methylhexyl)oxirane # $$

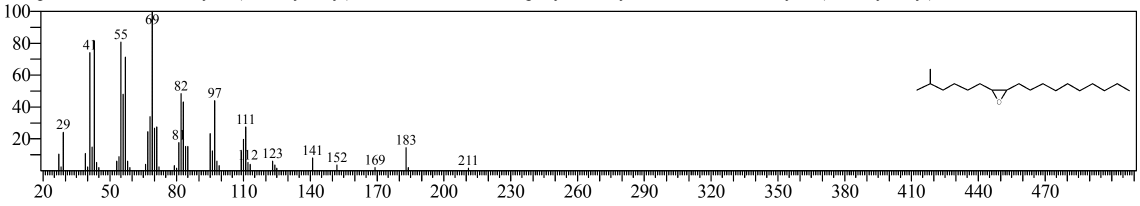

<< Target >>
Line#:23   R.Time:47.275(Scan#:8756) MassPeaks:314
RawMode:Single 47.275(8756) BasePeak:69.05(15716)
BG Mode:47.215(8744) Group 1 - Event 1

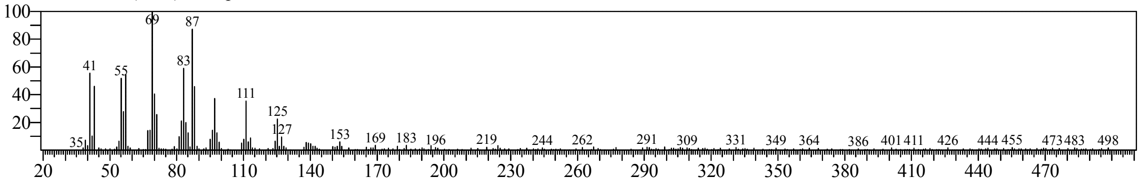

Hit#:1 Entry:119289 Library:NIST08.LIB
SI:94 Formula:C20H38O2 CAS:0-00-0 MolWeight:310 RetIndex:2144
CompName:Methacrylic acid, hexadecyl ester

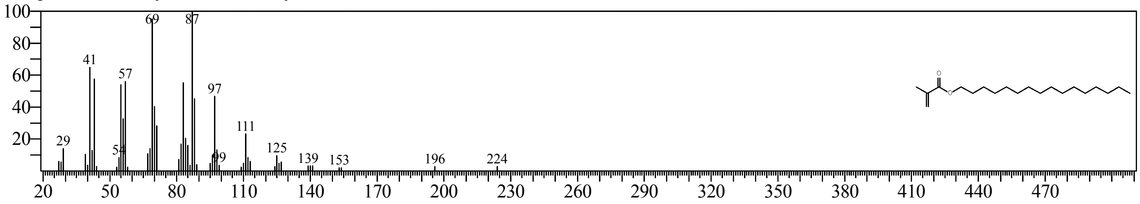

Hit#:2 Entry:128769 Library:NIST08.LIB
SI:93 Formula:C21H40O2 CAS:0-00-0 MolWeight:324 RetIndex:2243
CompName:Methacrylic acid, heptadecyl ester

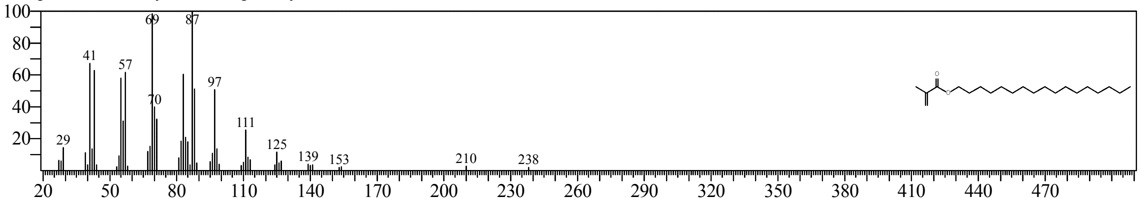

Hit#:3 Entry:109293 Library:NIST08.LIB
SI:93 Formula:C19H36O2 CAS:0-00-0 MolWeight:296 RetIndex:2044
CompName:Methacrylic acid, pentadecyl ester

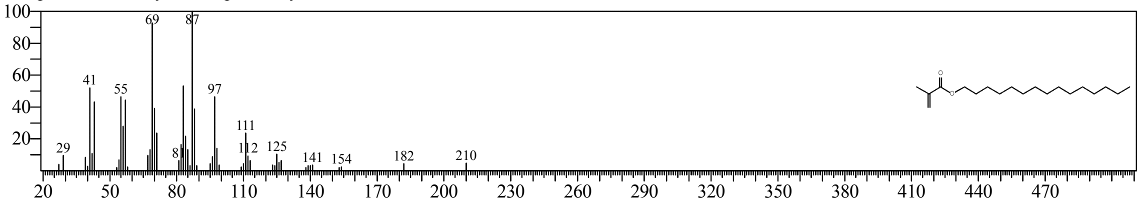

Hit#:4 Entry:146279 Library:NIST08.LIB
SI:93 Formula:C23H44O2 CAS:0-00-0 MolWeight:352 RetIndex:2442
CompName:Methacrylic acid, nonadecyl ester

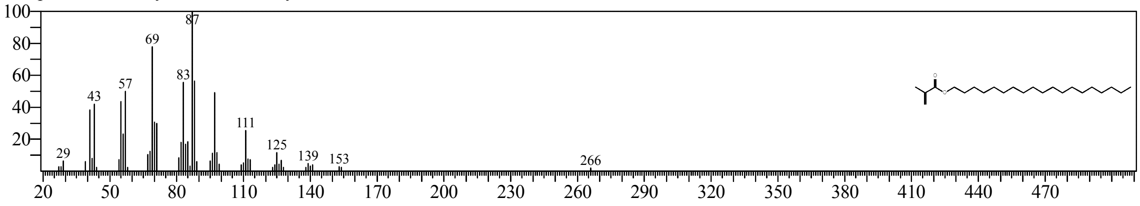

Hit#:5 Entry:99188 Library:NIST08.LIB
SI:92 Formula:C18H34O2 CAS:0-00-0 MolWeight:282 RetIndex:1945
CompName:Methacrylic acid, tetradecyl ester

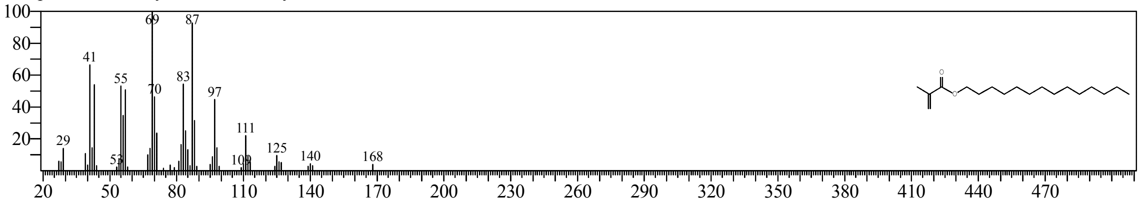

<< Target >>
Line#:24 R.Time:48.890(Scan#:9079) MassPeaks:281
RawMode:Single 48.890(9079) BasePeak:97.10(33443)
BG Mode:48.800(9061) Group 1 - Event 1

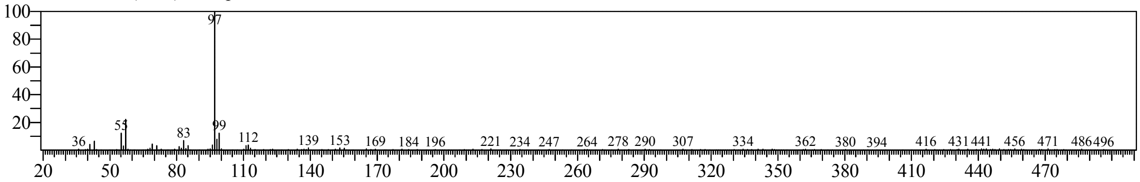

Hit#:1 Entry:163506 Library:NIST08.LIB
SI:83 Formula:C22H44O3S CAS:0-00-0 MolWeight:388 RetIndex:2895
CompName:Sulfurous acid, cyclohexylmethyl pentadecyl ester

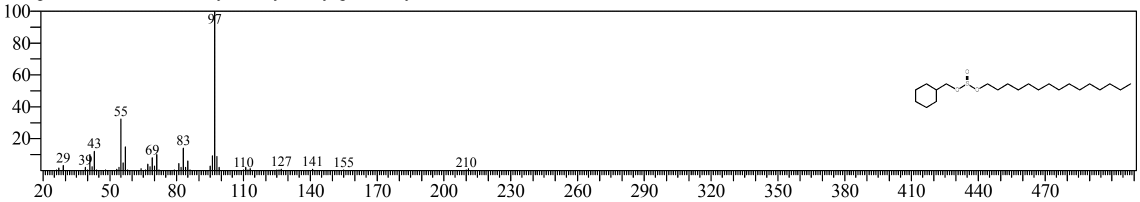

Hit#:2 Entry:175422 Library:NIST08.LIB
SI:83 Formula:C25H50O3S CAS:0-00-0 MolWeight:430 RetIndex:3193
CompName:Sulfurous acid, cyclohexylmethyl octadecyl ester

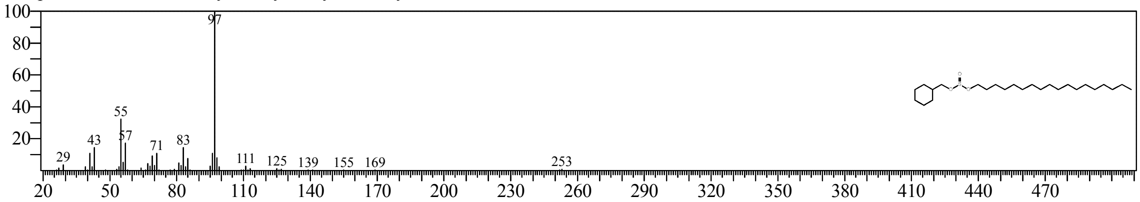

Hit#:3 Entry:150441 Library:NIST08.LIB
SI:82 Formula:C20H40O3S CAS:0-00-0 MolWeight:360 RetIndex:2696
CompName:Sulfurous acid, cyclohexylmethyl tridecyl ester

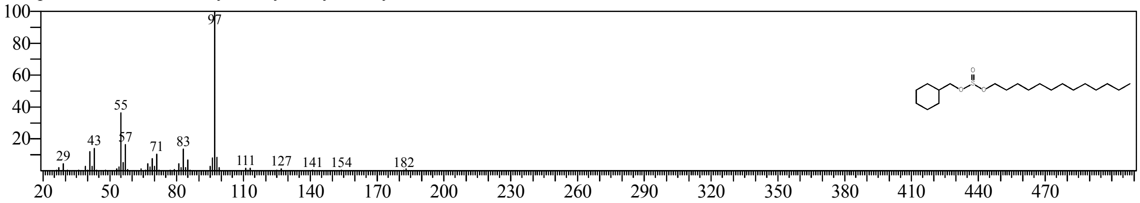

Hit#:4 Entry:157624 Library:NIST08.LIB
SI:82 Formula:C21H42O3S CAS:0-00-0 MolWeight:374 RetIndex:2795
CompName:Sulfurous acid, cyclohexylmethyl tetradecyl ester

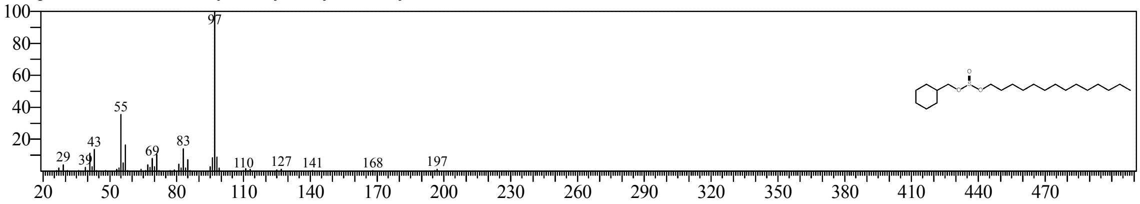

Hit#:5 Entry:142703 Library:NIST08.LIB
SI:82 Formula:C19H38O3S CAS:0-00-0 MolWeight:346 RetIndex:2597
CompName:Sulfurous acid, cyclohexylmethyl dodecyl ester

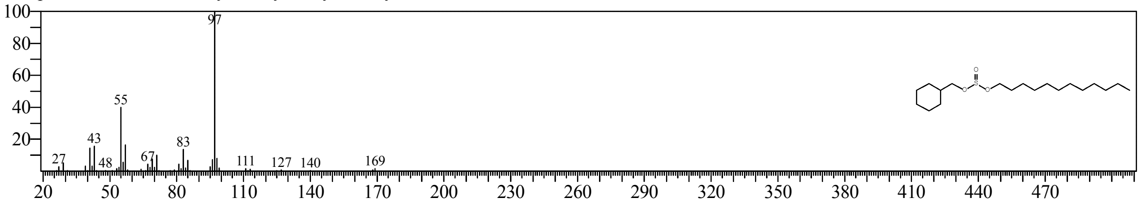

<< Target >>
Line#:25 R.Time:51.530(Scan#:9607) MassPeaks:276
RawMode:Single 51.530(9607) BasePeak:97.10(117664)
BG Mode:51.420(9585) Group 1 - Event 1

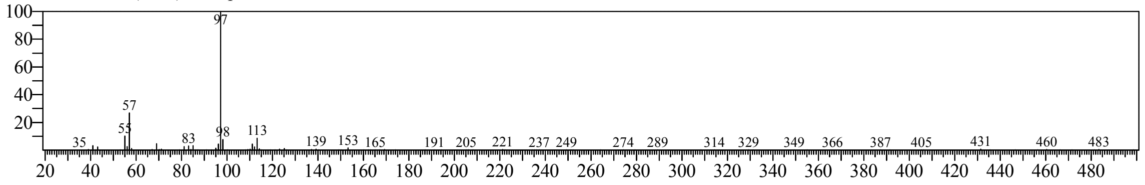

Hit#:1 Entry:125933 Library:NIST08.LIB
SI:83 Formula:C17H37O3P CAS:0-00-0 MolWeight:320 RetIndex:0
CompName:Bis(2-ethylhexyl) methylphosphonate

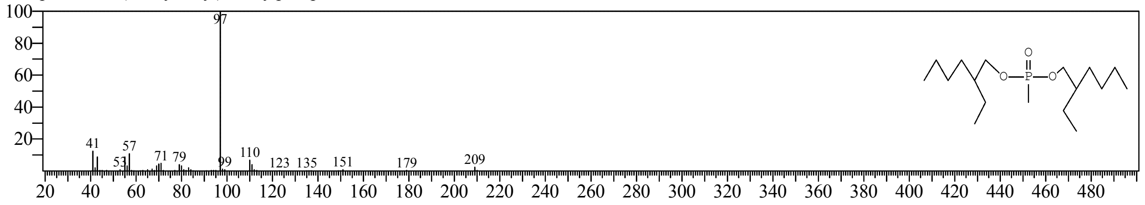

Hit#:2 Entry:163506 Library:NIST08.LIB
SI:83 Formula:C22H44O3S CAS:0-00-0 MolWeight:388 RetIndex:2895
CompName:Sulfurous acid, cyclohexylmethyl pentadecyl ester

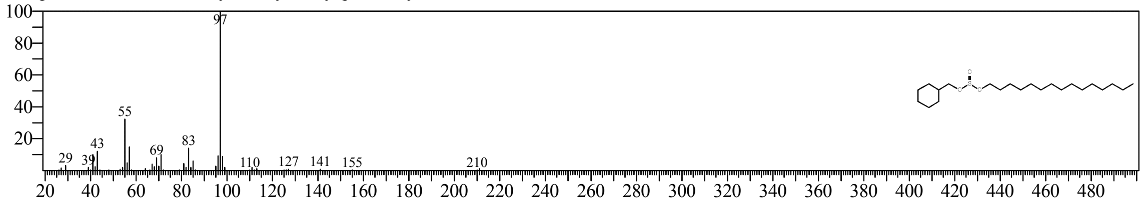

Hit#:3 Entry:175422 Library:NIST08.LIB
SI:82 Formula:C25H50O3S CAS:0-00-0 MolWeight:430 RetIndex:3193
CompName:Sulfurous acid, cyclohexylmethyl octadecyl ester

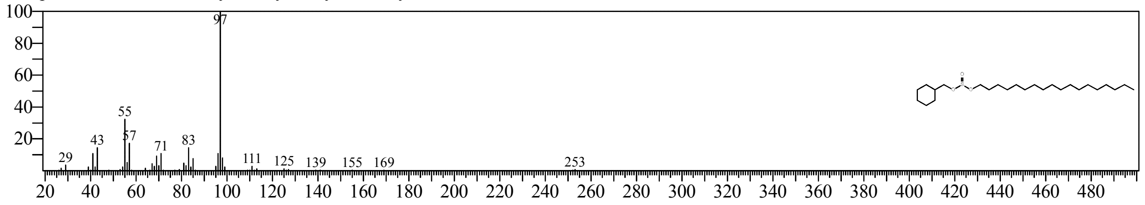

Hit#:4 Entry:157624 Library:NIST08.LIB
SI:82 Formula:C21H42O3S CAS:0-00-0 MolWeight:374 RetIndex:2795
CompName:Sulfurous acid, cyclohexylmethyl tetradecyl ester

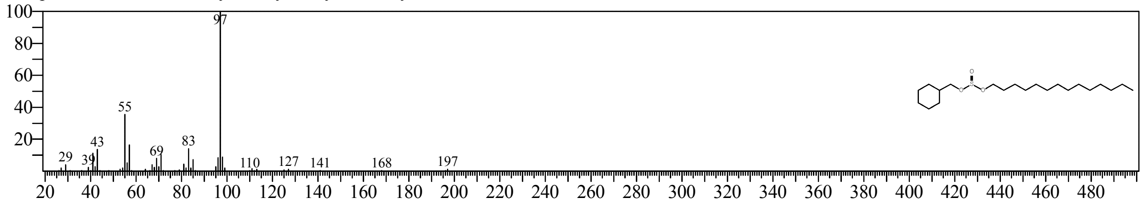

Hit#:5 Entry:142703 Library:NIST08.LIB
SI:82 Formula:C19H38O3S CAS:0-00-0 MolWeight:346 RetIndex:2597
CompName:Sulfurous acid, cyclohexylmethyl dodecyl ester

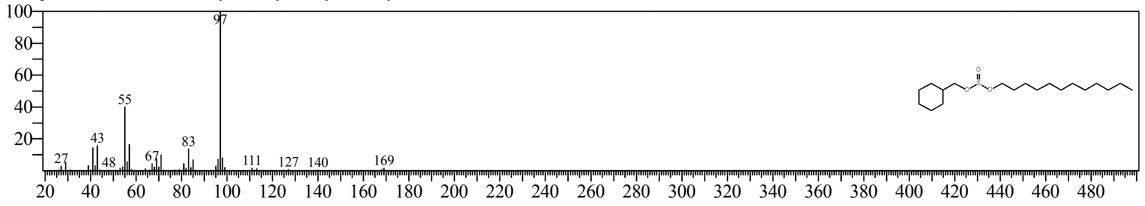

<< Target >>
Line#:26 R.Time:52.750(Scan#:9851) MassPeaks:249
RawMode:Single 52.750(9851) BasePeak:57.05(52059)
BG Mode:52.685(9838) Group 1 - Event 1

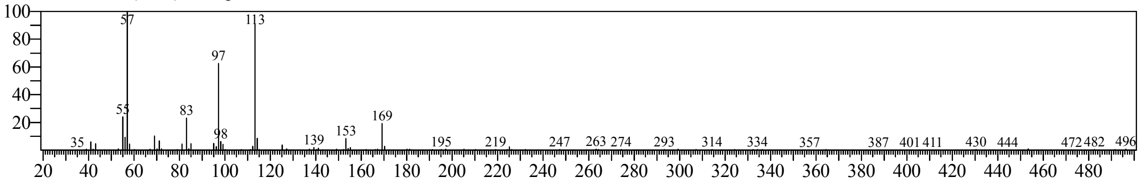

Hit#:1 Entry:58935 Library:NIST08.LIB
SI:77 Formula:C16H32 CAS:15796-04-0 MolWeight:224 RetIndex:1325
CompName:2,4,4,6,6,8,8-Heptamethyl-1-nonene

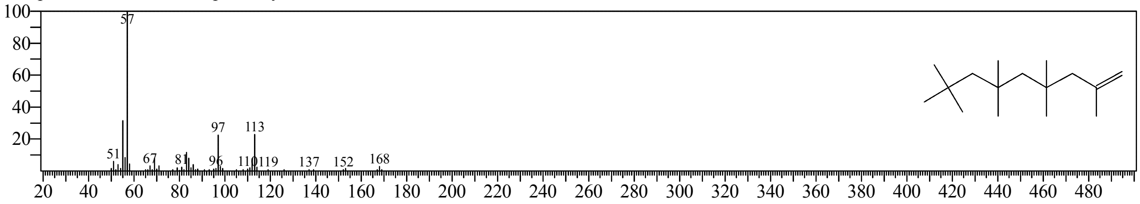

Hit#:2 Entry:41366 Library:NIST08.LIB
SI:75 Formula:C9H20B2O3 CAS:58163-56-7 MolWeight:198 RetIndex:0
CompName:Borinic acid, diethyl-, (2-ethyl-1,3,2-dioxaborolan-4-yl)methyl ester $$ (2-Ethyl-1,3,2-dioxaborolan-4-yl)methyl diethylborinate # $$

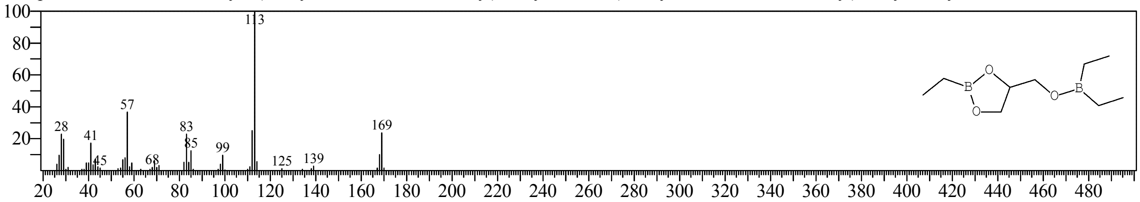

Hit#:3 Entry:172745 Library:NIST08.LIB
SI:75 Formula:C24H50O3S CAS:0-00-0 MolWeight:418 RetIndex:2966
CompName:Sulfurous acid, 2-ethylhexyl hexadecyl ester

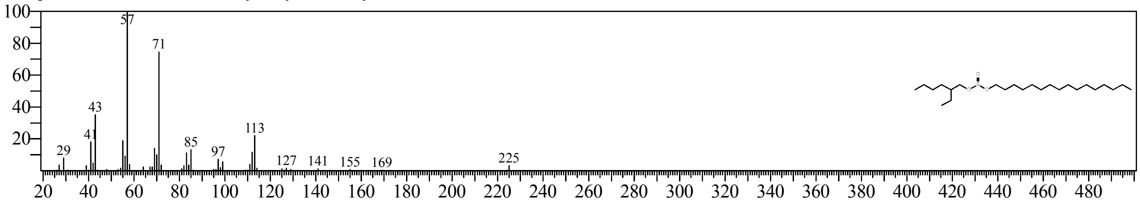

Hit#:4 Entry:165157 Library:NIST08.LIB
SI:75 Formula:C28H56 CAS:55255-73-7 MolWeight:392 RetIndex:2344
CompName:6-Tridecene, 2,2,4,10,12,12-hexamethyl-7-(3,5,5-trimethylhexyl)- $$ 2,2,4,10,12,12-Hexamethyl-7-(3,5,5-trimethylhexyl)-6-tridecene $$

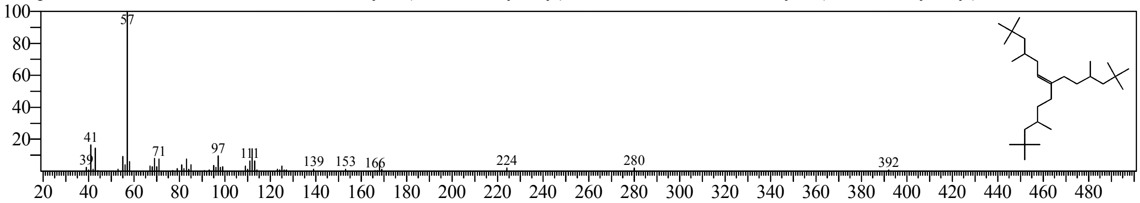

Hit#:5 Entry:178403 Library:NIST08.LIB
SI:75 Formula:C26H54O3S CAS:0-00-0 MolWeight:446 RetIndex:3165
CompName:Sulfurous acid, 2-ethylhexyl octadecyl ester

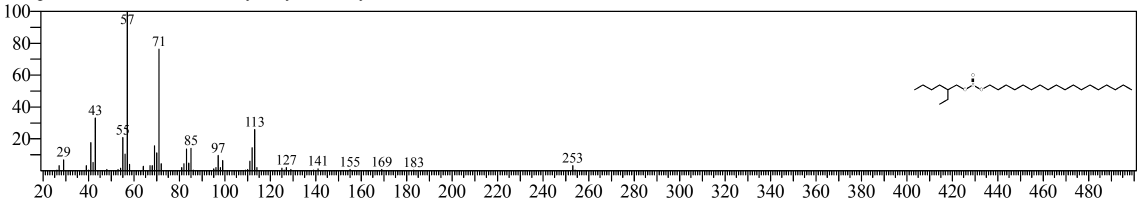

<< Target >>
Line#:27 R.Time:53.190(Scan#:9939) MassPeaks:334
RawMode:Single 53.190(9939) BasePeak:87.05(31370)
BG Mode:53.080(9917) Group 1 - Event 1

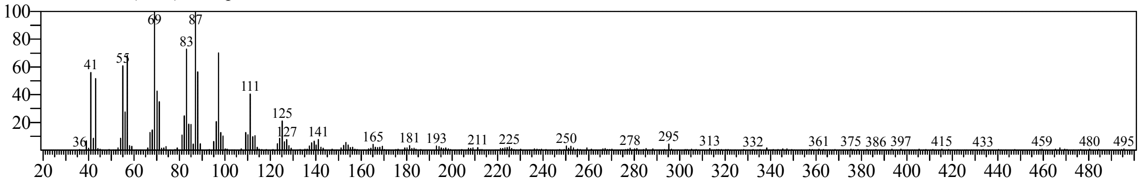

Hit#:1 Entry:128769 Library:NIST08.LIB
SI:93 Formula:C21H40O2 CAS:0-00-0 MolWeight:324 RetIndex:2243
CompName:Methacrylic acid, heptadecyl ester

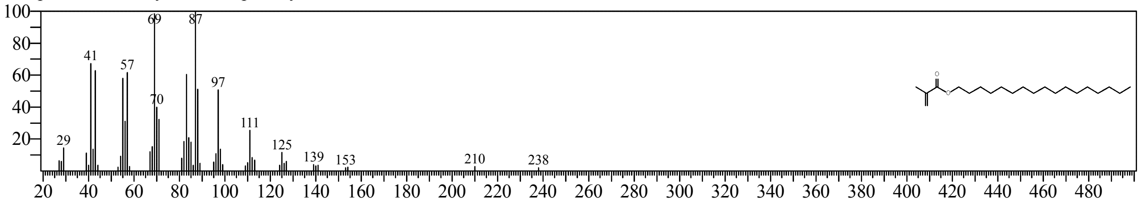

Hit#:2 Entry:146279 Library:NIST08.LIB
SI:93 Formula:C23H44O2 CAS:0-00-0 MolWeight:352 RetIndex:2442
CompName:Methacrylic acid, nonadecyl ester

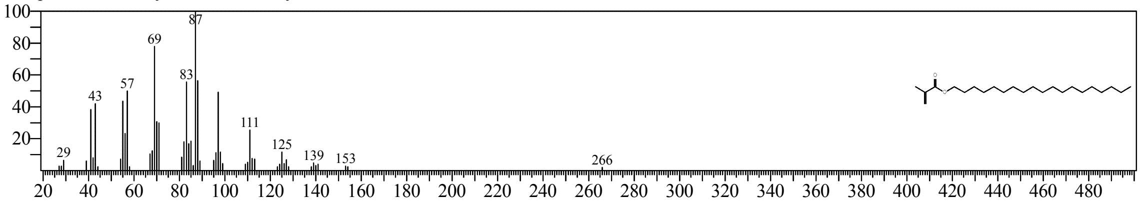

Hit#:3 Entry:119289 Library:NIST08.LIB
SI:92 Formula:C20H38O2 CAS:0-00-0 MolWeight:310 RetIndex:2144
CompName:Methacrylic acid, hexadecyl ester

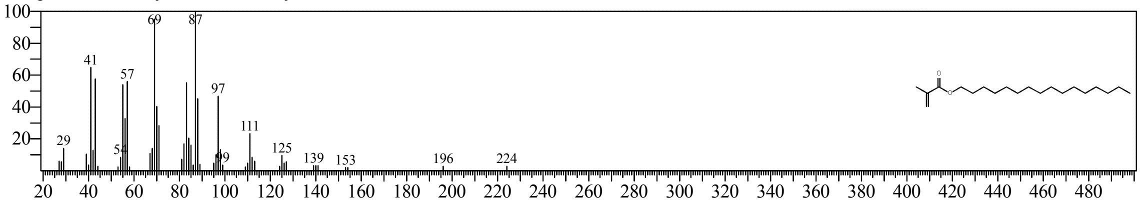

Hit#:4 Entry:137968 Library:NIST08.LIB
SI:92 Formula:C22H42O2 CAS:112-08-3 MolWeight:338 RetIndex:2343
CompName:Octadecyl methacrylate $$ Octadecyl 2-methylacrylate # $$

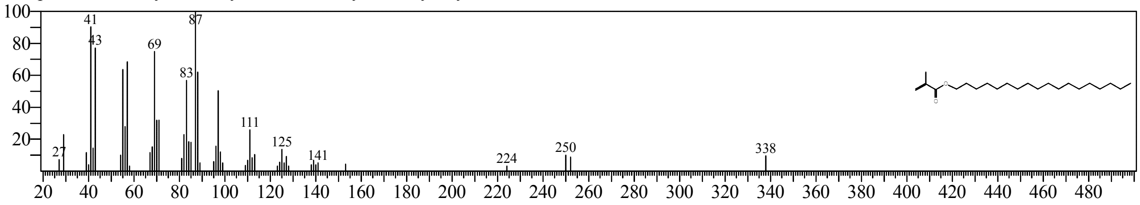

Hit#:5 Entry:109293 Library:NIST08.LIB
SI:92 Formula:C19H36O2 CAS:0-00-0 MolWeight:296 RetIndex:2044
CompName:Methacrylic acid, pentadecyl ester

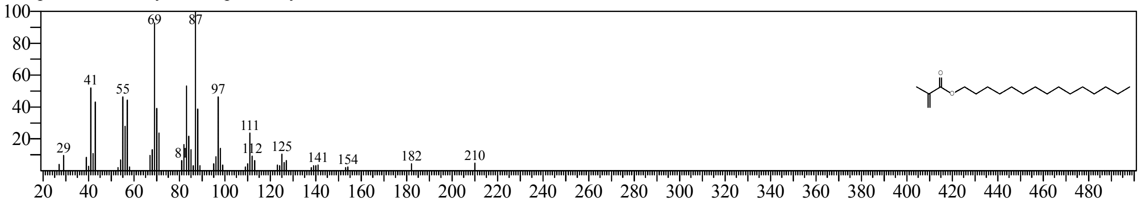

Supplement: S2 Table — (PDF) [file pone.0206982.s002.pdf]
